# Clinofibrate Disrupts the SNORA80B/YTHDC1‐Driven M6A Modification to Suppress Cholesterol Metabolism and Cisplatin Resistance in ESCC

**DOI:** 10.1002/advs.202509574

**Published:** 2025-11-03

**Authors:** Hongyu Yuan, Ge Ge, LiQiu Liu, Sijun Hu, Miaomiao Tian, Yongzhan Nie, Zitong Zhao, Yongmei Song

**Affiliations:** ^1^ State Key Laboratory of Molecular Oncology National Cancer Center/National Clinical Research Center for Cancer/Cancer Hospital Chinese Academy of Medical Sciences and Peking Union Medical College Beijing 100021 P. R. China; ^2^ State Key Laboratory of Bioactive Substance and Function of Natural Medicines Institute of Materia Medica Chinese Academy of Medical Sciences and Peking Union Medical College Beijing 100050 P. R. China; ^3^ State Key Laboratory of Holistic Integrative Management of Gastrointestinal Cancers Xijing Hospital of Digestive Diseases Fourth Military Medical University Xi'an 710032 P. R. China

**Keywords:** androgen receptor, cholesterol metabolism, esophageal squamous cell carcinoma, m^6^A modification, SNORA80B

## Abstract

Esophageal squamous cell carcinoma (ESCC) progression is driven by androgen receptor (AR) signaling, while small nucleolar RNAs (snoRNAs), classically involved in ribosomal RNA processing, are increasingly recognized for non‐classical roles in cancer. However, their function in ESCC remains unknown. This study investigates AR‐regulated snoRNAs and their mechanistic contributions to ESCC pathogenesis. SNORA80B is identified as the most AR‐responsive snoRNA with oncogenic activity by transcriptomic profiling. Beyond its classical role, SNORA80B stabilizes cholesterol metabolism transcripts via N⁶‐methyladenosine (m^6^A)‐YTHDC1, driving cholesterol/DHT accumulation and lipid droplets (LDs) formation. A feedforward loop is observed wherein DHT‐activated AR upregulates SNORA80B, which further enhances AR signaling through cholesterol metabolic reprogramming. Clinofibrate, identified as a SNORA80B inhibitor through high‐throughput screening of FDA‐approved drugs, disrupts this axis and demonstrates synergistic effects with cisplatin, overcoming resistance in ESCC. The study reveals a novel non‐classical function of SNORA80B in ESCC, establishing it as a key effector of AR‐driven metabolic reprogramming through m⁶A‐dependent regulation. The repurposing of clinofibrate demonstrates the therapeutic potential of targeting snoRNA‐mediated pathways, providing both mechanistic insights and a clinically translatable strategy for ESCC treatment. These findings redefine the functional scope of snoRNAs in cancer pathogenesis.

## Graphical abstract

This study uncovers a non‐canonical role of SNORA80B in ESCC, showing it mediates AR‐driven metabolic reprogramming via N⁶‐methyladenosine (m^6^A) methylation. By binding YTHDC1, SNORA80B stabilizes cholesterol‐metabolic enzyme mRNAs and promotes tumor progression. The FDA‐approved drug clinofibrate inhibits SNORA80B by disrupting cholesterol homeostasis, synergistically resensitizing cisplatin‐resistant ESCC to therapy.

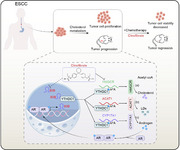

## 1 Introduction

Esophageal cancer remains a significant public health and continues to affect the social development. It ranks as the eleventh most commonly diagnosed cancer and the seventh leading cause of cancer‐related mortality worldwide, with an estimated 511000 new cases and 445000 deaths reported in 2022.^[^ ^1^ ^]^ Esophageal squamous cell carcinoma (ESCC) is the predominant histological subtype in Asia, Africa and south America, characterized by poor prognosis, primarily owing to late stage diagnosis in most patients.^[^ ^2^, ^3^ ^]^ In addition to common risk factors such as alcohol consumption, tobacco use and dietary custom, mounting evidence from various researches reveals that sex hormone signaling plays crucial role in cancer susceptibility and tumor progression.^[^ ^4^ ^]^

Sex hormone mainly includes estrogen and androgen. Emerging studies suggest that estrogen and its receptors, particularly estrogen receptor β (ERβ), may exert a protective effect by inhibiting tumor growth and metastasis.^[^ ^5^ ^]^ In contrast, androgen receptor (AR) is a ligand‐dependent transcription factor that regulates target gene expression upon activation by androgens, and is associated with a more aggressive ESCC phenotype, potentially influencing inflammatory signals, proliferation and survival.^[^ ^6^, ^7^ ^]^ Cholesterol acts as a substrate for androgen synthesis, providing the backbone of the steroid molecule. Studies suggest that aberrant cholesterol homeostasis, characterized by elevated intracellular cholesterol synthesis, uptake, and storage, promotes tumorigenesis and cancer progression by sustaining membrane biogenesis for rapid cell proliferation, migration and invasion.^[^ ^8^ ^]^ Cholesterol homeostasis is dynamically regulated by central metabolic enzymes, including 3‐hydroxy‐3‐methylglutaryl‐CoA reductase (HMGCR) catalyzing de novo biosynthesis, acyl‐coenzyme A acyltransferase 1 (ACAT1) mediating intracellular esterification, and cytochrome P450 17A1 (CYP17A1) driving steroidogenic conversion to androgens. Emerging evidence demonstrates that these enzymes critically regulate both oncogenesis and malignant progression through pathological modulation of cholesterol metabolic circuitry, revealing potential therapeutic vulnerabilities in cancer biology.^[^ ^9^ ^]^ While the oncogenic interplay between AR and cholesterol homeostasis has been comprehensively studied, it still remains unclear how AR‐mediated transcriptional reprogramming dynamically regulates the tumorigenesis and whether these transcriptional targets possess intrinsic oncogenic capacity in ESCC pathogenesis.

Small nucleolar RNAs (snoRNAs) are mainly encoded by intronic regions of both protein‐coding and non‐coding genes, and widely present in nucleus of eukaryotic cells with a length of 60–300 nucleotides. According to the structural features, snoRNAs are primarily categorized into two classes, box H/ACA snoRNAs and box C/D snoRNAs. These snoRNAs canonically direct site‐specific 2′‐O‐methylation and pseudouridylation of rRNAs through the formation of ribonucleoprotein complexes with small nucleolar riboproteins (snoRNPs).^[^ ^10^ ^]^ Beyond their canonical function, emerging evidence indicates that snoRNAs are involved in posttranscriptional regulation, such as rRNA acetylation, mRNA abundance, alternative splicing, and translation efficiency regulation.^[^ ^11^ ^]^ For instance, SNORD115 could modulate the alternative splicing of 5‐hydroxytryptamine receptor 2C pre‐mRNA.^[^ ^12^, ^13^ ^]^ In a breast cancer study, SNORA71A was identified as a critical mediator of epithelial‐mesenchymal transition (EMT)‐driven metastasis by maintaining the stabilization of ROCK2 mRNA.^[^ ^14^ ^]^ The landscape of ESCC research has expanded to include various non‐coding RNA species, with multiple studies characterizing tumor‐associated lncRNAs, circRNAs, and other regulatory RNAs.^[^ ^15^, ^16^ ^]^ Most recently, prognostic investigations established strong correlations between elevated SNORA42 or SNORD12B expression levels and unsatisfied clinical outcomes in ESCC progression.^[^ ^17^, ^18^ ^]^ Mechanistic studies further uncovered metabolic regulatory roles, exemplified by H/ACA snoRNA U17's control of cholesterol homeostasis and steroid precursor biosynthesis via esterification pathways.^[^ ^19^ ^]^ These findings collectively highlighted the imperative for systematic exploration of snoRNAs in ESCC.

YTH domain‐containing 1 (YTHDC1), a pivotal nuclear N⁶‐methyladenosine (m^6^A) reader protein, recognizesm^6^A modifications through evolutionarily conserved binding domains that specifically interact with m⁶A‐containing RNA molecules.^[^ ^20^ ^]^ Accumulating evidence has revealed that YTHDC1 orchestrates multiple post‐transcriptional regulatory mechanisms by mediating m⁶A‐dependent processes including mRNA stability control, alternative splicing modulation, and nuclear‐cytoplasmic shuttling.^[^ ^21^ ^]^ Of note, YTHDC1, as the main nuclear reader protein, has distinct function in cancers: it acts as a tumor suppressor in lung cancer by impeding malignant progression,^[^ ^22^ ^]^ yet paradoxically functions as an oncogenic factor in acute myeloid leukemia through stabilization of m⁶A‐modified transcripts.^[^ ^23^ ^]^ While recent studies have established YTHDC1's regulatory capacity over long non‐coding RNAs (lncRNAs) and circular RNAs (circRNAs) in various cancer models,^[^ ^24^, ^25^ ^]^ its mechanistic interplay with snoRNAs in ESCC development remains unexplored. This knowledge gap highlights the urgent need to investigate potential YTHDC1‐snoRNA regulatory networks that may contribute to ESCC pathogenesis through m⁶A‐mediated epigenetic mechanisms.

In the present study, we systematically characterized the AR‐associated snoRNA landscape in ESCC, leading to the functional validation of SNORA80B as a direct AR transcriptional target. Mechanistically, SNORA80B knockdown significantly impaired ESCC cell metastasis behavior through interaction with the m⁶A reader protein YTHDC1, thereby disrupting the cholesterol‐to‐androgen metabolic axis‐*a* critical steroidogenic pathway sustaining ESCC progression.

## 2 Results

### 2.1 AR‐Regulated SNORA80B Upregulation in ESCC

**Figure 1:**
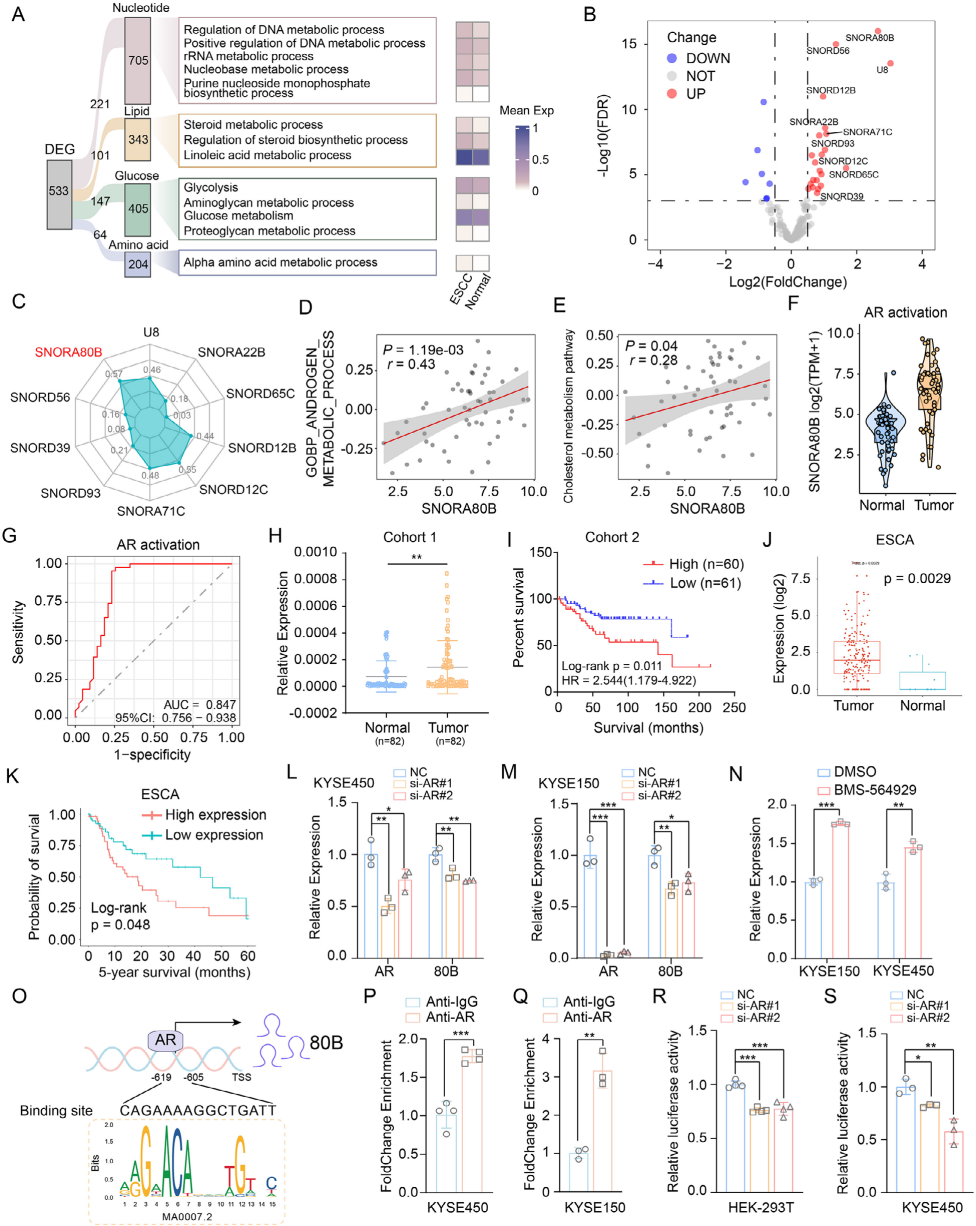
Androgen receptor (AR)‐related SNORA80B is elevated in ESCC. A) The aberrant events associated with AR activation in ESCC samples. B) The aberrant snoRNAs profiles in ESCC samples with AR activation. C) The top 10 snoRNAs with the highest correlation to cholesterol metabolism. D,E) The correlation between SNORA80B and androgen metabolic process pathway D) or cholesterol metabolic process (E). F) The expression of SNORA80B in ESCC samples with AR activation based on the ESCC transcriptome profiling. G) The ROC curve analysis of SNORA80B in ESCC. H) SNORA80B level is increased in ESCC compared to adjacent tissues in cohort 1 measured by qPCR. I) Survival analysis of ESCC patients with high and low SSNORA80B in cohort 2. J) Analysis of the expression level of SNORA80B in esophageal cancer (ESCA) acquired from the SNORic database. K) Kaplan–Meier curves for 5‐year survival of ESCA patients with high and low SNORA80B from the online SNORic database. L, M) Silencing AR inhibited the expression level of SNORA80B in KYSR450 (L) and KYSE150 (M). N) The expression level of SNORA80B was increased by BMS‐564929, an agonist of AR. O) A schematic illustration of the potential binding sites for AR located within the 1‐kb region upstream of the SNORA80B transcription sites. P,Q) Chromatin immunoprecipitation (ChIP)‐qPCR analysis showing the enrichment of AR at the genomic sites located within SNORA80B promoter in KYSE450 P) and KYSE150 Q) cells. R, S) The luciferase signal in 293T R) or KYSR450 S) cells with AR silencing is determined by luciferase reporter assays. The data are presented as the means ± SEM (*n* ≥ 3). A two‐tailed Student's *t*‐test was performed to compare the two groups. ^*^ *p* < 0.05, ^**^ *p* < 0.01, ^***^ *p* < 0.001.

In the transcriptome sequencing data of ESCC samples, we found that the pathway of androgen synthesis exhibited a notable bimodal distribution, with the right side of the dashed line indicating AR activation (Figure S1A, Supporting Information). AR, a member of the nuclear receptor superfamily, extends beyond its canonical role in the reproductive system to critically orchestrate tumor metabolic reprogramming. Interestingly, the metabolic pathway, including glucose, lipid, amino acid and nucleotide metabolism, were dysregulated in ESCC samples with AR activation (**Figure** **1**A). Further analysis in ESCC samples with AR activation revealed the aberrant profile of snoRNAs (Figure 1B). Considering that the synthetic precursor of AR is cholesterol, a type of steroid, the correlations of top 10 snoRNAs with cholesterol metabolism were analyzed. The result showed SNORA80B was most closely associated with cholesterol metabolism (Figure 1C). Additionally, the correlation between SNORA80B and AR metabolism or cholesterol metabolism was analyzed in ESCC samples with AR activation. The results indicated a strong correlation between SNORA80B expression and androgen metabolic process or cholesterol metabolism pathway (Figure 1D,E). The expression of SNORA80B in ESCC samples with AR activation was obviously increased in tumors compared with normal tissues (Figure 1F). ROC curve analysis demonstrated that the expression level of SNORA80B possessed certain diagnostic value for ESCC (Figure 1G). Data from 82 pairs of ESCC samples showed that SNORA80B was highly expressed in ESCC tissues (Figure 1H). To validate the prognostic role of SNORA80B, we assessed a second cohort and observed that higher SNORA80B expression was associated with poor prognosis and metastasis in patients with ESCC (Figure 1I). Moreover, data from SNORic database (http://bioinfo.life.hust.edu.cn/SNORic/basic/) in esophageal cancer indicated that SNORA80B were significantly higher in tumor tissues than adjacent esophageal tissues (Figure 1J). Furthermore, among patients with esophageal cancer, those with high SNORA80B expression exhibited shorter overall survival than those with low SNORA80B expression (Figure 1K). Therefore, the expression of SNORA80B was up‐regulated upon AR activation, and correlated with poor prognosis in ESCC.

To investigate whether SNORA80B was regulated by AR, the protein expression of AR in ESCC cells was detected first (Figure S1B, Supporting Information). Thus, KYSE450 and KYSE150 cells were transfected with siRNA targeting AR, which revealed that the expression of SNORA80B was inhibited when AR was reduced (Figure 1L,M). Moreover, the expression level of SNORA80B was also elevated in ESCC cells following treatment with the AR agonist BMS‐564929 (Figure 1N). To validate the functional relationship within the AR/SNORA80B axis, we performed a series of rescue experiments. RTCA and Transwell assays demonstrated that overexpression of SNORA80B effectively rescued the tumor‐suppressive effects resulting from AR knockdown, promoting proliferation, migration, and invasion in ESCC cells (Figure S1C–E, Supporting Information). Conversely, knockdown of SNORA80B attenuated the tumor‐promoting effects induced by BMS‐564929, an AR agonist (Figure S1F–H, Supporting Information).

To determine whether AR directly regulated SNORA80B expression, we analyzed the promoter region of SNORA80B using JASPAR database (https://jaspar.elixir.no/). The results from the online tools suggested the presence of AR binding sites in the SNORA80B promoter (Figure 1O). ChIP assays were carried out and results confirmed the binding between AR and SNOAR80B promoter region (Figure 1P,Q). Moreover, to explore the transcriptional activity of SNORA80B by AR, dual‐luciferase reporter assay revealed that the luciferase activity was decreased markedly in AR down‐regulated cells (Figure 1R,S). Collectively, the results above proved that SNORA80B was transcriptionally regulated by AR in ESCC.

### 2.2 SNORA80B Enhanced Malignant Phenotypes of ESCC Cells In Vitro and In Vivo

**Figure 2:**
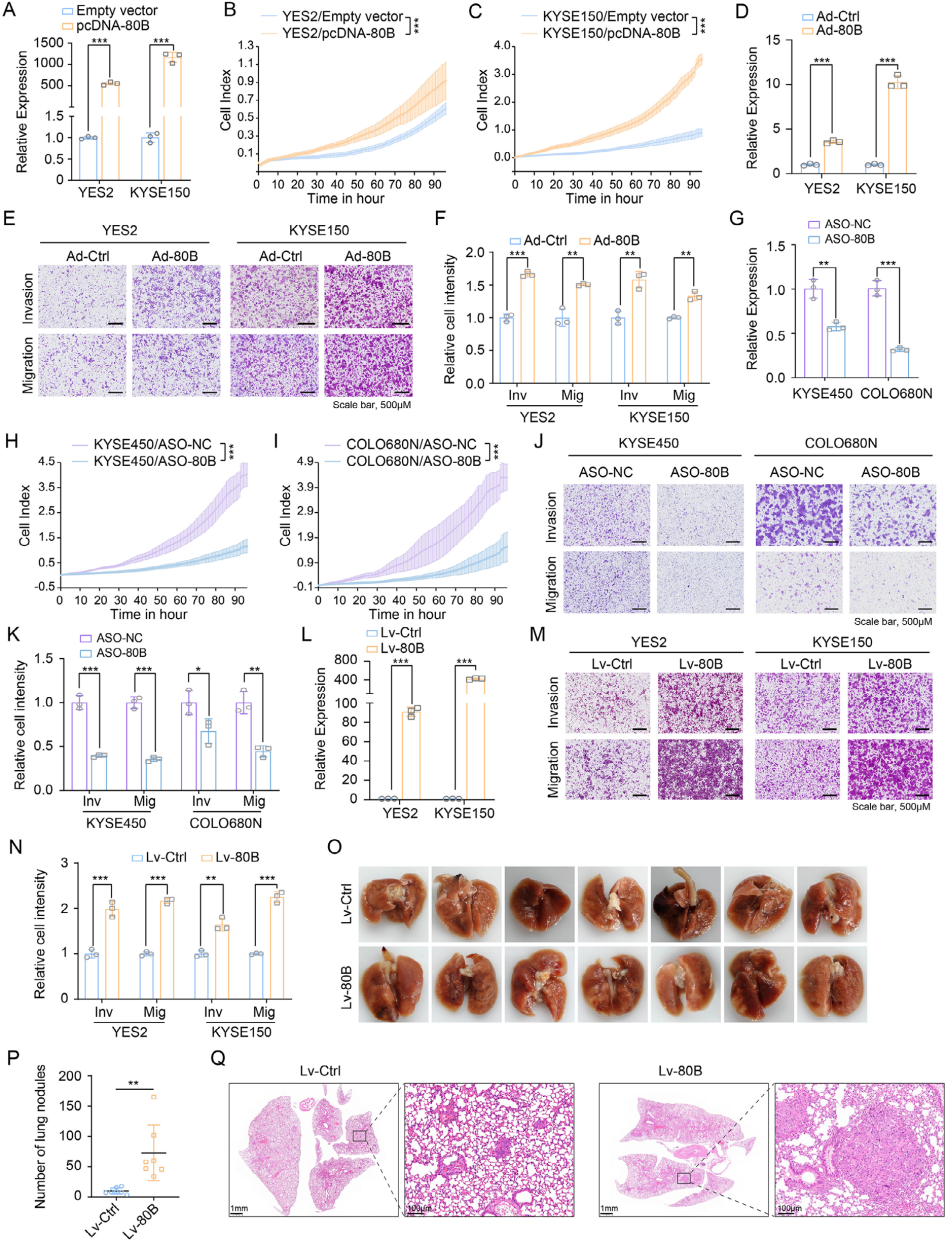
SNORA80B promotes malignant phenotype of ESCC cells in vitro and in vivo. A) The overexpressed efficiency in YES2 and KYSE150 cells transfected with SNORA80B plasmid. B,C) RTCA assays showing the effects of SNORA80B overexpression by plasmid on ESCC cell proliferation. D) qPCR analysis for the overexpression levels of adenovirus‐SNORA80B in YES2 and KYSE150 cells. E, F) Transwell assays showing the effects of SNORA80B overexpression on ESCC cell migration and invasion. G) The knockdown efficiency of ASO targeting SNORA80B confirmed by qPCR. H,I) RTCA assays showing the effects of SNORA80B knockdown by ASO on ESCC cell proliferation. J, K) Transwell assays showing the effects of SNORA80B knockdown on ESCC cell migration and invasion. L) The efficiencies of SNORA80B overexpression stably in YES2 and KYSE150 cells detected by qPCR. M, N) Transwell assays indicating the effect of SNORA80B overexpression on migration and invasion. O) The lung of mice after tail vein injection of KYSE150‐Lv‐Ctrl or KYSE150‐Lv‐80B cells. P) The quantification of metastatic nodules in the lung. Q) H&E staining showing the metastatic nodules in the lung tissues. The data are presented as the means ± SEM (*n* ≥ 3). A two‐tailed Student's *t*‐test was performed to compare the two groups. ^*^ *p* < 0.05, ^**^ *p* < 0.01, ^***^ *p* < 0.001.

We first profiled the basal expression of SNORA80B and its host gene, ODC1, across a panel of 10 ESCC cell lines. (Figure S2A,B, Supporting Information). The linear correlation analysis was conducted and showed that no correlation, R square = 0.0148 and p = 0.7376 (Figure S2C, Supporting Information). To determine whether SNORA80B was expressed independently of its host gene, we examined the effect of AR knockdown on ODC1 expression and found that it was not significantly altered in ESCC cells (Figure S2D,E, Supporting Information). Therefore, SNORA80B was over‐expressed in ESCC independent of ODC1. With the purpose of exploring the cellular function of SNORA80B, we conducted gain‐ and loss‐of‐function studies. The results of phenotype experiments revealed that SNORA80B overexpression in YES2 and KYSE150 cells significantly promoted cell proliferation, migration and invasion of ESCC cells (**Figure** **2**A–F). Conversely, antisense oligonucleotides (ASOs)‐mediated knockdown of SNORA80B substantially suppressed the ability of cell proliferation, migration and invasion of KYSE450 and COLO680N cells (Figure 2G–K). Taken together, this approach revealed that SNORA80B was an oncogene required for malignant phenotypes in ESCC cells.

To investigate the biological functions of SNORA80B in ESCC in vivo, YES2 and KYSE150 cells were stably infected with lentiviral negative control (Lv‐Ctrl) or SNORA80B lentiviral vectors (Lv‐80B). qRT‐PCR was applied to detect the efficiency of overexpression (Figure 2L). In addition, Transwell assay was further performed and the results showed high ability of invasion and migration in Lv‐SNORA80B cells compared with Lv‐Ctrl cells (Figure 2M,N), which was consistent with previous results. To determine whether SNORA80B enhances tumor metastasis in vivo, we injected BALB/c nude mice with Lv‐Ctrl or Lv‐80B cells via the tail vein. The resulting experimental metastasis assay demonstrated that SNORA80B overexpression significantly increased the number of lung metastases compared to the control group, corroborating our in vitro findings (Figure 2O–Q). qPCR analysis revealed that the expression of SNORA80B was significantly elevated in lung nodules from SNORA80B‐overexpressing groups compared with the control groups (Figure S2F, Supporting Information). Altogether, these results suggested that SNORA80B could enhance the invasion, migration and metastasis abilities of ESCC cells in vitro and in vivo.

### 2.3 SNORA80B Interacted with YTHDC1

**Figure 3:**
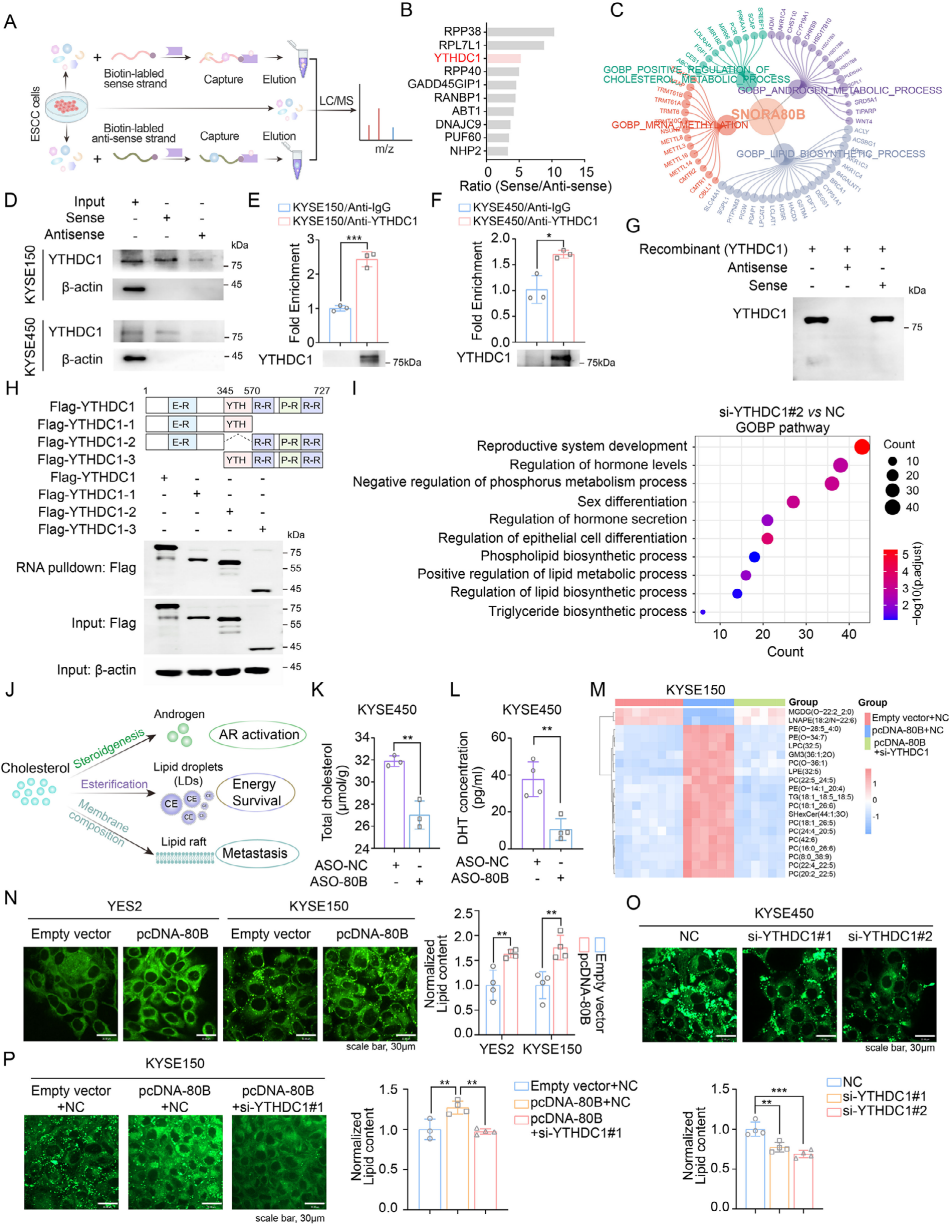
SNORA80B regulates cholesterol homeostasis in ESCC cells. A) Schematic of RNA‐protein pull‐down in ESCC cell lysates in combination with mass spectrometry analysis. B) Ten most abundant SNORA80B binding proteins identified by mass spectrometry. YTHDC1 is highlighted in red. C) The aberrant events associated with SNORA80B in ESCC. D) RNA pull‐down assays showing the interaction between SNORA80B and YTHDC1 in ESCC cells. β‐actin was used as a negative control. E, F) RIP and western blot assays showing YTHDC1 binding with SNORA80B in KYSE150 (E) and KYSE450 (F) cells. G) The physical interaction between SNORA80B and YTHDC1 was detected by recombinant protein of YTHDC1. H) The RNA pull‐down assays showing the interaction of SNORA80B with the YTHDC1 domain using ESCC cells transfected with truncated YTHDC1 plasmids. I) The differentially expressed genes (DEGs) pathway enrichment analysis by RNA sequencing in KYSE450 cells with si‐YTHDC1 vs si‐NC cells. J) Schematic diagram of cholesterol transport pathways. K) Cholesterol detection showing accumulation of total cholesterol in silencing SNORA80B compared with negative control cells. L) The DHT levels in SNORA80B knockdown and negative control groups. M) Heatmap of lipid‐related metabolites in the indicated cells. N) Representative confocal images and quantification of BODIPY in ESCC cells with overexpression SNORA80B vs negative control cells. O) Lipid droplet staining by BODIPY and quantification of lipid content in ESCC cells with si‐YTHDC1 vs si‐NC cells. P) Lipid droplet staining by BODIPY and quantification of lipid content showing silencing YTHDC1 attenuated the lipid droplet formation increased by SNORA80B overexpression. The data are presented as the means ± SEM (*n* ≥ 3). A two‐tailed Student's *t*‐test was performed to compare the two groups. One‐way ANOVA was used for multiple groups. ^*^ *p* < 0.05, ^**^ *p* < 0.01, ^***^ *p* < 0.001.

To further analyze the functions and mechanism of SNORA80B in ESCC, subcellular fractionation localization assays indicated that SNORA80B was mainly located in the nuclear (Figure S3A, Supporting Information). Studies have shown that snoRNAs exert diverse functions by forming RNA‐protein complexes with proteins.^[^ ^10^ ^]^ Therefore, RNA pull‐down assays in KYSE150 and KYSE450 cells were conducted. Cell lysates were incubated with biotin‐labeled sense‐SNORA80B or antisense‐SNORA80B, and further captured by Streptavidin beads. The proteins eluted from Streptavidin beads were identified by mass spectrometry (**Figure** **3**A). According to the results of protein mass spectrometry analysis, 10 highly enriched proteins were screened in ESCC cells (the ratio of sense/antisense and sense/beads >1.5) (Figure 3B). The biological events associated with SNORA80B were analyzed using transcriptome sequencing data of ESCC samples, which suggested that higher SNORA80B expression was related to androgen metabolic process, RNA methylation pathway, lipid metabolism and cholesterol metabolic process (Figure 3C). Among the binding proteins, YTHDC1 is an m^6^A reader protein with a localization exclusive to the nucleus, and has been implicated in the regulation of splicing, RNA stability and RNA export.^[^ ^26^, ^27^, ^28^ ^]^ Based on the previous studies, we proposed that YTHDC1 served as a primary interactor of SNORA80B through its m^6^A‐binding activity, orchestrating the post‐transcriptional regulatory programs to drive oncogenic process in ESCC.

The interaction between SNORA80B and YTHDC1 was confirmed by RNA pull‐down and western blots in KYSE150 and KYSE450 cells (Figure 3D). Subsequent RIP assays also revealed the direct interaction between YTHDC1 and SNORA80B (Figure 3E,F). In addition, the physical interaction between YTHDC1 and SNORA80B was validated using purified recombinant YTHDC1 protein (Figure 3G). We then constructed three truncated mutants of YTHDC1 to further identify the domains of YTHDC1 that mediated the interaction with SNORA80B. RNA pull down assays in 293T cells transfected with full‐length YTHDC1 and three truncated mutants showed differential interactions with SNORA80B (Figure 3H). Collectively, these results indicated that SNORA80B interacted with YTHDC1. To further investigate the regulation between SNORA80B and YTHDC1, we established cell models of SNORA80B overexpression or inhibition. The results suggested up or down regulation of SNORA80B had no effect on both mRNA and protein levels of YTHDC1 (Figure S3B–G, Supporting Information). Additionally, knockdown YTHDC1 did not change the expression of SNORA80B (Figure S3H–J, Supporting Information). Moreover, in vitro experiments suggested YTHDC1 inhibition decreased the abilities of cell proliferation, colony formation, invasion and migration in ESCC (Figure S4, Supporting Information), indicating the oncogenic role of YTHDC1 in ESCC.

### 2.4 SNORA80B Regulated Lipid Droplet Formation Through YTHDC1

In the above results, the regulation between SNORA80B and YTHDC1 was not observed, thus we proposed the interaction between them might affect the enzyme activity of YTHDC1. To further determine the downstream pathway regulated by SNORA80B/YTHDC1 axis, the RNA sequencing in YTHDC1 knockdown or control cells was conducted. The differential expressed genes (DEGs) were enriched in hormone secretion, lipid metabolic pathway and triglyceride biosynthetic process (Figure 3I). Combined with the previous analysis in Figure 3C, the results implicated SNORA80B/YTHDC1 might regulate cholesterol metabolism in ESCC. Cholesterol functions tripartitely as: 1) a critical precursor for de novo androgen synthesis to initiate AR signaling; 2) a substrate for esterification sequestered as lipid droplets (LDs) for energy homeostasis; and 3) a structural component integrated into membranes (Figure 3J). Additionally, elevated levels of total cholesterol and dihydrotestosterone (DHT) in control group were mitigated by the SNORA80B knockdown (Figure 3K,L). Furthermore, upregulation of SNORA80B increased the DHT concentration in KYSE150 cells (Figure S5A, Supporting Information), while inhibition of YTHDC1 decreased the total cholesterol levels in KYSE450 cells (Figure S5B, Supporting Information). To investigate the metabolic alterations underlying SNORA80B and YTHDC1, we conducted Liquid chromatography‐coupled tandem mass spectrometry (LC‐MS/MS)‐based analysis followed by unsupervised hierarchical clustering. The LC‐MS/MS results revealed distinct metabolite profiles in SNORA80B‐overexpressing cells compared to negative controls, and these alterations were attenuated upon YTHDC1 knockdown (Figure 3M). Additionally, the concentration of triacylglycerol (TG) was elevated in SNORA80B‐overexpressing cells, but this effect was reversed upon YTHDC1 knockdown (Figure S5C, Supporting Information). Besides being a precursor for steroid hormones, esterified cholesterol is the main component of LDs. As shown in Figure 3N, the result of BODIPY (493/503) staining revealed that SNORA80B overexpression enhanced the formation of LDs, while knockdown SNORA80B decreased the formation of LDs (Figure S5D, Supporting Information). Consistently, inhibition of YTHDC1 significantly attenuated the LDs accumulation (Figure 3O), an effect that completely rescued the SNORA80B overexpression‐induced lipogenic phenotype (Figure 3P). The results highlighted the crucial role of SNORA80B in pathways related to cholesterol metabolism and lipid accumulation.

### 2.5 SNORA80B Mediated m^6^A Modification of the Cholesterol Metabolism Transcripts Dependent of YTHDC1

**Figure 4:**
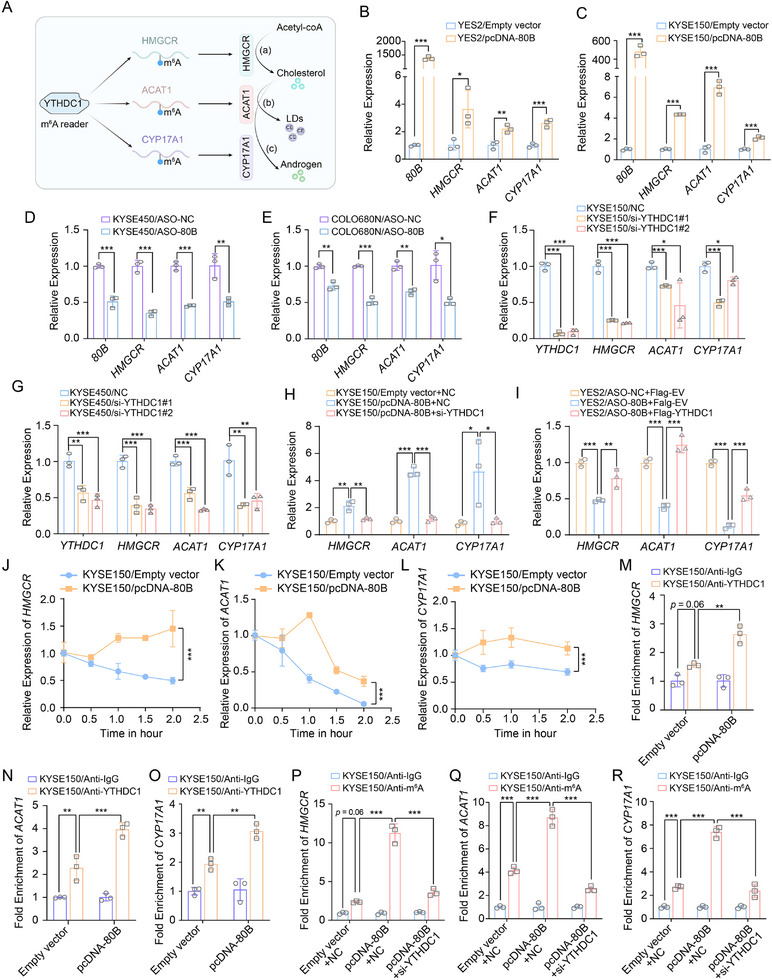
SNORA80B maintains the mRNA stability of genes associated with cholesterol homeostasis through YTHDC1 in an m^6^A dependent manner. A) Schematic illustration of the axis of SNORA80B/YTHDC1 regulating the process from cholesterol homeostasis through the m^6^A modification of HMGCR, ACAT1 and CYP17A1. B, C) qPCR analysis showing the increased mRNA levels of HMGCR, ACAT1 and CYP17A1 after overexpression SNORA80B in YES2 (B) and KYSE150 (C) cells. D, E) qPCR assays showing the effects of SNORA80B knockdown on the mRNA levels of HMGCR, ACAT1 and CYP17A1 in KYSE450 D) and COLO680N (E) cells. F, G) Effects of YTHDC1 knockdown on the mRNA expression of HMGCR, ACAT1 and CYP17A1 in KYSE150 (F) and KYSE450 (G) cells through qPCR analysis. H) The mRNA expression of HMGCR, ACAT1 and CYP17A1 in ESCC cells co‐transfected with SNORA80B and YTHDC1 siRNA. I) The mRNA expression of HMGCR, ACAT1 and CYP17A1 in ESCC cells co‐transfected with ASO‐80B and YTHDC1 plasmid. J–L) The mRNA stability of HMGCR J), ACAT1 K) and CYP17A1 L) by actinomycin D inhibition in KYSE150 cells with SNORA80B overexpression. M–O) RIP‐qPCR analysis the binding ability of YTHDC1 with HMGCR, ACAT1, CYP17A1 in KYSE150 cell line with SNORA80B overexpression by YTHDC1 antibody. IgG was used as a negative control. P–R) m^6^A methylation levels of HMGCR, ACAT1, CYP17A1 in KYSE150 cell line transfected with a empty vector or SNORA80B and either NC or si‐YTHDC1 by meRIP assays. The data are presented as the means ± SEM (*n* ≥ 3). A two‐tailed Student's *t*‐test was performed to compare the two groups. One‐way ANOVA and two‐way ANOVA were used for multiple groups. ^*^ *p* < 0.05, ^**^ *p* < 0.01, ^***^ *p* < 0.001.

Cholesterol biosynthesis initiates with the condensation of two acetyl‐CoA molecules catalyzed by HMGCR, the primary rate‐limiting enzyme in cholesterol biosynthesis. To maintain cellular homeostasis, cholesterol in excess of the current cellular demand could be either converted to less toxic LDs by ACAT1 or used as a precursor for androgen production by CYP17A1. As YTHDC1 is established as an m^6^A reader protein, we systematically identified conserved m^6^A methylation motifs in the transcripts of HMGCR, ACAT1, and CYP17A1, demonstrating their functional significance in post‐transcriptional regulation of gene expression. Thus, we proposed YTHDC1 enhanced the stability of HMGCR, ACAT1 and CYP17A1 in the m^6^A dependent manner to promote cholesterol metabolism (**Figure** **4**A). To verify the regulation of HMGCR, ACAT1 and CYP17A1 by SNORA80B/YTHDC1 axis, we performed qRT‐PCR and western blots on ESCC cells after SNORA80B overexpression or inhibition. Forced expression of SNORA80B resulted in an increase both in the mRNA and protein levels of HMGCR, ACAT1 and CYP17A1 (Figure 4B,C; Figure S5E, Supporting Information), of which the expressions were reduced after SNORA80B inhibition (Figure 4D,E; Figure S5F, Supporting Information). Additionally, knockdown YTHDC1 also significantly decreased HMGCR, ACAT1 and CYP17A1 expression at both mRNA and protein levels (Figure 4F,G; Figure S5G, Supporting Information). Further rescue experiments demonstrated that knockdown of YTHDC1 attenuated the upregulation of HMGCR, ACAT1, and CYP17A1 induced by SNORA80B overexpression (Figure 4H). Conversely, overexpression of YTHDC1 restored the expression of these genes following suppression by SNORA80B knockdown (Figure 4I). Regarding the alterations at the RNA level, we next tested the mRNA stability of CYP17A1, as assessed by qRT‐PCR, which results indicated upregulation of SNORA80B increased the mRNA stability of HMGCR, ACAT1 and CYP17A1 (Figure 4J–L), while SNORA80B inhibition decreased its stability (Figure S5H–J, Supporting Information). Collectively, these results indicated that SNORA80B modulated the expression of HMGCR, ACAT1, and CYP17A1 in a YTHDC1‐dependent manner at the post‐transcriptional level.

Given that the mRNA sequence of HMGCR, ACAT1 and CYP17A1 exists m^6^A modification sites, we next examined whether SNORA80B regulated their expressions through YTHDC1 in an m^6^A dependent manner. First, we used RIP qRT‐PCR and confirmed that YTHDC1 had significant interaction with HMGCR, ACAT1 and CYP17A1 (Figure S5K,L, Supporting Information). To further determine whether SNORA80B is responsible for recruiting YTHDC1 to m^6^A sites, we conducted RIP assays with an anti‐YTHDC1 antibody. The results demonstrated that overexpression of SNORA80B markedly enhanced the enrichment of YTHDC1 at the m^6^A sites of HMGCR, ACAT1, and CYP17A1 transcripts (Figure 4M–O). In contrast, knockdown of SNORA80B significantly impaired the binding of YTHDC1 to these sites (Figure S5M–O, Supporting Information). We next asked whether the observed recruitment of YTHDC1 corresponded to changes in m^6^A modification levels. Consistent with the RIP data, m^6^A modification analysis showed that overexpression of SNORA80B significantly increased m^6^A modification on these cholesterol metabolism transcripts, an effect that was attenuated upon YTHDC1 knockdown (Figure 4P–R). Conversely, knockdown of SNORA80B reduced the modification level, which was restored by YTHDC1 overexpression (Figure S5P–R, Supporting Information). To further evaluate the expression of the cholesterol metabolism‐related transcripts in vivo, the IHC staining was performed using lung metastases tissues from SNORA80B overexpression xenografts. In line with the in vitro results, IHC staining showed that SNORA80B overexpression increased the expression of HMGCR, ACAT1 and CYP17A1, but had no significant effect on YTHDC1 expression (Figure S5S, Supporting Information). Taken together, SNORA80B enhanced the recruitment of YTHDC1 to the m^6^A sites of HMGCR, ACAT1 and CYP17A1, and then increased their expressions, thereby regulating the cholesterol metabolism.

### 2.6 Screening of Clinofibrate as a Potent Inhibitor of SNORA80B

**Figure 5:**
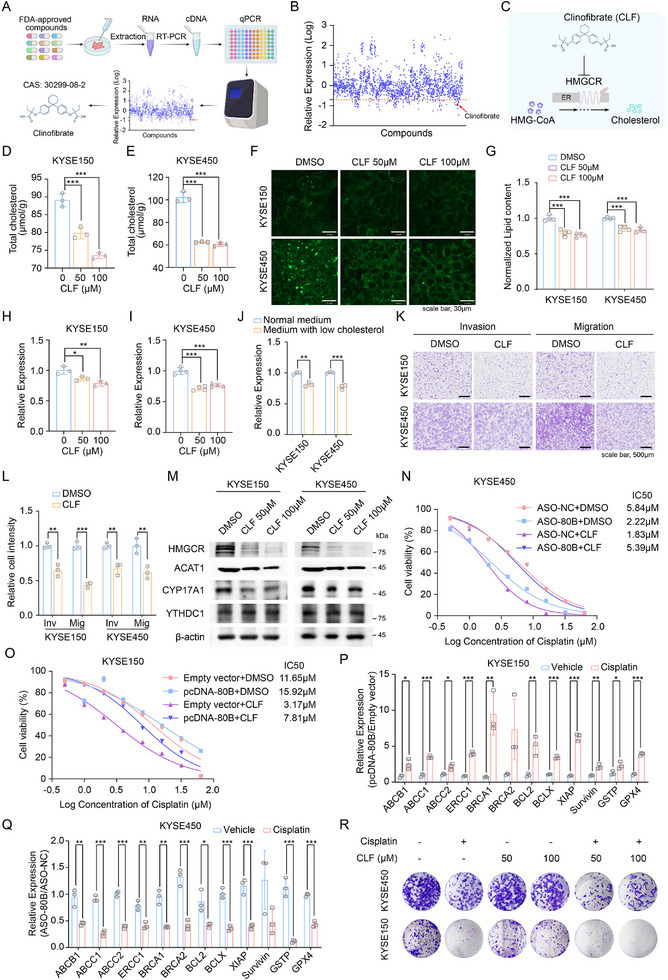
The inhibitor for SNORA80B was screened and verified its function in ESCC cells. A) Schematic illustration of the screening process to search for compounds which inhibited the expression of SNORA80B. B) The screening results of SNORA80B inhibitor through 1953 FDA‐approved drugs. C) Schematic of clinofibrate functions. D, E) Total cholesterol levels in KYSE150 (D) and KYSE450 (E) cells treated by clinofibrate. F, G) LDs staining by BODIPY (F) and quantification of lipid content (G) showing clinofibrate attenuated the LDs formation. H, I) The expression of SNORA80B in clinofibrate‐treated ESCC cells. J) The expression of SNORA80B treated by medium with low cholesterol for 4 h. K, L) Transwell assays showing the effects of clinofibrate on ESCC cell migration and invasion. M) The protein expression downstream of SNORA80B in clinofibrate‐treated ESCC cells. N) The percent viability (CCK‐8 assay) of KYSE450 cells transfected with ASO‐80B after 72 h treatment with indicated doses of cisplatin and constant treatment with CLF (100 µm) or control DMSO. O) The percent viability (CCK‐8 assay) of KYSE150 cells transfected with pcDNA‐80B after 72 h treatment with indicated doses of cisplatin and constant treatment with CLF (100 µm) or control DMSO. P) The expressions of cisplatin‐resistance related genes in KYSE150 cells transfected with pcDNA‐80B or empty vector, following treatment with cisplatin and vehicle. Q) The expressions of cisplatin‐resistance related genes in KYSE450 cells transfected with ASOA‐80B or ASO‐NC, following treatment with cisplatin and vehicle. R) Colony formation showing the combination effects of indicated doses of clinofibrate and cisplatin (0.5 µm) on ESCC cell viability. The data are presented as the means ± SEM (*n* ≥ 3). A two‐tailed Student's *t*‐test was performed to compare the two groups. ^*^ *p* < 0.05, ^**^ *p* < 0.01, ^***^ *p* < 0.001.

To further screening inhibitor of SNORA80B, we performed high throughput screening via qRT‐PCR using a library of 1,953 compounds that the Food and Drug Administration (FDA) approved (**Figure** **5**A,B). From the top 50 compounds with high inhibition to SNORA80B, we investigated the specific biological targets of these compounds (table S1, Supporting Information). Among these candidates, clinofibrate was identified as a potent suppressor of SNORA80B. Clinofibrate is an FDA‐approved drug used clinically to treat hyperlipidemia through direct inhibition of HMGCR, a rate‐limiting enzyme in cholesterol synthesis (Figure 5C). Notably, clinofibrate exhibited inhibitory effects on cholesterol content (Figure 5D,E), LDs formation (Figure 5F,G) and SNORA80B expression (Figure 5H,I) in KYSE150 and KYSE450 cells. Consistent with the cholesterol‐lowering effect of clinofibrate, SNORA80B expression was also reduced under low‐cholesterol culture conditions (Figure 5J). As a result, we focused on clinofibrate for further studies. Transwell assay suggested that clinofibrate obviously inhibited the ability of migration and invasion in ESCC cells (Figure 5K,L), consistent with the results of SNORA80B inhibition. Additionally, similar to SNORA80B inhibition, clinofibrate attenuated the expression of cholesterol metabolic proteins in ESCC cells (Figure 5M). Collectively, our results demonstrated that clinofibrate disrupted the positive feedback loop involving AR‐mediated SNORA80B transcription and YTHDC1‐dependent stabilization of HMGCR mRNA, thereby providing a novel mechanistic basis for its anticancer effects.

Cisplatin‐based chemotherapy followed by surgery has been considered a standard treatment for ESCC patients at advanced stage. However, a plethora of patients suffer from cisplatin resistance and result in the treatment failure, highlighting the need for novel therapeutic strategies. Thus, we investigated the potential role of SNORA80B inhibitor in improving cisplatin sensitivity. While both SNORA80B knockdown and CLF treatment individually sensitized cells to cisplatin (lower IC50), the knockdown of SNORA80B abolished the synergistic sensitization effect of CLF, resulting in a higher IC50 under combination treatment (Figure 5N). Conversely, SNORA80B overexpression enhanced the synergistic effect of CLF and cisplatin (Figure 5O). A consistent trend was observed when measuring the IC50 of CLF under fixed cisplatin concentration while modulating SNORA80B expression (Figure S6A,B, Supporting Information). Thus, suppressing SNORA80B expression weakens the synergistic effect of the combination treatment, whereas enhancing its expression promotes synergy. Regarding cisplatin resistance, several mechanisms have been described in tumors: 1) reduced intracellular accumulation via impaired influx or increased efflux, mediated by transporters such as ABCB1, ABCC1 and ABCC2; 2) augmented DNA repair capacity, facilitated by factors including ERCC1, BRCA1 and BRCA2; 3) dysregulation of apoptosis and activation of pro‐survival signaling pathways, involving anti‐apoptotic proteins such as Bcl2, BCLX, XIAP and Survivin; 4) enhanced detoxification, mediated by enzymes such as GSTP and GPX4. As shown in Figures 5P and S6C (Supporting Information), overexpression of SNORA80B in ESCC cells upregulated the expression of genes associated with cisplatin resistance. Conversely, knockdown of SNORA80B downregulated their expression (Figure 5Q; Figure S6D, Supporting Information). Additionally, clinofibrate attenuated colony‐formation of KYSE450 and KYSE150 cells in the presence of cisplatin (Figure 5R). These findings collectively indicated that clinofibrate exhibited inhibition of SNORA80B, thereby enhancing the sensitivity to cisplatin in ESCC cells.

### 2.7 Clinofibrate Exhibited Anti‐ESCC Efficacy and Enhancing Sensitivity to Chemotherapy In Vivo

**Figure 6:**
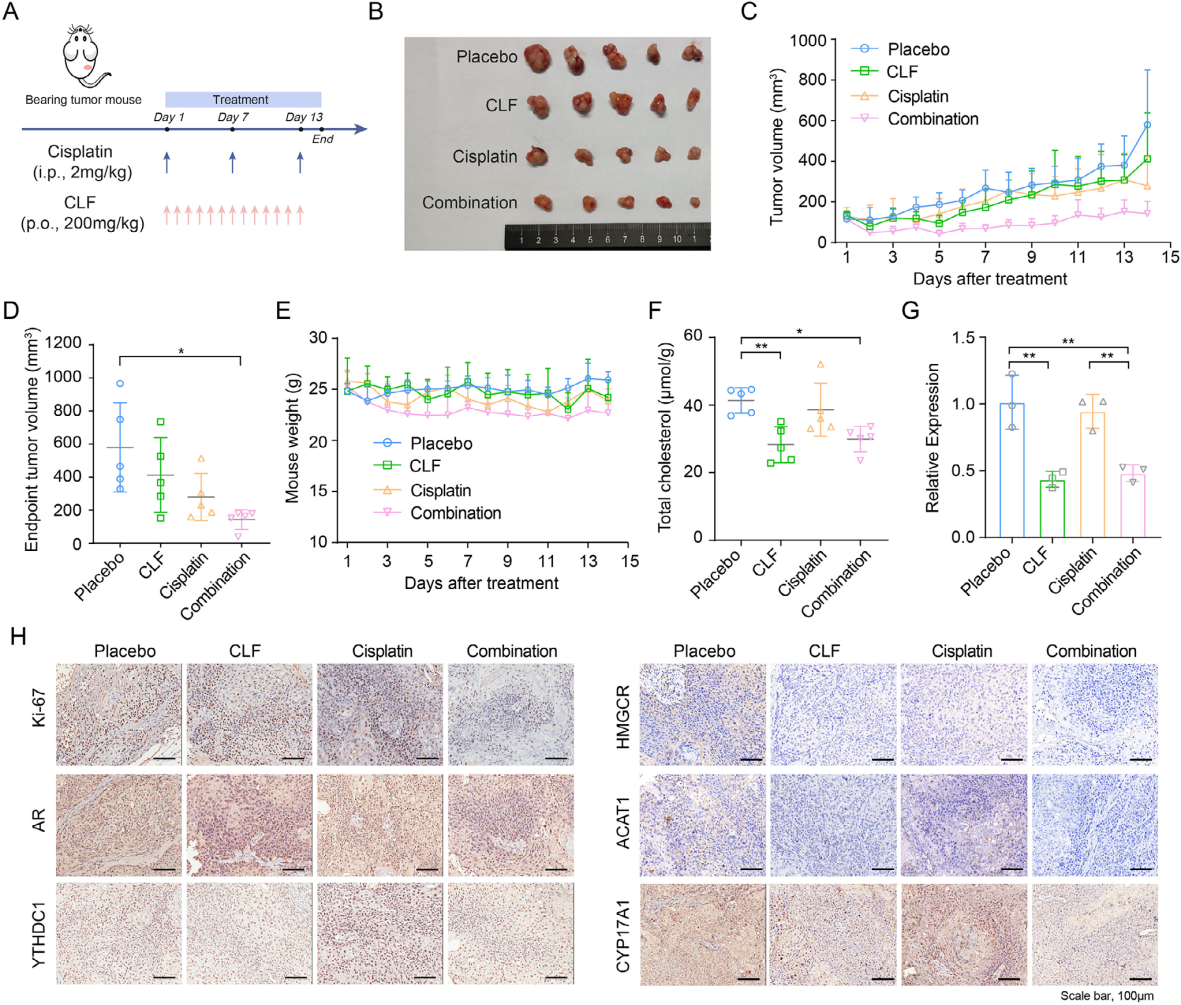
Effects of clinofibrate and cisplatin decreases the tumor growth in KYSE450 xenograft model. A) Schematic of mouse studies. B) Representative image of each group at the end of treatment. C) The curves of tumor volume for each group. D) The tumor volume of each group at the end of the experiment. E) The curves of mouse weight for each group during the whole treatment. F) The content of cholesterol in tumor tissues. G) The expression of SNORA80B in tumor xenografts. H) Representative images of IHC staining in tumor xenografts. The data are presented as the means ± SEM (*n* ≥ 3). One‐way ANOVA was used for multiple groups. ^*^ *p* < 0.05, ^**^ *p* < 0.01, ^***^ *p* < 0.001.

To better achieve clinical transformation and investigate inhibiting SNORA80B to restore cisplatin sensitivity in ESCC cells, we applied clinofibrate, the potential inhibitor of SNORA80B, combined with cisplatin in vivo experiment. Based on the previous results, we chose KYSE450 cells with high SNORA80B expression and moderate AR level for in vivo experiment. KYSE450 cells were implanted subcutaneously into BALB/c nude mice, randomly dividing into four groups, including i) placebo, ii) cisplatin, iii) clinofibrate and iv) combination (**Figure** **6**A). The in vivo results indicated that the tumor volumes in mice with cisplatin and clinofibrate combination therapy were significantly reduced (Figure 6B–D). Additionally, the comparable mice weights in the four groups suggested the high safety and low toxicity for combination therapy (Figure 6E). Given that clinofibrate is an inhibitor of cholesterol synthesis, the cholesterol of tumors and serum in mice were detected. The results showed the cholesterol in tumors were reduced in combination therapy and single clinofibrate group (Figure 6F). Moreover, SNORA80B expression was significantly downregulated in both the clinofibrate treatment group and the combination treatment group compared with placebo group (Figure 6G). Immunohistochemistry analysis revealed lower expression of proliferation marker (Ki‐67), AR and cholesterol metabolic proteins (HMGCR, ACAT1 and CYP17A1) in combination treatment than other groups (Figure 6H). Taken together, these data revealed clinofibrate enhanced the sensitivity of cisplatin in ESCC xenograft.

## 3 Discussion

Mounting evidence suggested that the alterations of androgen‐activated AR were critical for the growth, differentiation, and metastasis of many hormone‐related cancers,^[^ ^29^ ^]^ especially in prostate cancer.^[^ ^30^ ^]^ While several studies have revealed the crucial role of AR in the ESCC progression,^[^ ^6^, ^7^ ^]^ noncoding RNAs have been demonstrated as the potential targets of AR in cancers, including miRNAs, lncRNAs and circRNAs.^[^ ^31^ ^]^ Here, we identified SNORA80B, another subtype of noncoding RNAs, as a novel target of AR, was highly expressed in ESCC samples compared to adjacent normal tissues. While snoRNA biogenesis from intronic loci typically involves pre‐mRNA splicing modulated by splicing factors, our study revealed that SNORA80B was aberrantly overexpressed in AR‐activated samples and was transcriptionally regulated by AR, which further promoted ESCC progression. Nevertheless, whether splicing factors participate in SNORA80B biogenesis downstream of AR‐mediated transcription, and how these factors might interact with AR, requires further investigation.

The canonical androgen biosynthetic pathway originates from cholesterol through a series of enzymatic bioconversions, which sustained tumor progression by providing cholesterol for maintenance of membrane structure and oncogenic signaling.^[^ ^32^ ^]^ Additionally, cholesterol dysregulation, including uptake, synthesis, storage and metabolism, has also been linked to the progression of many malignant tumors.^[^ ^9^, ^32^ ^]^ While the precursor role of cholesterol in promoting androgen biosynthesis is well‐characterized, the reciprocal regulation of cholesterol biosynthesis via transcriptional modulation of lipid metabolic genes has been reported but the precise mechanism remains elusive. In this work, we demonstrated that SNORA80B orchestrated cholesterol homeostasis by modulating intracellular cholesterol metabolism, androgen biosynthesis and LDs biogenesis. These findings uncover a novel regulatory nexus from androgen/AR to cholesterol through snoRNA‐mediated mechanism.

In decades, accumulating evidence has proved that snoRNAs are associated with the initiation and development of cancer.^[^ ^33^ ^]^ On the one hand, they function in a canonical manner by regulating the modification of 2′‐O‐methylation or pseudouridylation of RNAs. For example, SNORD11B mediated 2′‐O‐methylated modification on the G509 site of 18S rRNA and accelerated CRC malignancy,^[^ ^34^ ^]^ while SNORA56 promoted the pseudouridylation of 28S rRNA at the U1664 site in CRC.^[^ ^35^ ^]^ Beyond the traditional role in ribosomal biogenesis, emerging studies have revealed snoRNAs regulate tumor progression by modulating oncoproteins’ expression or activating oncogenic pathways. As reported, SNORD50A and SNORD50B snoRNAs directly bind and inhibit K‐Ras in human cancer.^[^ ^36^ ^]^ SNORA28 acted as a molecular decoy that recruited BRD4 to increase the H3K9 acetylation of the promoter in colorectal cancer.^[^ ^37^ ^]^ Meanwhile, targeting SNORA38B attenuated tumorigenesis through GAB2/AKT/mTOR pathway by directly binding to E2F1 in non‐small cell lung cancer.^[^ ^38^ ^]^ In the current study, we found that SNORA80B functioned as an oncogene by binding to YTHDC1 in ESCC. Previous studies have evidence linking YTHDC1 to various facets of RNA expression, such as mRNA stability and mRNA nuclear export.^[^ ^21^ ^]^ Among the m^6^A readers, YTHDC1 is the only member with a localization exclusive to the nucleus. The same subcellular localization of YTHDC1 and SNORA80B provided more binding possibility, which was further validated by RNA pull down and RIP assays in our study.

While our study confirmed the physical interaction between SNORA80B and YTHDC1, we observed a mechanistic dissociation as evidenced by their mutually independent expressions. Our findings indicated that SNORA80B acted as a critical co‐factor that enhanced the reader activity of YTHDC1–specifically, its ability to recognize and bind m^6^A sites–without affecting its expression level. In our study, we discovered conserved m^6^A motifs within the transcripts of critical genes in cholesterol metabolism‐HMGCR (cholesterol synthesis), ACAT1 (cholesterol esterification) and CYP17A1 (steroidogenesis)‐all showing potential YTHDC1 binding domains. In the present study, the results revealed that YTHDC1 recognized and bound to m^6^A‐modified transcripts of cholesterol‐metabolizing enzymes, including HMGCR, ACAT1 and CYP17A1. The enhancement of YTHDC1's reader function by SNORA80B may occur through one or more of the following mechanisms: 1) inducing conformational changes or allosteric activation of YTHDC1 to increase its affinity for m^6^A sites; 2) serving as a molecular scaffold to facilitate the enrichment or stabilization of YTHDC1 at specific sites on m^6^A‐modified transcripts; 3) enhancing the interaction between YTHDC1 and other cofactors to form functional complexes; or 4) potentially modulating post‐translational modifications or subcellular localization of YTHDC1 to optimize its functional activity. However, the precise structural mechanism by which SNORA80B regulates YTHDC1 function requires further investigation (such as through structural biology or protein‐RNA interaction analyses).

Accumulating studies reveal that m^6^A methylation governs the fate of modified RNA, such as stability and nuclear export, which involves in cancer progression and metastasis,^[^ ^39^, ^40^ ^]^ including ESCC.^[^ ^41^ ^]^ In line with the previous studies, our data demonstrated that SNORA80B potentiated mRNA stabilization of cholesterol‐metabolizing enzymes through YTHDC1‐mediated m^6^A recognition. Additionally, cholesterol, as a fundamental structural component of cellular membranes, serves as the biochemical substrate for ACAT1‐catalyzed esterification, driving the biogenesis and functional maturation of LDs. Our data delineated a SNORA80B dependent regulatory axis wherein SNORA80B promoted LDs formation through YTHDC1 mediated mechanism, as evidenced by significant suppression of LDs biogenesis following YTHDC1 knockdown.

Platinum‐based chemotherapy is used as a common therapeutic regiment for patients with advanced ESCC. However, the inevitable emergence of platinum resistance substantially compromises clinical outcomes, underscoring the urgent need for innovative chemosensitization strategies. Although noncoding RNAs play an important role in tumor progression and have great potential as therapeutic targets, several challenges still remain regarding tolerance, specificity and delivery that still hinder their clinical application.^[^ ^42^ ^]^ In the present study, we pioneered an RNA‐targeted drug repurposing approach by screening an FDA‐approved compound library for SNORA80B inhibitor. Intriguingly, clinofibrate, the inhibitor of HMGCR, is a hypelipidemic agent approved by FDA and has been used to reduce serum triglycerides and cholesterol, and found as a potential inhibitor of SNORA80B in this study. Despite the indirect inhibition for SNORA80B by clinofibrate, it synergistically enhanced cisplatin cytotoxicity in ESCC through metabolic‐epigenetic crosstalk. Mechanistically, clinofibrate disrupted the cholesterol‐androgen‐AR positive feedback loop, thereby attenuating SNORA80B‐driven oncogenic programs.

Compared to novel investigational agents, this drug repositioning strategy leverages existing clinical safety profiles, enabling accelerated translation into combination therapy trials. It is worth noting that statins represent another class of drugs that share the same target (HMGCR) and clinical indications as clinofibrate. Currently, seven statin drugs are approved for clinical use worldwide, while mevastatin serves only as a lead compound in research. Although both statins and clinofibrate target HMGCR, their lipid‐lowering profiles differ significantly: statins primarily reduce cholesterol levels, whereas clinofibrate mainly lowers triglyceride concentrations. During our screening, two statins (rosuvastatin and mevastatin) exhibited negligible inhibitory effects on SNORA80B expression compared to the pronounced action of clinofibrate, while other statins even induced a paradoxical elevation. Consequently, clinofibrate was selected for further investigation due to its strongest inhibitory effect on SNORA80B. Despite recent evidence that statins can enhance chemotherapy sensitivity in pancreatic cancer,^[^ ^43^ ^]^ it remains unclear whether statins that showed some inhibitory potential during screening might also mitigate cisplatin resistance in ESCC in a manner comparable to clinofibrate. This issue requires further investigation in future.

**Figure 7:**
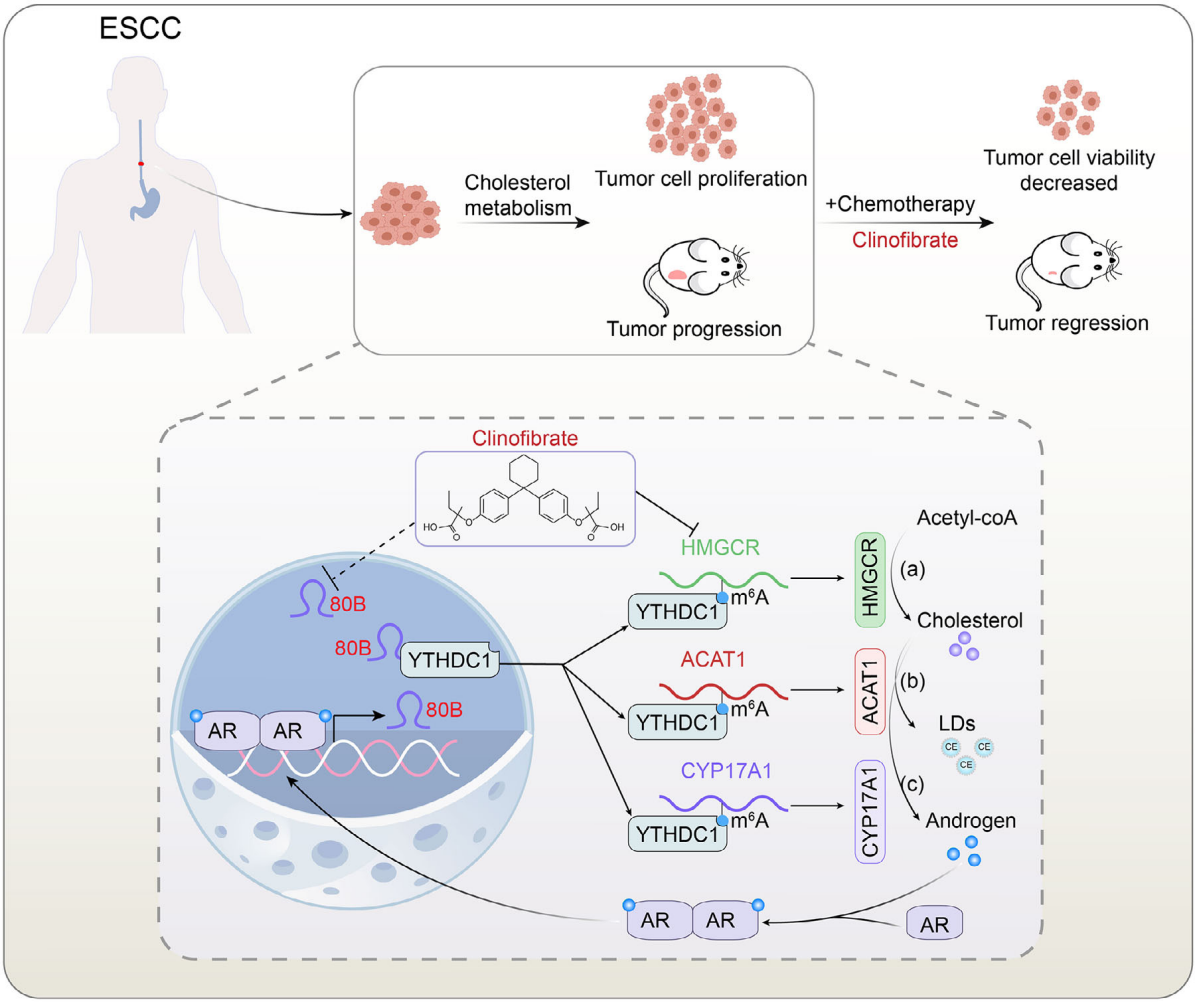
Schematic illustration of SNORA80B function model in ESCC cells with AR activation through YTHDC1‐cholesterol homeostasis axis.

In conclusion, we mechanistically delineated SNORA80B stratified ESCC patients into clinically distinct AR‐high vs AR‐low cohorts. Crucially, we established a snoRNA‐m⁶A regulatory axis wherein SNORA80B interacts with the YTHDC1 reader protein to stabilize the transcripts of cholesterol‐metabolic enzymes thereby fueling ESCC progression. Importantly, our drug repurposing screen identifies clinofibrate, an FDA‐approved HMGCR inhibitor, as a novel metabolic‐epigenetic modulator that disrupts the SNORA80B/YTHDC1/cholesterol homeostasis cascade through cholesterol synthesis suppression and AR signaling attenuation, ultimately achieving synergistic sensitization of cisplatin‐resistant ESCC (**Figure** **7**). These findings contribute to the advancement of ESCC research by expanding our comprehension of snoRNA‐mediated epigenetic‐metabolic interplay in cancer, positioning SNORA80B not only as a prognostic biomarker for precision oncology but also as a therapeutic vulnerability targetable through metabolic rewiring strategies. The clinical translatability of combining cholesterol‐lowering agents with platinum chemotherapy opens new avenues in ESCC.

## 4 Experimental Section

### Cell Culture and Reagents

Human ESCC cell lines in this study were kindly provided by Professor Yutaka Shimada from Kyoto University, Japan. KYSE150 (RRID: CVCL_1348) cell line was cultured in RPMI‐1640/Ham's F12 plus 2% fetal bovine serum (FBS). Other ESCC cell lines (YES2/RRID: CVCL_E322, KYSE30/RRID: CVCL_1351, KYSE70/RRID: CVCL_1356, KYSE140/RRID: CVCL_1347, KYSE150/RRID: CVCL_1348, KYSE180/RRID: CVCL_1349, KYSE410/RRID: CVCL_1352, KYSE450/RRID: CVCL_1353, KYSE510/RRID: CVCL_1354, COLO680N/RRID: CVCL_1131) were maintained in RPMI‐1640 with 10% FBS. HEK‐293T was cultured in DMEM complemented with 10% FBS. All mediums were supplemented with 1% penicillin/streptomycin. All cell lines were maintained at 37 °C in 5% CO_2_. All cell lines were authenticated by short tandem repeat (STR) profiling.

The antibodies and siRNA used in this study were listed in Tables S2 and S3 (Supporting Information).

### RNA Extraction and Library Preparation

Total RNA was extracted from ESCC tissues using the TRIzol reagent (Invitrogen, USA). RNA integrity and concentration were assessed using an Agilent 2100 Bioanalyzer (Agilent Technologies, USA) and a Qubit fluorometer (Thermo Fisher Scientific, USA), respectively. To simultaneously profile both coding and non‐coding transcripts, ribosomal RNA (rRNA) was depleted from total RNA using the Ribo‐Zero rRNA Removal Kit (Epicentre, USA). The rRNA‐depleted RNA was then used for library preparation. The sequencing libraries were constructed using the NEBNext Ultra II Directional RNA Library Prep Kit for Illumina (New England Biolabs, USA) according to the manufacturer's instructions. Then the libraries were size‐selected using agarose gel electrophoresis to enrich for fragments longer than 100 nucleotides, aiming to capture long non‐coding RNAs (lncRNAs) and messenger RNAs (mRNAs). Finally, the qualified libraries were PCR‐amplified and paired‐end 150 bp sequencing was performed on an Illumina NovaSeq 6000 platform (Illumina, USA).

### RNA Sequencing Data Analysis

We processed the raw sequencing data with fastp (v0.23.2) for quality control and adapter trimming under default parameters. Cleaned paired‐end reads were then aligned to the GRCh38 reference genome with HISAT2 (v2.2.1), and gene expression was quantified from the alignments using featureCounts (v2.0.3) to generate raw count matrices. To analyze snoRNA expression, raw counts corresponding to snoRNA features (as annotated in the GENCODE GRCh38 GTF file with gene_type = “snoRNA”) were extracted from the full count matrix for subsequent analysis. Subsequent differential expression analysis was performed in R (v4.4.2) using the edgeR package (v4.4.1). We filtered low‐abundance genes, retaining only those with a CPM > 1 in at least three samples. A generalized linear model incorporating patient ID as a covariate was applied to identify differentially expressed genes. Significant differential expression was defined by log2 fold change (|log2FC|) > 0.5 and false discovery rate (FDR)‐adjusted *p*‐value < 0.05.

### Functional Enrichment Analysis

To investigate the functions of differentially expressed genes (DEGs) identified across both ESCC patient tissues (normal vs tumor) and cellular groups (si‐YTHDC1 vs control), we performed gene set enrichment analysis (GSEA) using the enricher function in the clusterProfiler package (v4.14.0). Specifically, we retrieved gene sets related to glycolysis, fatty acid metabolism, amino acid metabolism, and nucleotide metabolism from the MSigDB database (v7.5.1). Significantly enriched terms were defined as those with an adjusted *p*‐value < 0.05.

### Pathway Activity Assessment

We employed the Gene Set Variation Analysis (GSVA) algorithm (v2.0.4) to quantify the activity levels of key pathways across individual samples. Specific gene sets representing pathways of interest (REACTOME: ANDROGEN BIOSYNTHESIS, GOBP: ANDROGEN METABOLIC PROCESS and REGULATION OF CHOLESTEROL METABOLIC PROCESS) were obtained from the MSigDB database (v7.5.1).

### RNA Extraction and RT‐qPCR

Total RNA was extracted from ESCC cells and tissue by RNAExpress Total RNA Kit (NCM Biotech, China). Reverse transcription was performed by Evo M‐MLV RT Mix Kit with gDNA Clean (Accurate Biotechnology, China). RT‐qPCR was conducted by SYBR Green Premix Pro Taq HS qPCR Kit (Accurate Biotechnology, China).

For the detection of SNORA80B in tumor xenografts, RNA was extracted from FFPE tissues using RNAprep Pure FFPE Kit (TIANGEN, China) following the manufacturer's instructions.

The sequences of primers were listed in Table S4 (Supporting Information).

The clinical characteristics of patients in Cohort 1 and Cohort 2 from whom ESCC tissues were obtained for SNORA80B expression analysis were summarized in Table S5 (Supporting Information).

### Transwell Assay

For cell migration, the indicated cells were suspended in serum‐free medium and further seeded in upper chamber of transwell. For cell invasion, the inside of transwell was covered with 2% Matrigel (Corning, USA) for 6 h at 37 °C. Then the suspension of indicated cells in serum‐free medium was seeded into the upper chamber of transwell. After 24 h, the transwells were fixed with methanol and stained with crystal violet. The cell intensity of migrated or invaded cells was counted by Image J software.

### Chromatin Immunoprecipitation (ChIP) Assay

The ChIP assay was performed using a Pierce Magnetic ChIP kit (ThermoFisher, USA) following the manufacturer's instructions. In brief, the sonicated and sheared chromatin was immunoprecipitated by AR antibody or negative IgG antibody at 4 °C overnight. Then the proteins were removed from the mixture and DNA samples were quantified by RT‐qPCR.

### Dual‐Luciferase Reporter Assay

The detection of luciferase activity was conducted by a luciferase assay system kit (Promega Corp., USA) according to the manufacturer's protocol. In short, cells were transiently co‐transfected with siRNA targeting AR and SNORA80B promoter cloned into pGL3 vector. After 24 h, cells were lysed and luciferase activity was measured using the designated kit. Firefly luciferase activity was normalized to that of renilla fluorescein.

### Generation of Stable Cell Lines

The sequence of SNORA80B was cloned into the pHBLV‐CMV‐MCS‐fLUC‐PURO lentiviral vector. To establish stable cell lines expressing Lv‐Ctrl or Lv‐80B, cells infected with Lv‐Ctrl or Lv‐80B lentivirus in medium containing 5 µg mL^−1^ polybrene, followed by selection with 2 µg mL^−1^ puromycin to generate stable cell lines. The overexpression efficiency of SNORA80B was determined by qPCR.

### RNA Pull Down Assay and Mass Spectrometry

The sequences for sense and antisense of SNORA80B were cloned into pcDNA3.1 plasmid vector. The sense or antisense of biotin‐label SNORA80B was transcribed in vitro using biotin‐16 UTP by MegaScript T7 Transcription Kit (ThermoFisher) according to the manufacturer's instructions, respectively. 2 µg RNA was suspended in 25 µL structure buffer (10 mm Tris HCl pH7.0, 0.1 m KCl, 10 mm MgCl_2_) and denatured at 95 °C for 2 min, in the ice for 3 min, and recovered at room temperature (RT) for 30 min. Then the biotinylated RNA and cell lysates were incubated in binding buffer (25 mm Tris HCl pH7.4, 150 mm KCl, 0.5% NP40, 0.5 mm DTT) at RT for 30 min. Next, 30 µL Streptavidin beads was added to the mixture above and incubated at RT for 20 min. Then the beads were washed for five times by washing buffer (25 mm Tris HCl pH7.4, 150 mm KCl, 0.5% NP40, 50 mm NaCl). The proteins binding with beads were washed and denatured by loading buffer, and further detected by mass spectrometry or Western blot. For the validation of the physical interaction between YTHDC1 and SNORA80B, purified recombinant YTHDC1 protein was used. The experimental procedure followed a similar protocol as described previously, with the exception that cell lysates were replaced with wash buffer. For the binding assays between SNORA80B and truncated mutants of YTHDC1, cell lysates transfected with different truncated plasmids of YTHDC1 were used in RNA pull down experiment.

Mass spectrometry measurements were performed as previously described.^[^ ^44^ ^]^

### RNA Immunoprecipitation (RIP) Assay

The cells were crosslinked by 1% formaldehyde at RT for 10 min and terminated by 140 mm glycine buffer. Then the cells were washed by pre‐cooled PBS and lysed in lysis buffer (25 mm Tris‐HCl pH7.4, 150 mm NaCl, 1% NP‐40, 1 mm EDTA, 5% glycerol, Protease inhibitor and RNase inhibitor). The mixture of protein A/G beads and indicated antibody were suspended in binding buffer (20 mm Tris‐HCl pH 7.4, 50 mm NaCl, 2 mm MgCl_2,_ 5% glycerol, 0.1% Tween‐20, Protease inhibitor and RNase inhibitor) and incubated at RT for 2 h. Next, the cell lysates were added to the above mixture and incubated overnight at 4 °C. The beads were washed by washing buffer (20 mm Tris‐HCl pH 7.4, 10 mm NaCl, 0.1% Tween‐20, 50 mm NaCl) and further detected by RT‐qPCR and Western blot.

### Me‐RIP (m^6^A‐RNA Immunoprecipitation) Assay

The MeRIP assay was performed according to the manufacturer's instructions of riboMeRIP m^6^A Transcriptome Profiling Kit (Ribo Biotechnology, Guangzhou, Guangdong, China). Briefly, total RNA was extracted and captured by m^6^A‐specific antibody or IgG and affinity beads, and the mixture was incubated at room temperature for 90 min. Then the RNA fragments bound to the affinity beads were extracted with elution buffer. RT‐qPCR was performed to analyze the m^6^A‐enriched content of the target RNA. The specific primers for m^6^A methylation sites of HMGCR, ACAT1 and CYP17A1 were listed in Table S4 (Supporting Information).

### BODIPY 493/503 Staining

The lipophilic fluorescence dye BODIPY 493/503 was used to monitor the content of neutral lipids in ESCC cells. The indicated ESCC cells were inoculated into plates for confocal and stained with BODIPY 493/503 working solution in the dark for 30 min at 37 °C, then stopped by washing the cells with PBS. Representative images were taken with confocal laser scanning microscope.

### Untargeted Metabolomic Profiling

KYSE150 cells transfected with either pcDNA‐80B or an empty vector, along with si‐YTHDC1 or NC, were washed twice with ice‐cold PBS and harvested by trypsinization. The cell pellets were then washed three times with ice‐cold PBS, flash‐frozen in liquid nitrogen for 1 min, and stored for subsequent analysis. All detections and analyses were performed by Biotree Biomedical Technology Co., Ltd (Shanghai, China).

### High Throughput Screening for SNORA80B Inhibitor

This assay was performed as previously described.^[^ ^45^ ^]^ Briefly, KYSE450 cells were seeded to achieve 50% confluence. Following 24 h adherence, individual FDA‐approved drugs from the screening library (Selleckchem, Cat. #L1300) were administered to cells for 48 h. Total RNA was extracted and further converted to cDNA. The expression of SNORA80B was measured by qPCR and normalized by U6. The fold change of SNORA80B expression was calculated compared to parental cells.

### 50% Inhibitory Concentration (IC50)

ESCC cells transfected indicated plasmids or ASO were seeded into 96‐well plates, and divided into experimental and negative control groups. Cells were treated with constant clinofibrate and a range of cisplatin concentrations or constant cisplatin and a range of clinofibrate. After 72 h of treatment, Cell Counting Kit‐8 (CCK8) (NCM Biotech, China) was used to assess cell viability and performed according to the manufacturer's instruction.

### Animal Studies

Animal experiments were conducted in accordance with the guidelines for the care of laboratory animals. All animals were purchased from Beijing HFK Bioscience Company (HFK Bioscience, China). Six‐week‐old male nude BALB/c mice were used to establish the xenograft tumor model.

For lung metastasis model, the indicated tumor cells (5 × 10^5^) were injected via tail vain of BalB/c nude mice to assess the role of SNORA80B in tumor cell metastasis. The metastatic progression was monitored via live animal imaging system. The pulmonary metastatic nodules were stained with hematoxylin and eosin (H&E).

For clinofibrate or cisplatin treatment, 2 × 10^6^ KYSE450 cells were implanted into the right underarm of the mice. When the tumor volumes were up to 100 mm^3^, the mice were divided into four groups randomly, including placebo, clinofibrate treatment, cisplatin treatment and combination treatment. Clinofibrate was given by oral administration (gavage) at the dosage of 100 mg kg^−1^ every day, and cisplatin was given by intraperitoneal injection at the dosage of 2 mg kg^−1^ once a week. The subcutaneous tumor size and mice weights were measured and calculated at the indicated time points. The xenograft tumors were removed for mass determination and immunohistochemistry. The volume was estimated using the formula 0.5 × length × width.^2^

### Statistical Analysis

Each experiment was performed in triplicate, and all data were shown as the mean ± standard deviation. Student's *t*‐test was used for two groups analysis. One‐way ANOVA and two‐way ANOVA were used for multiple groups analysis. Kaplan–Meier analysis with log‐rank test was used for survival analysis. *p* < 0.05 was considered to be statistically significant. All statistical analyses were performed with GraphPad Prism 8 (GraphPad Software Inc.).

## Conflict of Interest

The authors declare no competing interests.

## Author Contributions

H.Y., G.G., and L.L. contributed equally to this work as co‐first authors. Y.S., Z.Z., and H.Y. conceived and designed the experiments. H.Y., Z.Z., and G.G. performed the experiments. H.Y., Z.Z., G.G., and L.L. performed the statistical collection and analysis. S.H., M.T., and Y.N. performed mass spectrometry. H.Y. and Z.Z. wrote the manuscript and Y.S. revised the manuscript. All authors read and approved the final manuscript.

## Supporting information



Supporting Information

Supporting Information

Supporting Information

Supporting Information

## Data Availability

The data that support the findings of this study are available from the corresponding author upon reasonable request.
