# Supplementary material for: Clinofibrate Disrupts the SNORA80B/YTHDC1‐Driven M6A Modification to Suppress Cholesterol Metabolism and Cisplatin Resistance in ESCC
Source: Adv Sci (Weinh). 2025 Nov 3;13(3):e09574. doi: 10.1002/advs.202509574 (PMC12884767; doi:10.1002/advs.202509574)
Supplement: Supplementary file 1 — Supporting Information [file ADVS-13-e09574-s004.docx]

**Supplementary materials**

**Clinofibrate Disrupts the SNORA80B/YTHDC1-driven m^6^A Modification to Suppress Cholesterol Metabolism and Cisplatin Resistance in ESCC**

Hongyu Yuan^a, b, †^, Ge Ge^a, †^, LiQiu Liu^a, †^, Sijun Hu^c^, Miaomiao Tian^c^, Yongzhan Nie^c^, Zitong Zhao^a, *^, Yongmei Song^a, *^

^a^ State Key Laboratory of Molecular Oncology, National Cancer Center/National Clinical Research Center for Cancer/Cancer Hospital, Chinese Academy of Medical Sciences and Peking Union Medical College, 100021, Beijing, China.

^b^ State Key Laboratory of Bioactive Substance and Function of Natural Medicines, Institute of Materia Medica, Chinese Academy of Medical Sciences and Peking Union Medical College, Beijing 100050, China

^c^ State Key Laboratory of Holistic Integrative Management of Gastrointestinal Cancers, Xijing Hospital of Digestive Diseases, Fourth Military Medical University, Xi'an 710032, China

^†^ These authors contributed equally.

*Correspondence: Yongmei Song, State Key Laboratory of Molecular Oncology, National Cancer Center/National Clinical Research Center for Cancer /Cancer Hospital, Chinese Academy of Medical Sciences and Peking Union Medical College, Beijing, China. Tel: 86-010-8778-8422, Fax: 86-10-6771-5058, Email: symlh2006@163.com, [songym@cicams.ac.cn](mailto:songym@cicams.ac.cn). Zitong Zhao, State Key Laboratory of Molecular Oncology, National Cancer Center/National Clinical Research Center for Cancer /Cancer Hospital, Chinese Academy of Medical Sciences and Peking Union Medical College, Beijing, China. E-mail: zhaozitong880807@126.com.

**Supplementary methods**

**Cell proliferation assay**

The cell proliferation was detected by xCELLigence Real-Time Cell Analyzer (RTCA)-MP system as described previously. ^[1]^ Briefly, the indicated cells were seeded in E-Plate 96. Then the signal of cell proliferation was recorded automatically every 15min by RTCA-MP system.

**Colony formation assay**

The indicated cells were seeded into 6-well plate and cultured for 10 days. Then the cells were fixed with methanol and stained with crystal violet. The ability to form colonies was measured by G:box (Syngene).

**Sub-cellular fractionation assay**

The nucleoplasmic separation kit (Beyotime) was used for sub-cellular fractionation assay according to the manufacturer’s instructions. All primer sequences in this study are listed in supplementary table 4.

**RNA stability assay**

The transfected cells were treated with actinomycin D for the indicated times. Total RNA was extracted and further subjected to RT-qPCR. The change of expression of CYP17A1 mRNA was observed and shown by GraphPad Prism 8 software.

**Immunohistochemistry assay**

Tissues were fixed in 4% formaldehyde and embedded in paraffin. For histopathological visualization, 4 μm tissue slides were deparaffinized and rehydrated, followed by antigen retrieval. Then, endogenous peroxidase activity was blocked by endogenous peroxidase blocking buffer (ZsbBio, China) for 20 min and incubated in goat serum (ZsbBio, China) for 20 min. Slides were incubated with primary antibodies at 4℃ overnight. Next, tissues were incubated with secondary antibody for 30 min at RT. Finally, slides were incubated with DAB working solution and stained by hematoxylin for nuclear, dehydrated and mounted with a cover slip using mounting medium.

**References**

1. Song, Y., L. Li, Y. Ou, et al., *Identification of genomic alterations in oesophageal squamous cell cancer*. Nature, 2014. **509**(7498): p. 91-5.

**Supplementary Figure legends**

**
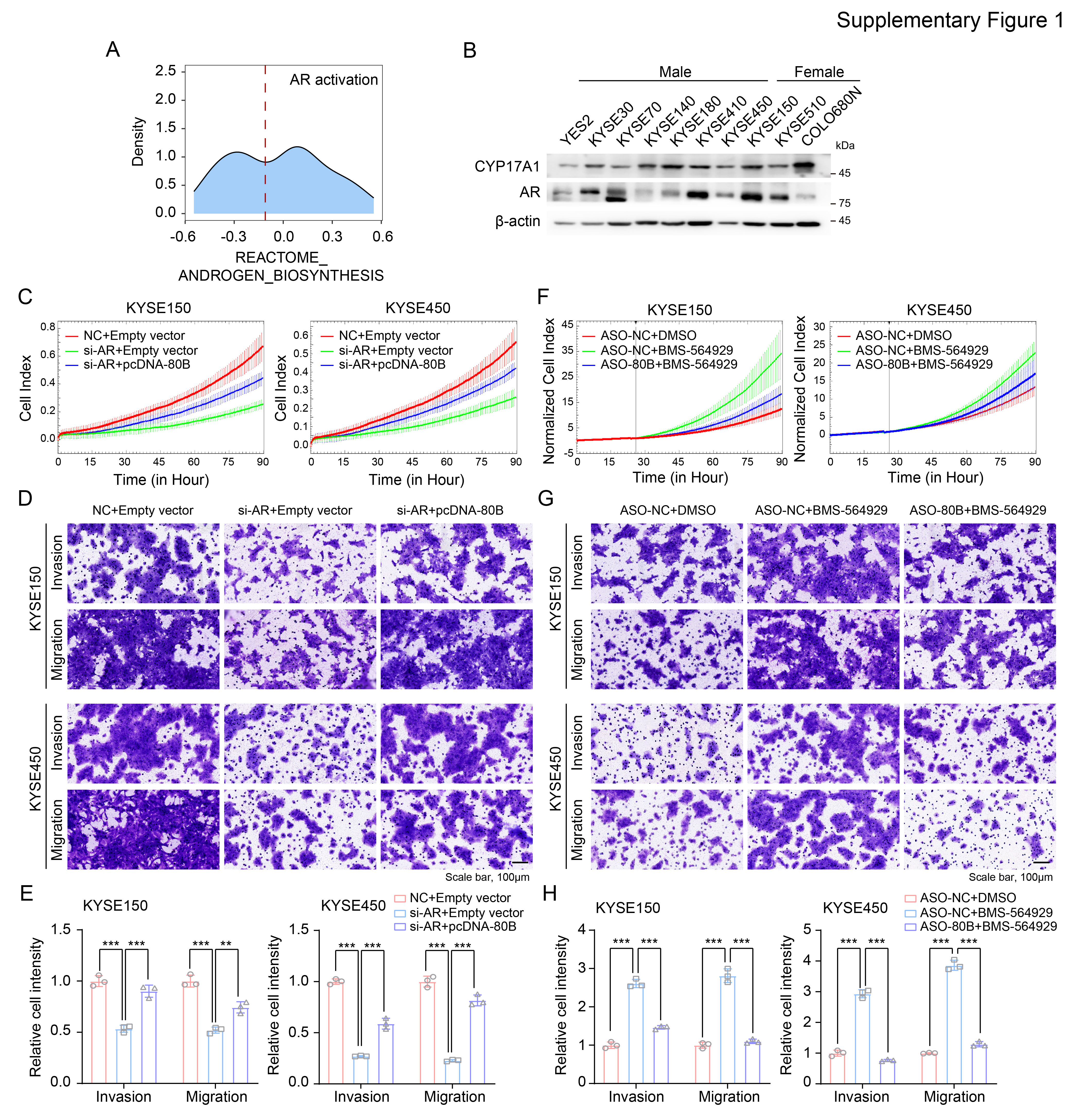
Supplementary Fig. 1** The expression of SNORA80B and AR in ESCC cells. (**A**) The androgen biosynthesis pathway density plot for ESCC samples, with the right side of the dashed line indicating AR activation. (**B**) The protein levels of AR and CYP17A1 in ESCC cells. (**C**) The cell proliferation ability detected by RTCA assays in ESCC cells with co-transfection of si-AR and pcDNA-80B or empty vector. (**D and E**) Images of transwell assays (D) and quantitative analysis (E) in ESCC cells with co-transfection of si-AR and pcDNA-80B or empty vector. (**F**) The cell proliferation ability of ESCC cells following transfection with ASO-80B and treatment with BMS-564929 or DMSO. (**G and H**) Images of transwell assays (G) and quantitative analysis (H) in ESCC cells following transfection with ASO-80B and treatment with BMS-564929 or DMSO. The data are presented as the means ± SEM (n ≥ 3). One-way ANOVA was performed to compare multiple groups. *p < 0.05, **p < 0.01, ***p < 0.001.

**
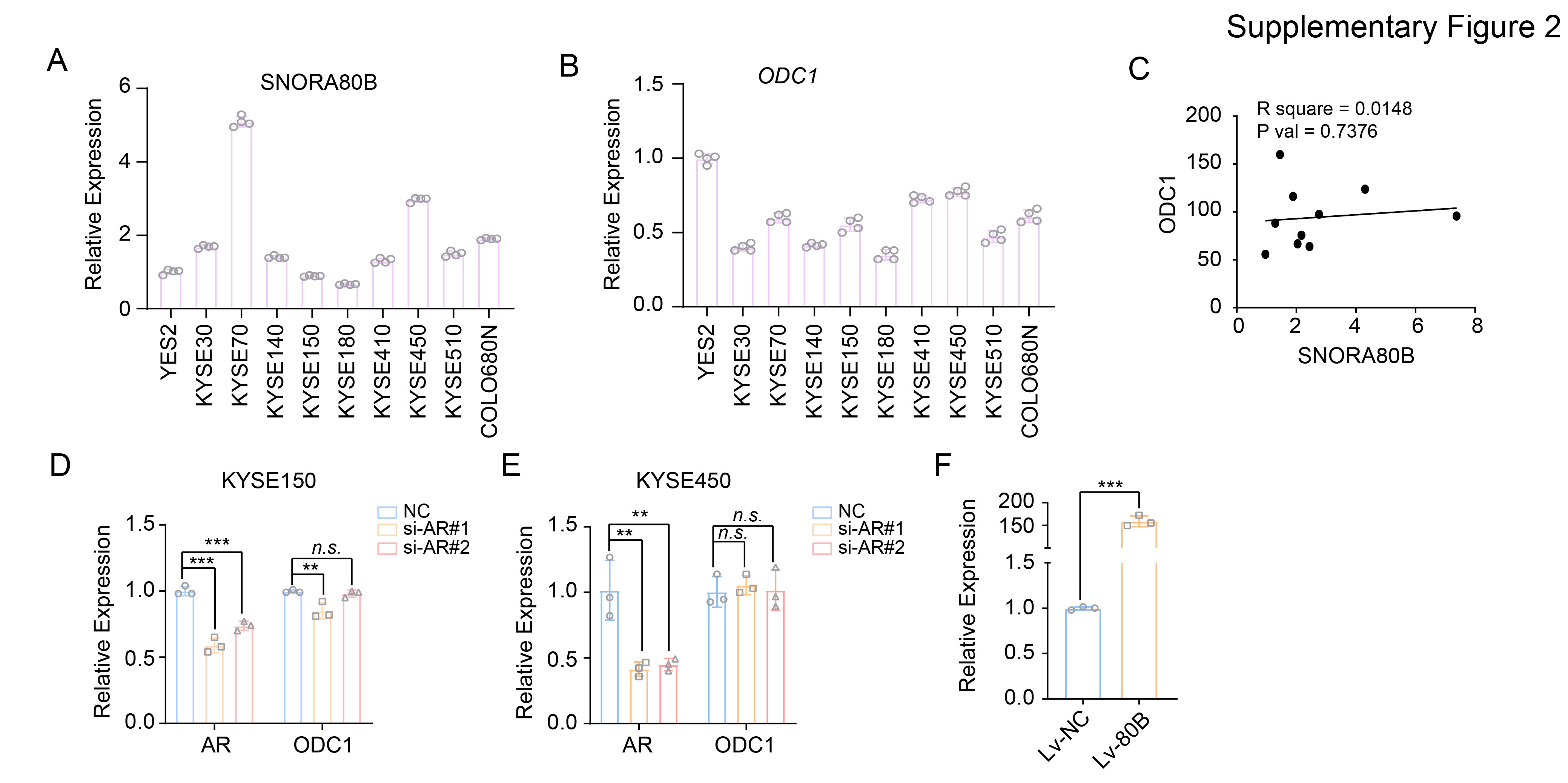
Supplementary Fig. 2** The independence of SNORA80B expression from ODC1. (**A**) The expression of SNORA80B in ESCC cells. (**B**) The mRNA expression of *ODC1*, the host gene of SNORA80B, in ESCC cells. (**C**) The correlation between SNORA80B and ODC1 in ESCC cells. (**D and E**) The expression of ODC1 in KYSE150 (D) and KYSE450 (E) cells transfected with AR siRNA. (**F**) The expression of SNORA80B in nodules of lung metastasis tissues from SNORA80B overexpression xenografts. The data are presented as the means ± SEM (n ≥ 3). C: Correlation analyses were conducted using Pearson’s correlation coefficient. A two-tailed Student’s t-test was performed to compare the two groups. *p < 0.05, **p < 0.01, ***p < 0.001. *n.s.* means not significant.

**
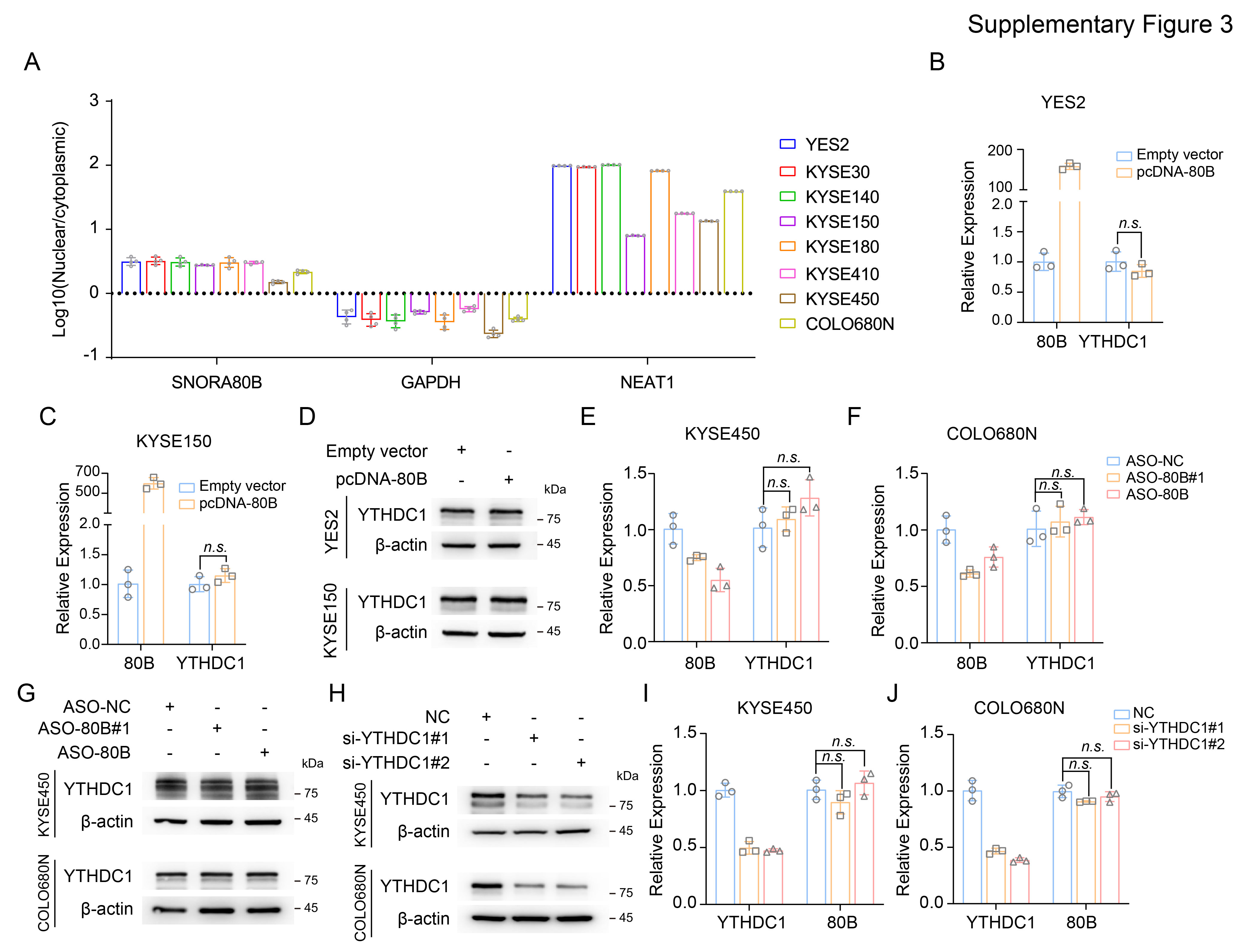
Supplementary Fig. 3** The regulation between SNORA80B and YTHDC1. (**A**) The nuclear/cytoplasmic ratio of SNORA80B in sub-cellular fractions in ESCC cells. GAPDH and lncRNA-NEAT1 are used as cytoplasmic and nuclear markers, respectively. (**B and C**) The mRNA expression of YTHDC1 in ESCC cells with SNORA80B overexpression. (**D**) Western blot analysis indicating the effects of SNORA80B overexpression on the protein level of YTHDC1. (**E and F**) qPCR analysis showing effects on the mRNA level of YTHDC1 in SNORA80B knockdown cells. (**G**) The protein level of YTHDC1 in SNORA80B knockdown cells. (**H**) The knockdown efficiency of YTHDC1 by siRNA in ESCC cells. (**I and J**) The effects on SNORA80B expression in YTHDC1 knockdown cells. The data are presented as the means ± SEM (n ≥ 3). A two-tailed Student’s t-test was performed to compare the two groups. *n.s.* means not significant.

**
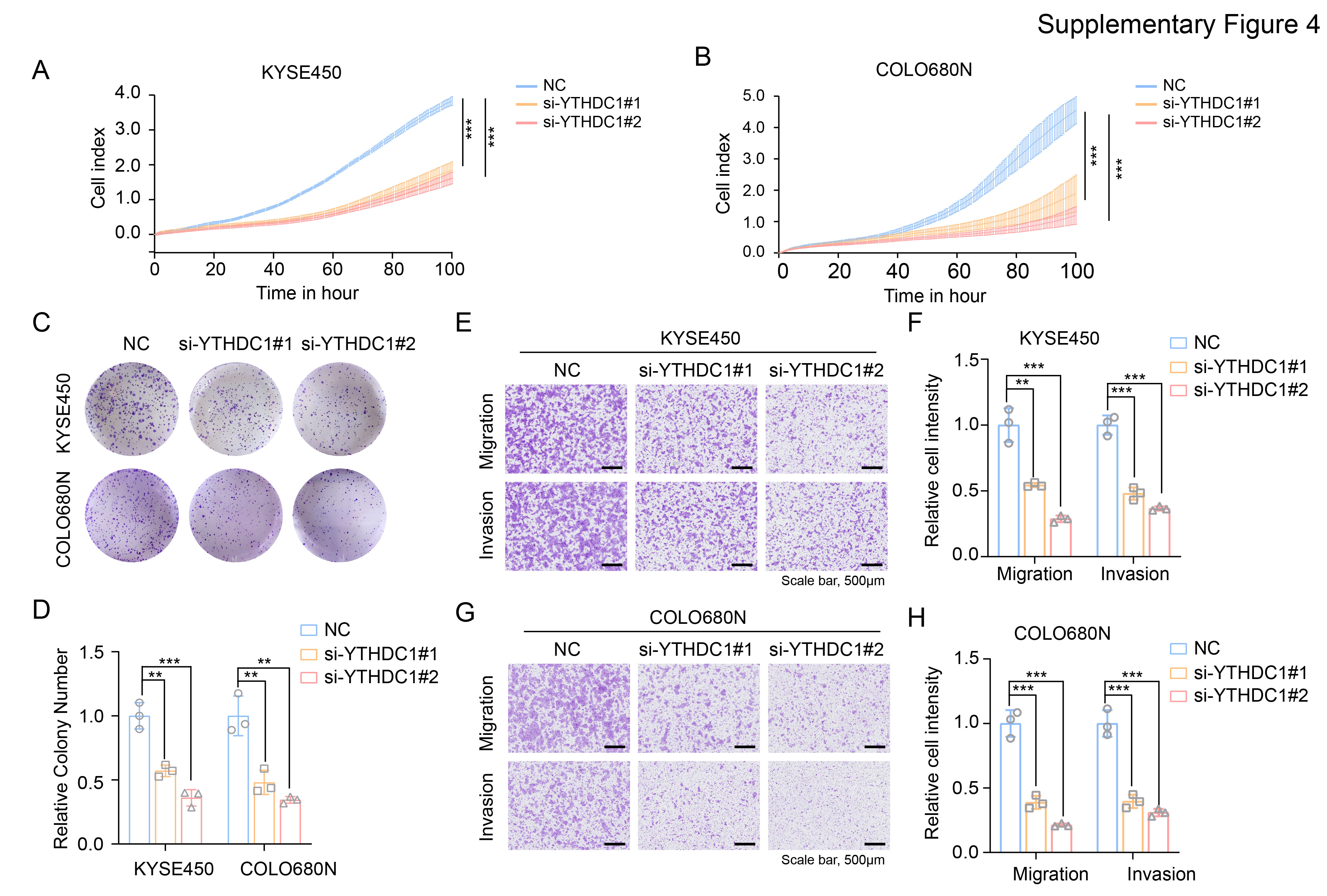
Supplementary Fig. 4** The function of YTHDC1 on ESCC cells. The malignant phenotype is detecting in ESCC cells with YTHDC1 knockdown in vitro, including (**A and B**) RTCA assays, (**C and D**) colony formation assays, (**E-H**) transwell assays. The data are presented as the means ± SEM (n ≥ 3). A two-tailed Student’s t-test was performed to compare the two groups. *p < 0.05, **p < 0.01, ***p < 0.001.

**
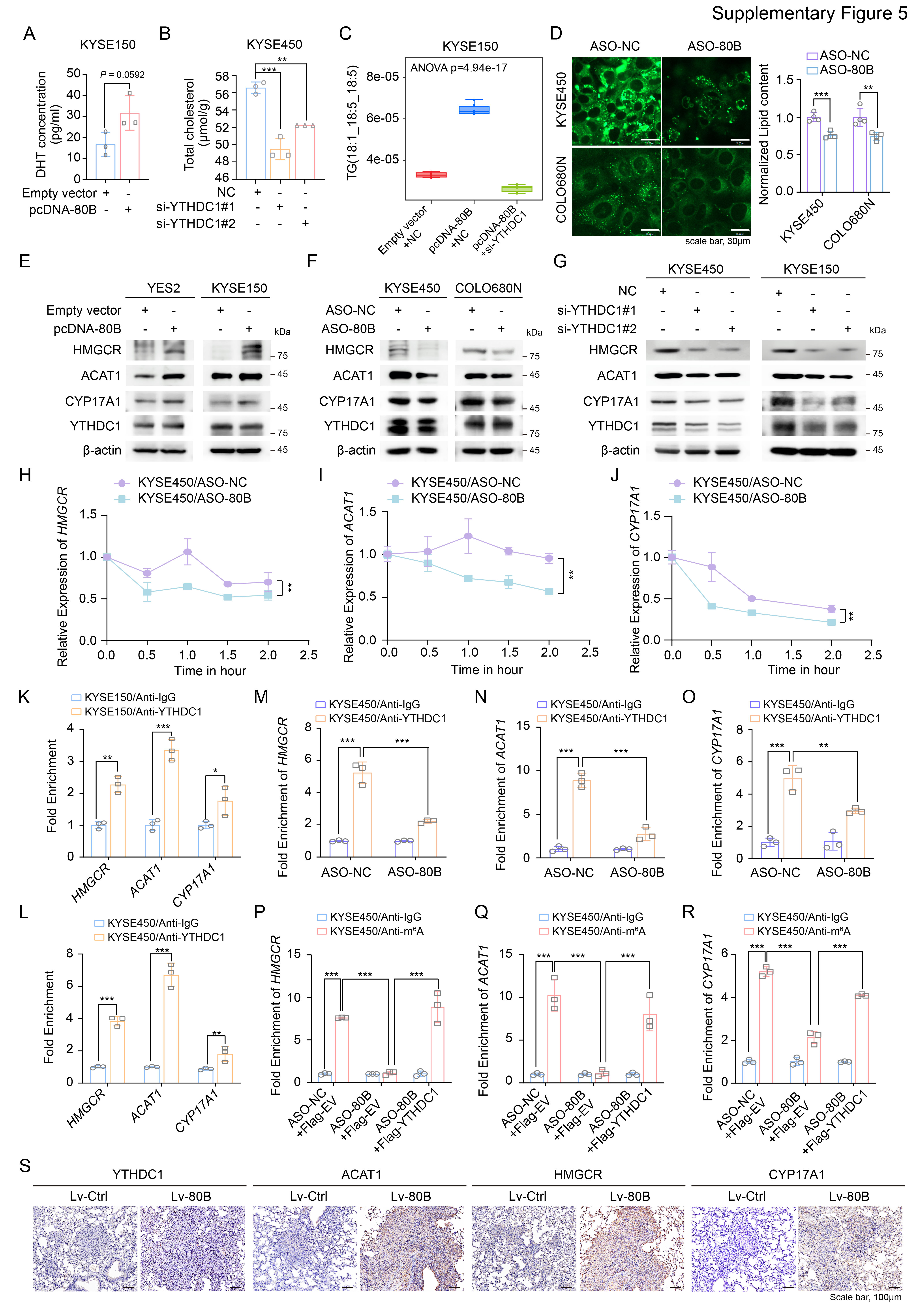
Supplementary Fig. 5** The SNORA80B/YTHDC1 axis in regulating cholesterol homeostasis. (**A**) The detection of DHT levels in SNORA80B upregulation compared with negative control cells. (**B**) Total cholesterol contents in silencing YTHDC1 compared with negative control cells. (**C**) The boxplot analysis of triacylglycerol (TG) in the indicated cells. (**D**) Representative confocal images and quantification of BODIPY in ESCC cells with ASO-SNORA80B vs. ASO-NC cells. (**E**) The protein levels of HMGCR, ACAT1 and CYP17A1 indicating the effects of SNORA80B overexpression in ESCC cells. (**F**) The protein expression of HMGCR, ACAT1 and CYP17A1 in ESCC cells with SNORA80B knockdown. (**G**) Western blot of HMGCR, CYP17A1 and ACAT1 expression in ESCC cells transfected with siRNA of YTHDC1. (**H-J**) The mRNA stability of HMGCR (H), ACAT1 (I) and CYP17A1 (J) by actinomycin D inhibition in KYSE450 cells with SNORA80B knockdown. (**K-L**) RIP-qPCR analysis the binding ability of YTHDC1 with HMGCR, ACAT1, CYP17A1 in ESCC cells by YTHDC1 antibody. IgG was used as a negative control. (**M-O**) RIP-qPCR analysis the binding ability of YTHDC1 with HMGCR, ACAT1, CYP17A1 in KYSE450 cell line with SNORA80B knockdown by YTHDC1 antibody. IgG was used as a negative control. (**P-R**) m^6^A methylation levels of HMGCR, ACAT1, CYP17A1 in KYSE450 cell line transfected with ASO-NC or ASO-80B and either Flag-NC or Flag-YTHDC1 by meRIP assays. (**S**) The IHC staining for YTHDC1, HMGCR, ACAT1 and CYP17A1 in the lung metastases of xenograft tumors. The data are presented as the means ± SEM (n ≥ 3). A two-tailed Student’s t-test was performed to compare the two groups. One-way ANOVA and two-way ANOVA were used for multiple groups. *p < 0.05, **p < 0.01, ***p < 0.001.

**
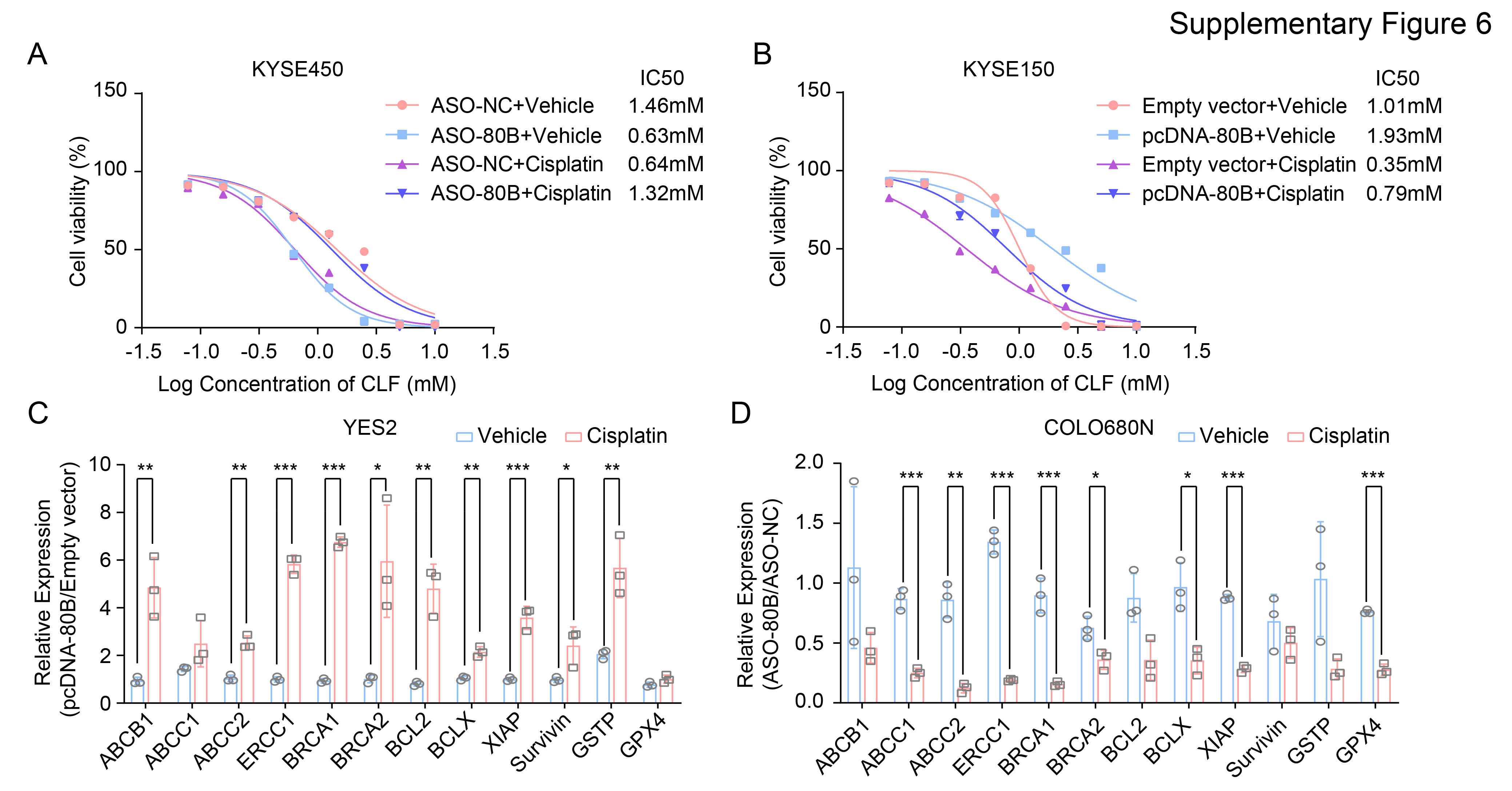
Supplementary Fig. 6** The inhibitor for SNORA80B enhanced the sensitivity of ESCC cells to cisplatin. (**A**) The percent viability (CCK-8 assay) of KYSE450 cells transfected with ASO-80B after 72h treatment with indicated doses of CLF and constant treatment with cisplatin (5µM) or control DMSO. (**B**) The percent viability (CCK-8 assay) of KYSE150 cells transfected with pcDNA-80B after 72h treatment with indicated doses of CLF and constant treatment with cisplatin (10µM) or control DMSO. (**C**) The expressions of cisplatin-resistance related genes in YES2 cells transfected with pcDNA-80B or empty vector, following treatment with cisplatin and vehicle. (**D**) The expressions of cisplatin-resistance related genes in COLO680N cells transfected with ASOA-80B or ASO-NC, following treatment with cisplatin and vehicle. The data are presented as the means ± SEM (n ≥ 3). A two-tailed Student’s t-test was performed to compare the two groups. *p < 0.05, **p < 0.01, ***p < 0.001.

**Supplementary table 1. The top 50 compounds with high inhibition of SNORA80B.**

| **Compound** | **Target** | **FoldChange** | **Log (FoldChange)** |
| --- | --- | --- | --- |
| Fluocinonide | Glucocorticoid Receptor | 0.035 | -1.455750399 |
| Clotrimazole | CYP | 0.055 | -1.256800232 |
| Chloroxine | Anti-infection | 0.060 | -1.219171482 |
| Rifapentine | DNA/RNA Synthesis | 0.062 | -1.210140583 |
| Enasidenib (AG-221) | Dehydrogenase | 0.063 | -1.204119983 |
| Curcumin | NFkB，HDAC，Nrf2 | 0.063 | -1.198099383 |
| Prazosin HCl | Adrenergic Receptor | 0.066 | -1.180250365 |
| Vardenafil HCl Trihydrate | PDE | 0.071 | -1.149217914 |
| Metoclopramide HCl | Dopamine Receptor | 0.079 | -1.101890565 |
| Metaproterenol Sulfate | β2-adrenergic receptor | 0.082 | -1.087209898 |
| Ledipasvir (GS5885) | HCV Protease | 0.084 | -1.074173589 |
| Tacrine HCl | AChR | 0.087 | -1.060252944 |
| Enoxacin | Topoisomerase | 0.089 | -1.052099835 |
| Vorapaxar | PAR1 | 0.089 | -1.048524546 |
| Prostaglandin E2 (PGE2) | others | 0.090 | -1.047584385 |
| Brimonidine Tartrate | Adrenergic Receptor | 0.090 | -1.044650292 |
| Oxiracetam | GABA Receptor | 0.091 | -1.039165607 |
| Pilocarpine HCl | AChR | 0.092 | -1.034474373 |
| Carvedilol | Adrenergic Receptor | 0.092 | -1.034038035 |
| Fidaxomicin | DNA/RNA Synthesis | 0.096 | -1.019492696 |
| Pasiniazid | others | 0.101 | -0.997272359 |
| Nystatin (Fungicidin) | Anti-infection | 0.102 | -0.993398986 |
| Sulfalozine sodium | Anti-infection | 0.103 | -0.985873236 |
| Sulfadiazine | Anti-infection | 0.104 | -0.981357786 |
| Azacitidine | DNA Methyltransferase | 0.107 | -0.972326886 |
| Buflomedil HCl | Adrenergic Receptor | 0.108 | -0.968223543 |
| Nilotinib hydrochloride | Bcr-Abl | 0.110 | -0.95882102 |
| Meticrane | Sodium Channel | 0.110 | -0.95840893 |
| Clinofibrate | HMG-CoA Reductase | 0.111 | -0.955366369 |
| Nateglinide | Potassium Channel | 0.112 | -0.949850372 |
| Azaperone | Dopamine Receptor | 0.114 | -0.944379089 |
| Miconazole Nitrate | Anti-infection | 0.115 | -0.940718736 |
| Isosorbide | others | 0.117 | -0.93246098 |
| Mexiletine HCl | Sodium Channel | 0.118 | -0.927396477 |
| Tyloxapol | lipoprotein lipase | 0.118 | -0.92739352 |
| Tranylcypromine (2-PCPA) HCl | MAO | 0.119 | -0.9235644 |
| Mepenzolate Bromide | mAChR | 0.119 | -0.923425738 |
| Netilmicin Sulfate | Anti-infection | 0.120 | -0.922529404 |
| Niraparib (MK-4827) tosylate | PARP | 0.120 | -0.919377286 |
| Sulfamethoxypyridazine | Anti-infection | 0.122 | -0.915104516 |
| Decitabine | DNA Methyltransferase | 0.126 | -0.899816846 |
| Nicotine Ditartrate | nAChR | 0.126 | -0.898390504 |
| Pramiracetam | others | 0.129 | -0.890819512 |
| Aluminium hydroxide | others | 0.129 | -0.890420549 |
| Acetazolamide | Carbonic Anhydrase | 0.129 | -0.888518396 |
| Zidovudine | Reverse Transcriptase | 0.130 | -0.884943583 |
| Fosfomycin Tromethamine | others | 0.135 | -0.870000977 |
| Amlodipine Besylate | Calcium Channel | 0.136 | -0.866966388 |
| Sodium 4-aminohippurate Hydrate | others | 0.137 | -0.862250012 |
| Piperacillin Sodium | Anti-infection | 0.138 | -0.859327815 |

**Supplementary table 2. Antibodies used in this study.**

| Antibodies | SOURCE | Cat Number | Application | RRID |
| --- | --- | --- | --- | --- |
| β-actin | Cell Signaling Technology | 3700S | WB | AB_2242334 |
| YTHDC1 | Proteintech | 14392-1-AP | WB, RIP, IHC | AB_2878052 |
| AR | Proteintech | 22089-1-AP | WB | AB_11182176 |
| AR | Abcam | ab108341 | ChIP, IHC | AB_10865716 |
| CYP17A1 | Proteintech | 14447-1-AP | WB, IHC | AB_2292527 |
| ACAT1 | Proteintech | 16215-1-AP | WB, IHC | AB_2220210 |
| HMGCR | Proteintech | 13533-1-AP | WB, IHC | AB_2877957 |
| Ki-67 | ZSGB-BIO | ZM-0167 | IHC | AB_2920617 |

**Supplementary table 3.** **The list of nucleic acid sequences for siRNA.**

| Gene | Sequence |
| --- | --- |
| si-AR#1 | CAAGGGAGGTTACACCAAA |
| si-AR#2 | TCTCTTCACAGCCGAAGAA |
| SNORA80B-ASO#1 | TTATGACAGGCCCGTCACCC |
| SNORA80B-ASO | GTACTATGACAGGCCCAGGG |
| si-YTHDC1#1 | GGAAGAAGTGAACTCTGAATT |
| si-YTHDC1#2 | CCATGAGAATGTGTCTCTTTT |

**Supplementary table 4. The list of sequences for RT-qPCR primers.**

| Gene | Sequence (5’-3’) | Application |
| --- | --- | --- |
| SNORA80B | F: CATGCCAGAGCAAACTGTGTC | qPCR |
|  | R: TGCAACCCACTTCAGTGCCA |  |
| U6 | F: CTCGCTTCGGCAGCACA | qPCR |
|  | R: AACGCTTCACGAATTTGCGT |  |
| AR | F: TGTAAGGCAGTGTCGGTGTC | qPCR |
|  | R: GAAGCTGTTCCCCTGGACTC |  |
| ODC1 | F: TGATGCCCGCTGTGTTTTTG | qPCR |
|  | R: AACTGCAAGCGTGAAAGCTG |  |
| YTHDC1 | F: AGTGGACAGACGATTTTCAGGA | qPCR |
|  | R: CTTGAGGAGGTGGAGCATGG |  |
| CYP17A1 | F: CGGCCTCAAATGGCAACTCT | qPCR |
|  | R: GCCACGAAGACAGGAAAGGA |  |
| ACAT1 | F: TTCTTAATTTTAGGATGTCTGGAGC | qPCR |
|  | R: CTGAATTAGCATGGCAGAAGCA |  |
| HMGCR | F: TGCAGCAAACATTGTCACCG | qPCR |
|  | R: CCATTACGGTCCCACACACA |  |
| GAPDH | F: TGTTGCCATCAATGACCCCTT | qPCR |
|  | R: CTCCACGACGTACTCAGCG |  |
| lncRNA‐NEAT1 | F: AACGCTTTATTTTCCAGGTGGCA | qPCR |
|  | R: CGGGCTTACCAGATGACCAG |  |
| 18S | F: CAGCCACCCGAGATTGAGCA | qPCR |
|  | R: TAGTAGCGACGGGCGGTGT |  |
| ABCB1 | F: AGGCTGTCTAACAAGGGCAC | qPCR |
|  | R: GGAGGCCAACATACATGCCT |  |
| ABCC1 | F: CCATGAATGTGCAGAAGGCG | qPCR |
|  | R: CGGATGGTGGACTGGATGAG |  |
| ABCC2 | F: CACAAACCCTGAACTGGCTG | qPCR |
|  | R: AGGGTGCCTCATTTTCCACT |  |
| ERCC1 | F: ACAAAACGGACAGTCAGACCC | qPCR |
|  | R: AACTCCTTGGGTTCTTTCCCAG |  |
| BRCA1 | F: CCGAAGAGGGGCCAAGAAAT | qPCR |
|  | R: ACAGACACTCGGTAGCAACG |  |
| BRCA2 | F: ACTGAGTATTTGGCGTCCATCA | qPCR |
|  | R: CCCGTGGCTGGTAAATCTGA |  |
| BCL2 | F: GAACTGGGGGAGGATTGTGG | qPCR |
|  | R: CCGTACAGTTCCACAAAGGC |  |
| XIAP | F: CGAGGAACCCTGCCATGTAT | qPCR |
|  | R: TGACCAGGCACGATCACAAG |  |
| Survivin | F: CTCAAGGACCACCGCATCTC | qPCR |
|  | R: GTTCCTCTATGGGGTCGTCATC |  |
| GSTP | F: TATTTCCCAGTTCGAGGCCG | qPCR |
|  | R: TACAGGGTGAGGTCTCCGTC |  |
| SNORA80B | F: TCGGCCATGACTTAGAGGTG | ChIP-qPCR |
|  | R: TCCTGAGAGGTTCTGCCTTCT |  |
| ACAT1 | F1: GCCATTGAAAAGGCAGGG | RIP/meRIP-qPCR for KYSE150 |
|  | R1: CCCCACCATATGGTGTTG |  |
| ACAT1 | F2: CTGACGCTGCTGTAGAACCT | RIP/meRIP-qPCR for KYSE450 |
|  | R2: TGACCAACAATCCTGGCTCC |  |
| HMGCR | F: CCTACTACCTCAGCAAGCCT | RIP/meRIP-qPCR |
|  | R: GGCTGTCTTCTTGGTGCAAG |  |
| CYP17A1 | F: CCGGAGTGACTCTATCACC | RIP/meRIP-qPCR |
|  | R: CCTGAGGATTGTGCAGCAG |  |

**Supplementary table 5 The clinical characteristics of the cohort evaluated for SNORA80B detection and survival.**

**Cohort 1**

| No. | Gender | Age | Tumor location | Histologic grade | pT category | Expression of adjacent | Expression of tumor |
| --- | --- | --- | --- | --- | --- | --- | --- |
| 1 | Female | 53 | Lower | Moderate | T2 | 0.00002338 | 0.00000223 |
| 2 | Male | 64 | Lower | Moderate | T2 | 0.00002274 | 0.00000254 |
| 3 | Female | 63 | Middle | Moderate | T2 | 0.00002046 | 0.00000264 |
| 4 | Female | 64 | Middle | Poorly | T2 | 0.00002022 | 0.00000362 |
| 5 | Male | 67 | Middle | Moderate | T3 | 0.00001619 | 0.00000407 |
| 6 | Male | 51 | Middle | Poorly | T2 | 0.00001582 | 0.00000466 |
| 7 | Female | 63 | Middle | Moderate | T3 | 0.00001522 | 0.00029492 |
| 8 | Male | 58 | Middle | Poorly | T3 | 0.00001515 | 0.00002917 |
| 9 | Female | 66 | Middle | Moderate | T2 | 0.00001445 | 0.00002912 |
| 10 | Female | 65 | Middle | Moderate | T2 | 0.00001444 | 0.00001237 |
| 11 | Male | 51 | Middle | Poorly | T3 | 0.00001385 | 0.00001327 |
| 12 | Male | 51 | Middle | Moderate | T3 | 0.00001382 | 0.00001162 |
| 13 | Male | 57 | Middle | Moderate | T2 | 0.00001117 | 0.00001218 |
| 14 | Male | 66 | Middle | Moderate | T3 | 0.00001105 | 0.00001231 |
| 15 | Female | 46 | Lower | Moderate | T3 | 0.00000968 | 0.00018674 |
| 16 | Male | 61 | Middle | Poorly | T3 | 0.00000955 | 0.00018997 |
| 17 | Male | 65 | Middle | Moderate | T3 | 0.00000858 | 0.00019049 |
| 18 | Male | 63 | Lower | Moderate | T3 | 0.00000792 | 0.00019842 |
| 19 | Male | 57 | Upper | Poorly | T2 | 0.00000709 | 0.00020024 |
| 20 | Female | 55 | Middle | Moderate | T3 | 0.00000192 | 0.00024107 |
| 21 | Male | 60 | Lower | Moderate | T3 | 0.00002229 | 0.00024307 |
| 22 | Female | 36 | Upper | Poorly | T3 | 0.00002974 | 0.00041308 |
| 23 | Male | 58 | Middle | Poorly | T3 | 0.00000706 | 0.00043159 |
| 24 | Male | 52 | Middle | Poorly | T3 | 0.00000627 | 0.00045542 |
| 25 | Male | 41 | Middle | Poorly | T3 | 0.00000592 | 0.00001946 |
| 26 | Female | 66 | Middle | Poorly | T2 | 0.00000935 | 0.00002943 |
| 27 | Female | 61 | Middle | Poorly | T2 | 0.00000783 | 0.00002998 |
| 28 | Female | 61 | Upper | Well | T2 | 0.00000978 | 0.00003075 |
| 29 | Male | 52 | Middle | Moderate | T3 | 0.00040668 | 0.00003152 |
| 30 | Male | 47 | Middle | Moderate | T3 | 0.00040644 | 0.00003237 |
| 31 | Male | 59 | Middle | Moderate | T3 | 0.00038768 | 0.00003239 |
| 32 | Male | 61 | Middle | Moderate | T2 | 0.00037585 | 0.00003435 |
| 33 | Female | 57 | Middle | Moderate | T3 | 0.00035730 | 0.00003627 |
| 34 | Female | 62 | Middle | Moderate | T2 | 0.00023978 | 0.00003685 |
| 35 | Male | 65 | Middle | Poorly | T3 | 0.00023918 | 0.00013904 |
| 36 | Male | 69 | Middle | Poorly | T3 | 0.00022509 | 0.00014053 |
| 37 | Male | 62 | Middle | Moderate | T3 | 0.00011816 | 0.00014541 |
| 38 | Male | 61 | Middle | Poorly | T3 | 0.00011254 | 0.00014642 |
| 39 | Male | 69 | Middle | Moderate | T3 | 0.00011014 | 0.00015039 |
| 40 | Male | 77 | Middle | Moderate | T3 | 0.00010475 | 0.00015528 |
| 41 | Male | 75 | Lower | Moderate | T3 | 0.00010470 | 0.00016074 |
| 42 | Female | 54 | Middle | Well | T3 | 0.00007193 | 0.00018035 |
| 43 | Male | 59 | Middle | Moderate | T3 | 0.00002361 | 0.00004299 |
| 44 | Male | 46 | Lower | Poorly | T3 | 0.00002307 | 0.00004619 |
| 45 | Male | 55 | Lower | Moderate | T3 | 0.00002162 | 0.00004802 |
| 46 | Male | 66 | Lower | Moderate | T3 | 0.00002061 | 0.00005014 |
| 47 | Male | 68 | Lower | Moderate | T3 | 0.00002056 | 0.00005079 |
| 48 | Female | 63 | Middle | Poorly | T2 | 0.00002020 | 0.00005345 |
| 49 | Male | 64 | Middle | Poorly | T3 | 0.00001466 | 0.00006844 |
| 50 | Male | 70 | Middle | Poorly | T3 | 0.00001080 | 0.00030978 |
| 51 | Female | 66 | Middle | Moderate | T3 | 0.00001079 | 0.00031265 |
| 52 | Male | 62 | Middle | Moderate | T3 | 0.00000982 | 0.00001340 |
| 53 | Female | 63 | Middle | Poorly | T3 | 0.00000972 | 0.00001677 |
| 54 | Male | 48 | Lower | Moderate | T3 | 0.00000959 | 0.00001749 |
| 55 | Male | 54 | Middle | Poorly | T3 | 0.00000804 | 0.00001773 |
| 56 | Female | 61 | Middle | Moderate | T3 | 0.00000191 | 0.00027186 |
| 57 | Male | 56 | Middle | Moderate | T3 | 0.00000103 | 0.00028758 |
| 58 | Female | 52 | Middle | Moderate | T2 | 0.00000075 | 0.00000503 |
| 59 | Female | 41 | Middle | Moderate | T2 | 0.00000687 | 0.00000645 |
| 60 | Male | 50 | Middle | Moderate | T3 | 0.00000308 | 0.00000670 |
| 61 | Male | 42 | Middle | Moderate | T3 | 0.00000939 | 0.00001123 |
| 62 | Male | 44 | Middle | Poorly | T3 | 0.00000729 | 0.00001156 |
| 63 | Male | 70 | Middle | Moderate | T3 | 0.00000717 | 0.00034963 |
| 64 | Male | 58 | Middle | Poorly | T2 | 0.00000654 | 0.00035549 |
| 65 | Male | 58 | Middle | Moderate | T3 | 0.00040494 | 0.00001820 |
| 66 | Male | 72 | Middle | Moderate | T3 | 0.00037609 | 0.00001834 |
| 67 | Female | 61 | Middle | Moderate | T3 | 0.00023699 | 0.00001913 |
| 68 | Male | 64 | Middle | Poorly | T3 | 0.00022575 | 0.00049642 |
| 69 | Male | 56 | Middle | Moderate | T2 | 0.00021540 | 0.00056966 |
| 70 | Male | 58 | Middle | Poorly | T3 | 0.00020145 | 0.00062939 |
| 71 | Male | 63 | Middle | Poorly | T3 | 0.00020130 | 0.00065603 |
| 72 | Male | 51 | Middle | Poorly | T3 | 0.00013265 | 0.00067022 |
| 73 | Male | 66 | Middle | Poorly | T3 | 0.00012365 | 0.00075620 |
| 74 | Male | 63 | Upper | Poorly | T3 | 0.00006887 | 0.00084633 |
| 75 | Male | 52 | Middle | Moderate | T3 | 0.00000005 | 0.00000007 |
| 76 | Male | 64 | Lower | Moderate | T3 | 0.00000008 | 0.00000235 |
| 77 | Male | 50 | Middle | Moderate | T2 | 0.00000005 | 0.00000006 |
| 78 | Female | 63 | Middle | Moderate | T3 | 0.00000002 | 0.00000148 |
| 79 | Male | 53 | Middle | Moderate | T2 | 0.00000002 | 0.00000038 |
| 80 | Female | 40 | Middle | Moderate | T2 | 0.00000002 | 0.00000002 |
| 81 | Male | 51 | Middle | Poorly | T3 | 0.00000002 | 0.00000249 |
| 82 | Female | 59 | Middle | Moderate | T3 | 0.00000005 | 0.00000049 |

**Cohort 2**

| No. | Differentiation | Gender | Age | pT category | Status | Months | Expression of SNORA80B |
| --- | --- | --- | --- | --- | --- | --- | --- |
| 1 | Well | Male | 50 | T1 |  | 12 | 0.00001010 |
| 2 | Well | Male | 54 | T1 | Live | 49 | 0.00000956 |
| 3 | Well | Male | 54 | T1 | Live | 40 | 0.00000953 |
| 4 | Well | Male | 41 | T1 |  | 26 | 0.00000927 |
| 5 | Well | Female | 62 | T1 | Dead | 142 | 0.00000701 |
| 6 | Well | Male | 51 | T1 | Live | 120 | 0.00000700 |
| 7 | Well | Male | 40 | T1 | Live | 118 | 0.00000650 |
| 8 | Well | Male | 61 | T1 | Dead | 40 | 0.00000602 |
| 9 | Well | Male | 48 | T1 | Live | 60 | 0.00000557 |
| 10 | Well | Male | 67 | T1 | Live | 15 | 0.00000523 |
| 11 | Well | Male | 62 | T1 | Live | 183 | 0.00000485 |
| 12 | Well | Male | 48 | T1 | Dead | 32 | 0.00000480 |
| 13 | Well | Male | 47 | T1 | Live | 64 | 0.00000477 |
| 14 | Well | Female | 43 | T1 | Live | 24 | 0.00000448 |
| 15 | Well | Female | 55 | T1 | Dead | 59 | 0.00000429 |
| 16 | Well | Female | 57 | T1 | Live | 61 | 0.00000418 |
| 17 | Well | Male | 62 | T1 | Live | 26 | 0.00000403 |
| 18 | Well | Male | 58 | T1 | Live | 184 | 0.00000401 |
| 19 | Well | Male | 63 | T1 | Live | 183 | 0.00000382 |
| 20 | Well | Male | 54 | T1 | Dead | 161 | 0.00000377 |
| 21 | Well | Female | 58 | T1 | Dead | 47 | 0.00000311 |
| 22 | Well | Male | 67 | T1 | Live | 101 | 0.00000300 |
| 23 | Well | Male | 46 | T1 | Live | 144 | 0.00000281 |
| 24 | Well | Female | 58 | T1 | Live | 96 | 0.00000277 |
| 25 | Well | Female | 61 | T1 | Live | 92 | 0.00000223 |
| 26 | Well | Male | 60 | T1 | Live | 80 | 0.00000205 |
| 27 | Well | Female | 43 | T1 | Dead | 14 | 0.00000188 |
| 28 | Well | Male | 49 | T1 | Live | 67 | 0.00000140 |
| 29 | Well | Female | 58 | T1 | Live | 62 | 0.00000118 |
| 30 | Well | Female | 64 | T1 | Live | 53 | 0.00000100 |
| 31 | Well | Male | 58 | T1 | Live | 114 | 0.00000078 |
| 32 | Moderate | Male | 63 | T1 | Live | 50 | 0.00003054 |
| 33 | Moderate | Male | 42 | T1 | Live | 139 | 0.00002665 |
| 34 | Moderate | Male | 40 | T1 |  | 24 | 0.00002419 |
| 35 | Moderate | Male | 52 | T1 | Live | 109 | 0.00001857 |
| 36 | Moderate | Female | 44 | T1 |  | 27 | 0.00001691 |
| 37 | Moderate | Male | 51 | T1 |  | 25 | 0.00001640 |
| 38 | Moderate | Female | 50 | T1 | Live | 89 | 0.00001458 |
| 39 | Moderate | Female | 50 | T1 | Live | 51 | 0.00001426 |
| 40 | Moderate | Male | 55 | T1 |  | 5 | 0.00001404 |
| 41 | Moderate | Male | 54 | T1 | Dead | 54 | 0.00001347 |
| 42 | Moderate | Male | 59 | T1 |  | 50 | 0.00001331 |
| 43 | Moderate | Male | 51 | T1 | Dead | 73 | 0.00001312 |
| 44 | Moderate | Male | 48 | T1 | Dead | 4 | 0.00001290 |
| 45 | Moderate | Male | 43 | T1 | Live | 136 | 0.00001274 |
| 46 | Moderate | Male | 54 | T1 |  | 15 | 0.00001179 |
| 47 | Moderate | Male | 65 | T1 | Live | 82 | 0.00001072 |
| 48 | Moderate | Male | 64 | T1 | Dead | 67 | 0.00001063 |
| 49 | Moderate | Male | 70 | T1 | Dead | 8 | 0.00001039 |
| 50 | Moderate | Male | 56 | T1 | Live | 24 | 0.00000979 |
| 51 | Moderate | Female | 63 | T1 | Dead | 34 | 0.00000979 |
| 52 | Moderate | Male | 52 | T1 | Live | 51 | 0.00000725 |
| 53 | Moderate | Female | 55 | T1 | Live | 66 | 0.00000678 |
| 54 | Moderate | Male | 47 | T1 | Live | 19 | 0.00000663 |
| 55 | Moderate | Male | 68 | T1 | Live | 22 | 0.00000637 |
| 56 | Moderate | Male | 46 | T1 | Live | 200 | 0.00000612 |
| 57 | Moderate | Male | 65 | T1 | Dead | 162 | 0.00000530 |
| 58 | Moderate | Male | 64 | T1 | Live | 12 | 0.00000528 |
| 59 | Moderate | Female | 37 | T1 | Live | 216 | 0.00000521 |
| 60 | Moderate | Male | 42 | T1 | Live | 60 | 0.00000503 |
| 61 | Moderate | Male | 57 | T1 | Live | 76 | 0.00000471 |
| 62 | Moderate | Male | 42 | T1 | Live | 135 | 0.00000471 |
| 63 | Moderate | Male | 52 | T1 | Dead | 52 | 0.00000467 |
| 64 | Moderate | Male | 49 | T1 | Live | 154 | 0.00000457 |
| 65 | Moderate | Male | 54 | T1 | Dead | 12 | 0.00000456 |
| 66 | Moderate | Male | 62 | T1 | Live | 11 | 0.00000455 |
| 67 | Moderate | Male | 44 | T1 | Live | 28 | 0.00000375 |
| 68 | Moderate | Male | 50 | T1 | Live | 48 | 0.00000370 |
| 69 | Moderate | Female | 50 | T1 | Dead | 39 | 0.00000347 |
| 70 | Moderate | Male | 69 | T1 | Live | 108 | 0.00000317 |
| 71 | Moderate | Female | 67 | T1 | Live | 58 | 0.00000314 |
| 72 | Moderate | Male | 66 | T1 | Live | 104 | 0.00000312 |
| 73 | Moderate | Male | 61 | T1 | Live | 86 | 0.00000300 |
| 74 | Moderate | Female | 57 | T1 | Dead | 26 | 0.00000290 |
| 75 | Moderate | Male | 64 | T1 | Live | 94 | 0.00000268 |
| 76 | Moderate | Male | 38 | T1 | Live | 117 | 0.00000265 |
| 77 | Moderate | Male | 50 | T1 | Dead | 31 | 0.00000213 |
| 78 | Moderate | Male | 68 | T1 | Live | 84 | 0.00000211 |
| 79 | Moderate | Male | 68 | T1 | Live | 81 | 0.00000211 |
| 80 | Moderate | Male | 52 | T1 | Live | 71 | 0.00000188 |
| 81 | Moderate | Male | 61 | T1 | Live | 72 | 0.00000177 |
| 82 | Moderate | Male | 52 | T1 | Live | 135 | 0.00000124 |
| 83 | Moderate | Male | 61 | T1 | Dead | 40 | 0.00000104 |
| 84 | Poorly | Male | 48 | T1 |  | 20 | 0.00003885 |
| 85 | Poorly | Female | 71 | T1 | Dead | 50 | 0.00003741 |
| 86 | Poorly | Male | 64 | T1 | Dead | 5 | 0.00003732 |
| 87 | Poorly | Female | 42 | T1 | Dead | 32 | 0.00003550 |
| 88 | Poorly | Male | 64 | T1 | Dead | 45 | 0.00003228 |
| 89 | Poorly | Male | 52 | T1 |  |  | 0.00003173 |
| 90 | Poorly | Male | 54 | T1 | Live | 84 | 0.00002401 |
| 91 | Poorly | Male | 65 | T1 | Dead | 12 | 0.00002113 |
| 92 | Poorly | Male | 50 | T1 | Live | 104 | 0.00001720 |
| 93 | Poorly | Male | 65 | T1 |  |  | 0.00001665 |
| 94 | Poorly | Male | 36 | T1 |  |  | 0.00001323 |
| 95 | Poorly | Male | 49 | T1 |  | 20 | 0.00001142 |
| 96 | Poorly | Female | 74 | T1 | Dead | 12 | 0.00001114 |
| 97 | Poorly | Male | 65 | T1 | Dead | 22 | 0.00000964 |
| 98 | Poorly | Male | 51 | T1 | Live | 50 | 0.00000913 |
| 99 | Poorly | Male | 61 | T1 | Live | 65 | 0.00000911 |
| 100 | Poorly | Female | 70 | T1 | Live | 36 | 0.00000818 |
| 101 | Poorly | Male | 59 | T1 | Dead | 42 | 0.00000814 |
| 102 | Poorly | Male | 56 | T1 | Dead | 26 | 0.00000795 |
| 103 | Poorly | Male | 43 | T1 | Dead | 32 | 0.00000770 |
| 104 | Poorly | Male | 54 | T1 | Live | 3 | 0.00000577 |
| 105 | Poorly | Male | 39 | T1 | Live | 18 | 0.00000535 |
| 106 | Poorly | Female | 56 | T1 | Live | 61 | 0.00000425 |
| 107 | Poorly | Female | 52 | T1 | Live | 126 | 0.00000395 |
| 108 | Poorly | Male | 46 | T1 | Live | 116 | 0.00000330 |
| 109 | Poorly | Male | 56 | T1 | Dead | 14 | 0.00000328 |
| 110 | Poorly | Male | 46 | T1 | Live | 19 | 0.00000291 |
| 111 | Poorly | Male | 59 | T1 | Dead | 62 | 0.00000288 |
| 112 | Poorly | Female | 67 | T1 | Live | 61 | 0.00000272 |
| 113 | Poorly | Male | 63 | T1 | Live | 88 | 0.00000251 |
| 114 | Poorly | Male | 69 | T1 | Live | 88 | 0.00000221 |
| 115 | Poorly | Female | 49 | T1 | Live | 87 | 0.00000216 |
| 116 | Poorly | Female | 50 | T1 | Live | 144 | 0.00000185 |
| 117 | Poorly | Female | 62 | T1 | Live | 72 | 0.00000172 |
| 118 | Poorly | Male | 53 | T1 | Live | 72 | 0.00000171 |
| 119 | Poorly | Female | 48 | T1 | Live | 69 | 0.00000164 |
| 120 | Poorly | Male | 53 | T1 | Live | 67 | 0.00000145 |
| 121 | Poorly | Male | 77 | T1 | Live | 67 | 0.00000127 |
