# Supplementary material for: Clinofibrate Disrupts the SNORA80B/YTHDC1‐Driven M6A Modification to Suppress Cholesterol Metabolism and Cisplatin Resistance in ESCC
Source: Adv Sci (Weinh). 2025 Nov 3;13(3):e09574. doi: 10.1002/advs.202509574 (PMC12884767; doi:10.1002/advs.202509574)
Supplement: Supplementary file 2 — Supporting Information [file ADVS-13-e09574-s002.pdf]

STR 基因型检测报告

样本名称：HEK-293T

检测方法： 用 Axygen 的基因组抽提试剂盒提取 DNA，采用 20- STR 扩增方案扩增，在 ABI 3730XL 型遗传分析仪上对 STR 位点和性别基因 Amelogenin 进行检测。

检测结果： 该株细胞 DNA 分型在细胞系检索中找到**完全匹配**的细胞系，DSMZ 数据库显示细胞名为 **HEK- 293T**，细胞号对应 **635**。本次检测在该细胞系中**发现多等位基因**。细胞匹配值：1.0

STR 数据库比对分析： 待测细胞的 STR 位点和 Amelogenin 位点的基因分型结果与收录于 ExPASY,ATCC, DSMZ, JCRB 和 RIKEN 数据库的 2455 个细胞系 STR 数据进行比对，如果待检测细胞未收录于以上细胞库或这是自行建立的新细胞系将无法进行比对，用户需根据细胞分型结果自行与其他数据库进行比对。

分型结果：

Loci	送检细胞 STR 信息			细胞库细胞 STR 信息		
	送检细胞名：HEK-293T			细胞库细胞名：HEK- 293T		
	Allele1	Allele2	Allele3	Allele1	Allele2	Allele3
D5S818	8	9		8	9	
D13S317	12	14		12	14	
D7S820	11	11		11	11	
D16S539	9	13		9	13	
VWA	16	18	19	16	19	
TH01	7	9.3		7	9.3	
AMEL	X	X		X	X	
TPOX	11	11		11	11	
CSF1PO	11	12		11	12	
D12S391	19	21				
FGA	23	23				
D2S1338	19	19				
D21S11	28	30.2				
D18S51	17	18				
D8S1179	12	14				
D3S1358	15	17				
D6S1043	11	11				
PENTAE	7	15				
D19S433	18	18				
PENTAD	9	10				
D1S1656	15	17.3				

分型图谱：

EV	Cell No.	Cell name	Locus names									Figures
			D5S818	D13S317	D7S820	D16S539	VWA	TH01	AM	TPOX	CSF1PO	
	Query (Your Cell)		*8,9*	*12,14*	*11,11*	*9,13*	*16,18,19*	*7,9.3*	*X,X*	*11,11*	*11,12*	
1.00(38/38)	CRL-11268	293T/17 [HEK 293T/17]	8,9	12,14	11,11	9,13	16,18,19	7,9.3	X,X	11,11	11,12	-
0.97(38/39)	CRL-11269	ANJOU 65	8,9	11,12,14	11,11	9,13	16,18,19	7,9.3	X,X	11,11	11,12	-
0.97(36/37)	635	293T	8,9	12,14	11,11	9,13	16,19	7,9.3	X,X	11,11	11,12	-

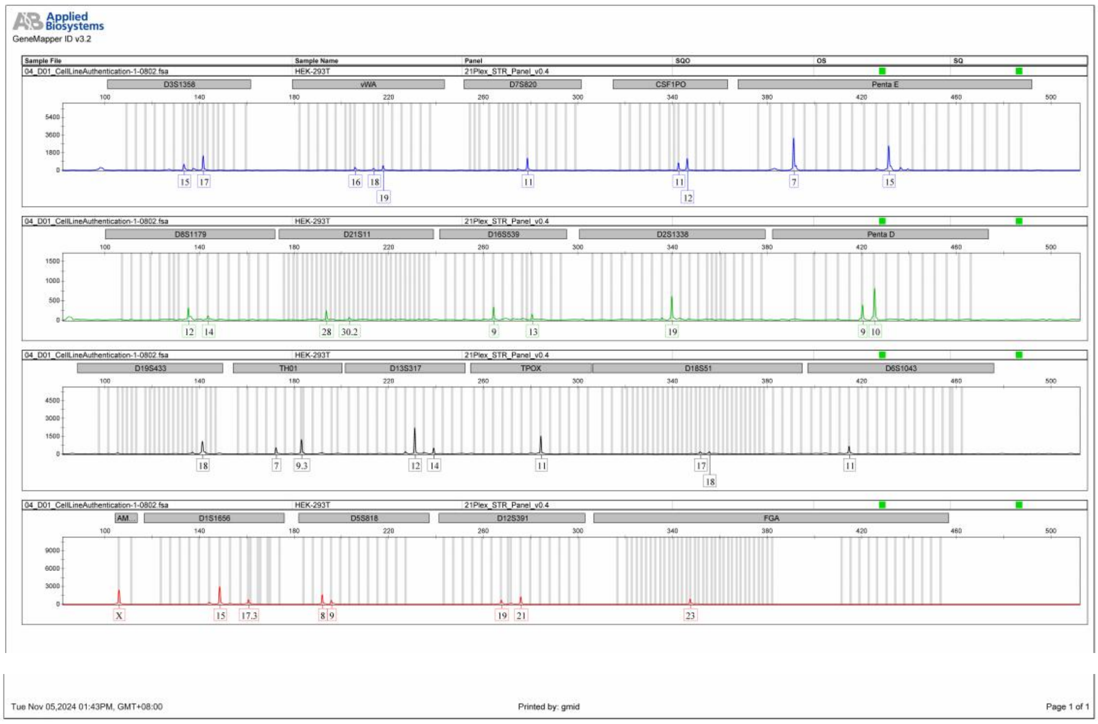

备注：

1. 根据国际细胞鉴定委员会（ICLAC)制定的细胞 STR 鉴定标准，匹配度≥80%即可认为该细胞系正确，匹配度＜80%则说明该细胞系的来源需要被怀疑。
2. 图谱有效峰位真实的 PCR 条带，小峰和非特异性条带在计算中忽略不计。
3. 国家实验细胞资源共享平台，数据库入口 http://www.cellresource.cn /。
