# Supplementary material for: Clinofibrate Disrupts the SNORA80B/YTHDC1‐Driven M6A Modification to Suppress Cholesterol Metabolism and Cisplatin Resistance in ESCC
Source: Adv Sci (Weinh). 2025 Nov 3;13(3):e09574. doi: 10.1002/advs.202509574 (PMC12884767; doi:10.1002/advs.202509574)

Supplementary materials

Figure 3D

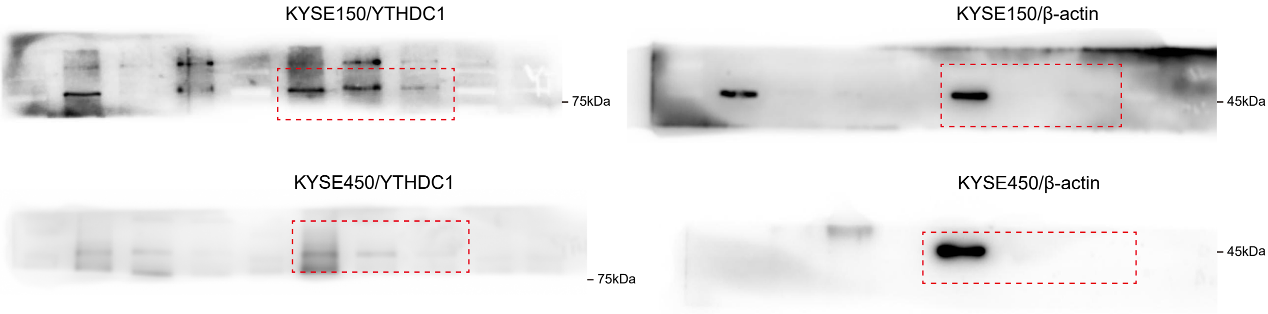

Figure 3E

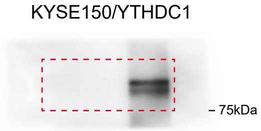

Figure 3F

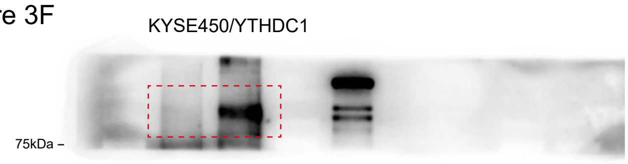

Figure 3G

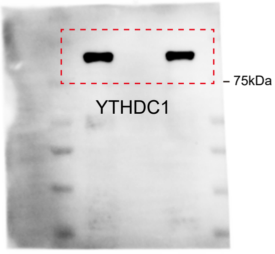

Figure 3H

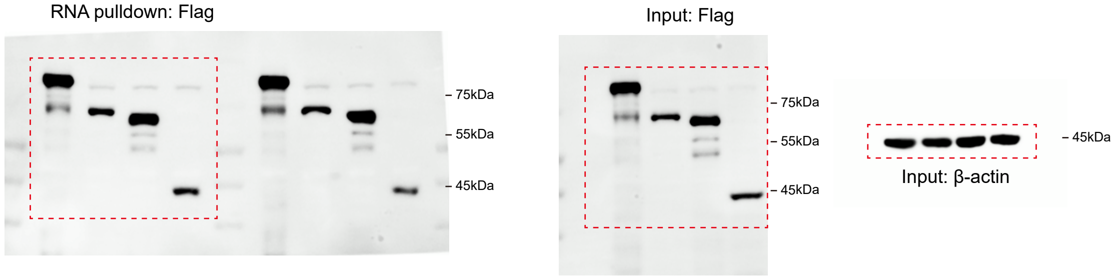

Figure 5M

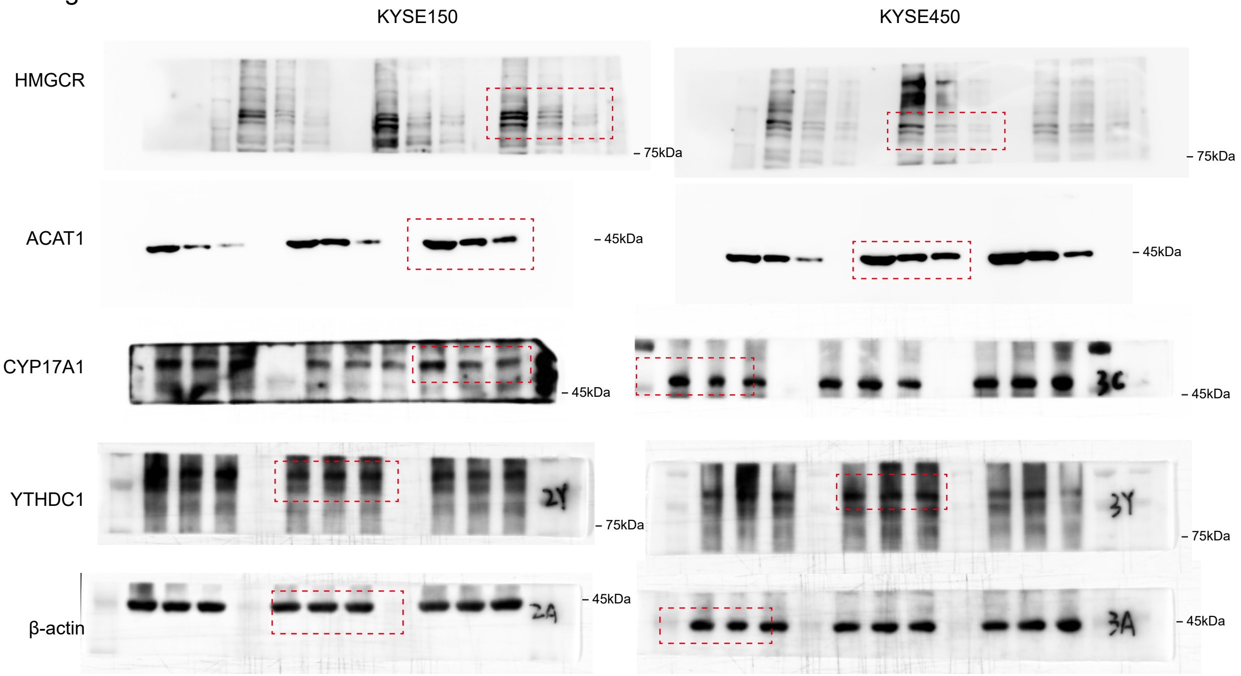

Figure S1B

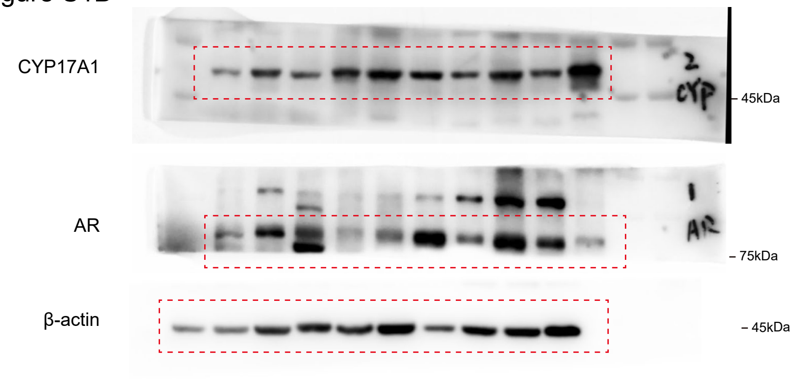

Figure S3D

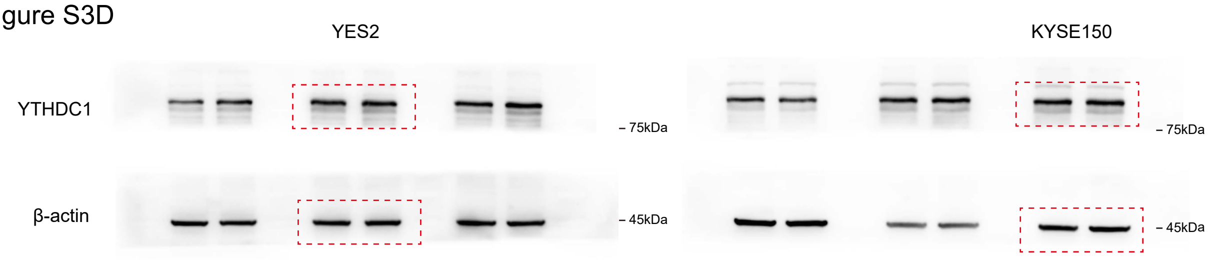

Figure S3G

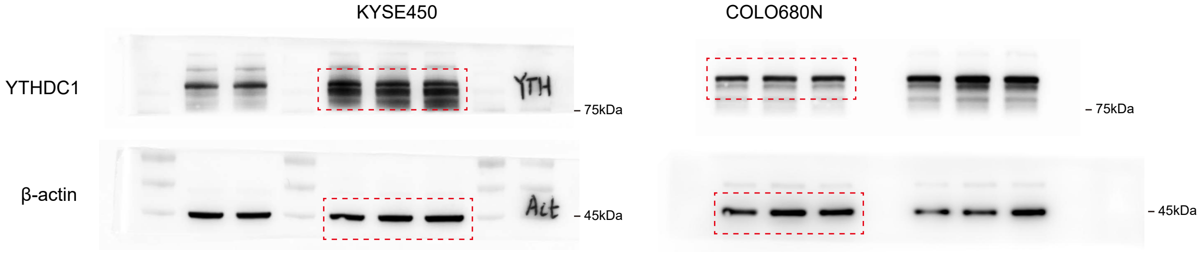

Figure S3H

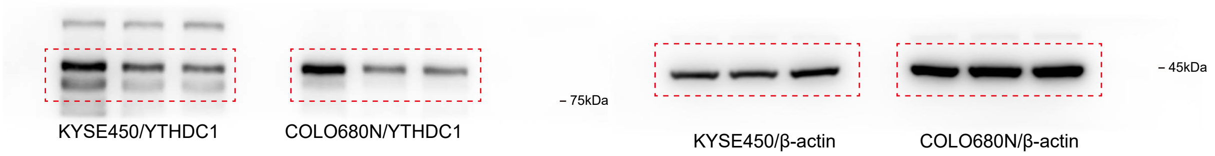

Figure S5D

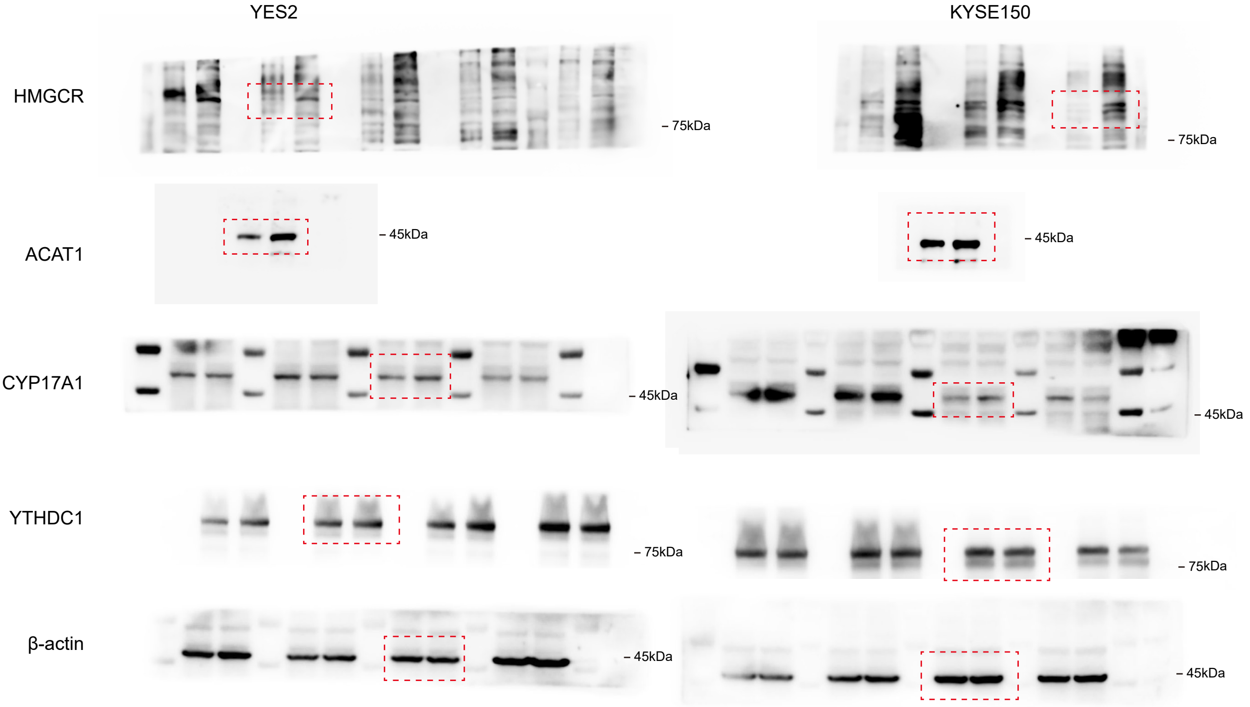

Figure S5E

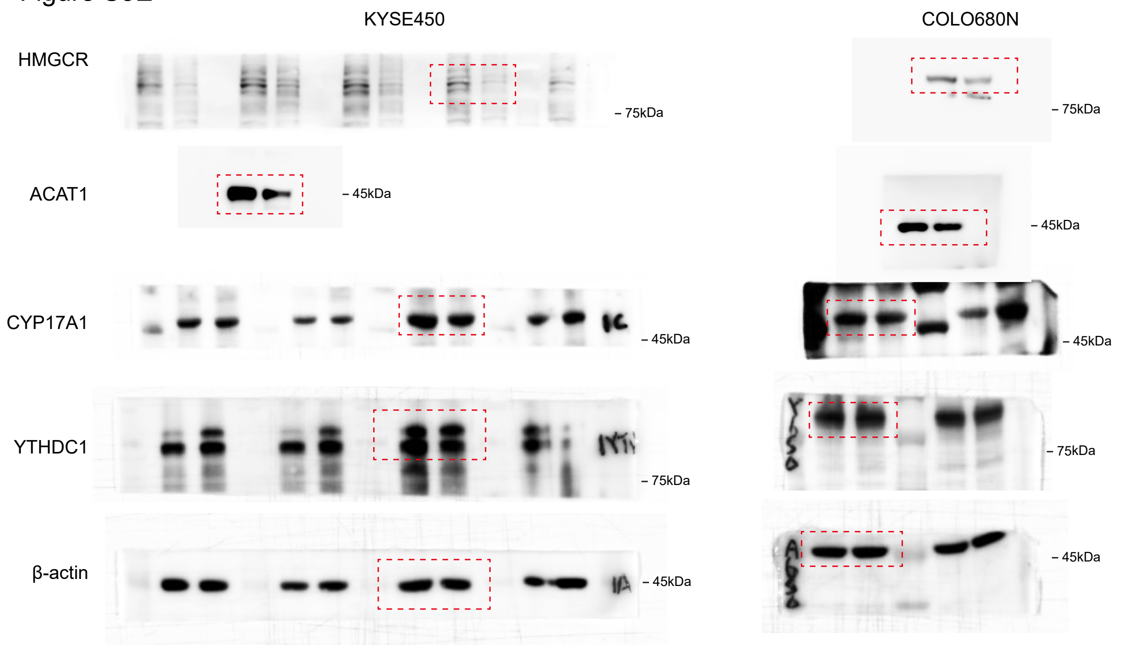

Figure S5F

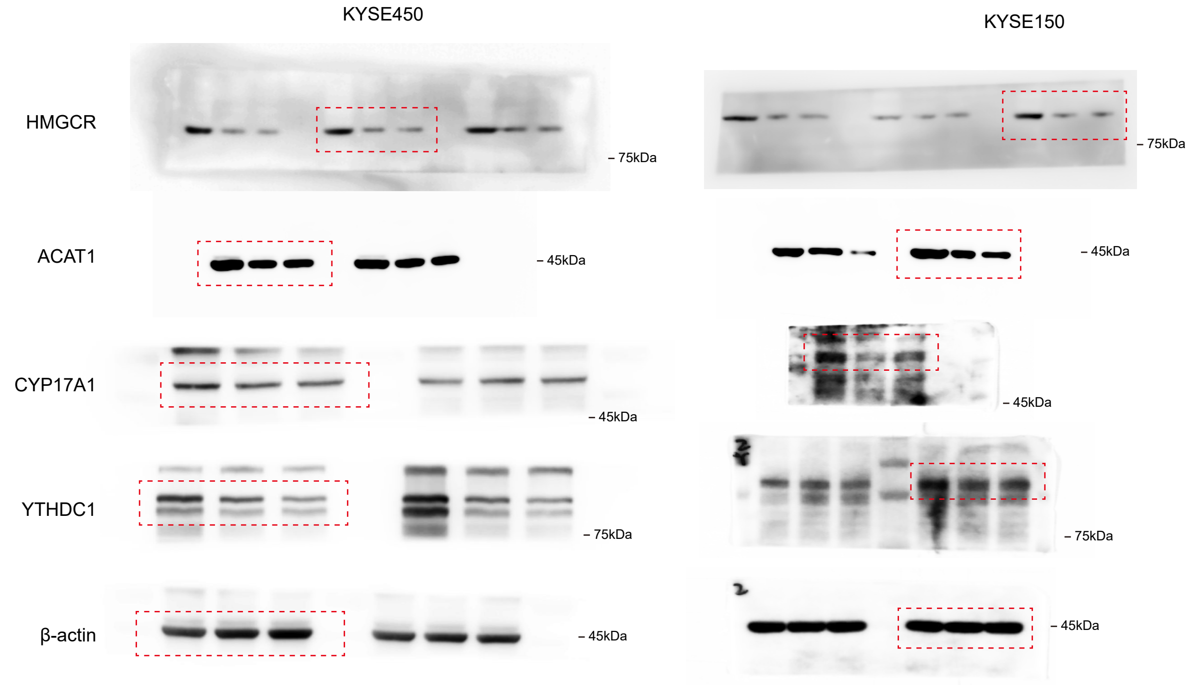

Supplement: Supplementary file 3 — Supporting Information [file ADVS-13-e09574-s003.pdf]
