# Supplementary material for: Clinofibrate Disrupts the SNORA80B/YTHDC1‐Driven M6A Modification to Suppress Cholesterol Metabolism and Cisplatin Resistance in ESCC
Source: Adv Sci (Weinh). 2025 Nov 3;13(3):e09574. doi: 10.1002/advs.202509574 (PMC12884767; doi:10.1002/advs.202509574)

STR 细胞鉴定结题报告

**订单编号：**	LWXB21035
**报告时间：**	2021.07.08

华大基因

项目基本情况			
订单编号	LWXB21035	订单名称	中国医学科学院肿瘤医院赵梓彤 12 个样本细胞鉴定 7.1
客户单位	中国医学科学院肿瘤医院	销售姓名	贺敏
客户姓名	赵梓彤	项目管理	王双玉
客户联系方式	13718847346；zhaozitong880807@126.com		
项目完成情况			
起始日期	2021.07.01	完成日期	2021.07.08
样品数	9 个		
结算金额	人民币柒仟贰佰元整（￥7200）		
备注	目前完成 9 个		

目录

1 实验目的 5

2 实验流程 5

2.1 实验试剂 5

2.2 实验仪器 5

2.3 实验步骤 5

2.4 上机检测 6

3 实验结果 7

3.1 KYSE410 检测结果 7

3.1.1 KYSE410 STR 数据 7
3.1.2 KYSE410 STR 位点数据比对结果 8
3.1.3 KYSE410 STR 图谱 9

3.2 KYSE150 检测结果 10

3.2.1 KYSE150 STR 数据 10
3.2.2 KYSE150 STR 位点数据比对结果 11
3.2.3 KYSE150 STR 图谱 12

3.3 KYSE30 检测结果 13

3.3.1 KYSE30 STR 数据 13
3.3.2 KYSE30 STR 位点数据比对结果 14
3.3.3 KYSE30 STR 图谱 15

3.4 KYSE140 检测结果 16

3.4.1 KYSE140 STR 数据 16
3.4.2 KYSE140 STR 位点数据比对结果 17
3.4.3 KYSE140 STR 图谱 18

3.5 KYSE510 检测结果 19

3.5.1 KYSE510 STR 数据 19
3.5.2 KYSE510 STR 位点数据比对结果 20
3.5.3 KYSE510 STR 图谱 21

3.6 KYSE180 检测结果 22

3.6.1 KYSE180 STR 数据 22
3.6.2 KYSE180 STR 位点数据比对结果 23
3.6.3 KYSE180 STR 图谱 24

3.7 KYSE70 检测结果 25

3.7.1 KYSE70 STR 数据 25
3.7.2 KYSE70 STR 位点数据比对结果 26
3.7.3 KYSE70 STR 图谱 27

3.8 KYSE450 检测结果 28

3.8.1 KYSE450 STR 数据 28
3.8.2 KYSE450 STR 位点数据比对结果 29
3.8.3 KYSE450 STR 图谱 30

3.9 colo680n 检测结果 31

3.9.1 colo680n STR 数据 31
3.9.2 colo680n STR 位点数据比对结果 32
3.9.3 colo680n STR 图谱 33

4. 参考文献 34

1 实验目的

细胞 STR 信息鉴定：

KYSE-410：https://web.expasy.org/cellosaurus/CVCL_1352

KYSE-150：https://web.expasy.org/cellosaurus/CVCL_1348

KYSE-30：https://web.expasy.org/cellosaurus/CVCL_1351

KYSE-140：https://web.expasy.org/cellosaurus/CVCL_1347

KYSE-510：https://web.expasy.org/cellosaurus/CVCL_1354

KYSE-180：https://web.expasy.org/cellosaurus/CVCL_1349

KYSE-70：https://web.expasy.org/cellosaurus/CVCL_1356

KYSE-450：https://web.expasy.org/cellosaurus/CVCL_1353

COLO 680N：https://web.expasy.org/cellosaurus/CVCL_1131

2 实验流程

2.1 实验试剂

华大人类 DNA 分型盒 （炎黄）

2.2 实验仪器

GeneAmp® PCR system9700，ABI3730XL

2.3 实验步骤

DNA 提取后，按以下体系进行反应扩增

ddH2O	2	μl
2X Master Mix	5	μl
4X Primer Pair Mix	2	μl
DNA	1	μl
Total	10	μl

PCR 扩增使用 Applied Biosystems 9700 PCR System 进行扩增，PCR 扩增程序如下：

95℃	10min	
95℃	10 sec	×28Cycle
58℃	1min	×28Cycle
70℃	20 sec	×28Cycle
60℃	15 min	
4℃	∞	

2.4 上机检测

内标混合：将 PCR 产物与 STR 500 内标，HIDI 混合，混合体系为

STR-500	0.5μl
PCR 产物	1μl
HIDI	8.5μL
Total	10μL

将混合物在 PCR 仪上进行热变性（95℃3min40s），冰中骤冷，待上机。

使用仪器为 3730XL 按仪器操作说明书安装毛细管，进行毛细管位置的校正，人工手动灌胶和建立运行的测序文件。仪器将自动灌胶至毛细管，1.2kV 预电泳 5min，按编程次序自动进样，再预电泳（1.2kV，20min），在 7.5kV 下电泳 2h。电泳结束后仪器会自动清洗，灌胶，进下一样品，预电泳和电泳。每一个样品电泳总时间为 2.5h。电泳结束后仪器会自动分析 STR 图谱。

3 实验结果

3.1 KYSE410 检测结果

3.1.1 KYSE410 STR 数据

STR 基因座	KYSE410 检测结果	
	Allele 1	Allele 2
Yindel	-	
AMEL	X	
D3S1358	15	16
D13S317	11	
D7S820	12	
D16S539	10	12
SE33	28.2	31.2
D10S1248	13	
D5S818	13	
D21S11	30	
TPOX	8	11
D1S1656	13	15
D6S1043	13	15
DXS6795	13	
D19S433	13	
D22S1045	15	16
D8S1179	10	
Penta E	8	12
DYS391	-	
D2S441	11	12
D12S391	17	19
D2S1338	17	19
vWA	16	18
Penta D	11	
TH01	8	
D18S51	13	15
CSF1PO	12	
FGA	20	

3.1.2 KYSE410 STR 位点数据比对结果

通过 DSMZ 数据库/客户提供细胞信息比对结果如下

EV	Cell No.	Cell name	Locus names								
			D5S818	D13S317	D7S820	D16S539	VWA	TH01	AM	TPOX	CSF1PO
	Query (Your Cell)		13,13	11,11	12,12	10,12	16,18	8,8	X,X	8,11	12,12
1.00(36/36)	381	KYSE-410	13,13	11,11	12,12	10,12	16,18	8,8	X,X	8,11	12,12
0.83(30/36)	CRL-2335	HCC 1806	13,13	11,11	10,12	10,10	16,18	8,8	X,X	8,9	12,12
0.67(24/36)	87	GDM-1	13,13	11,12	11,12	12,12	16,17	6,6	X,X	8,11	12,12
0.67(24/36)	248	COLO-677	11,13	11,11	9,10	9,9	16,18	8,8	X,Y	8,11	12,12
0.67(24/36)	361	BFTC-905	13,13	11,11	9,12	12,12	14,17	7,8	X,X	8,9	12,12
**1.00(36/36)**	**CVCL_1352**	**KYSE-410**	**13,13**	**11,11**	**12,12**	**10,12**	**16,18**	**8,8**	**X,X**	**8,11**	**12,12**

客户提供数据比对

根据客户提供数据进行比对，KYSE410 样本的 STR 结果与数据相符。

注：参考 ANSI/ATCC,Authenticationof Human Cell Line Standardization of STR Profiling . 2011,ASN-0002-2011.标准，STR 检测匹配度超过 80%即可认为是同一种来源的细胞。

3.1.3 KYSE410 STR 图谱

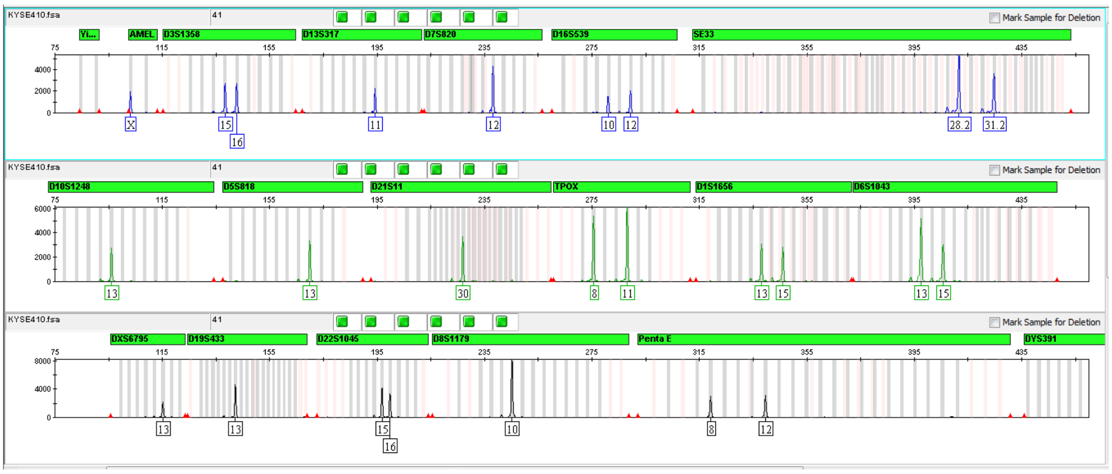

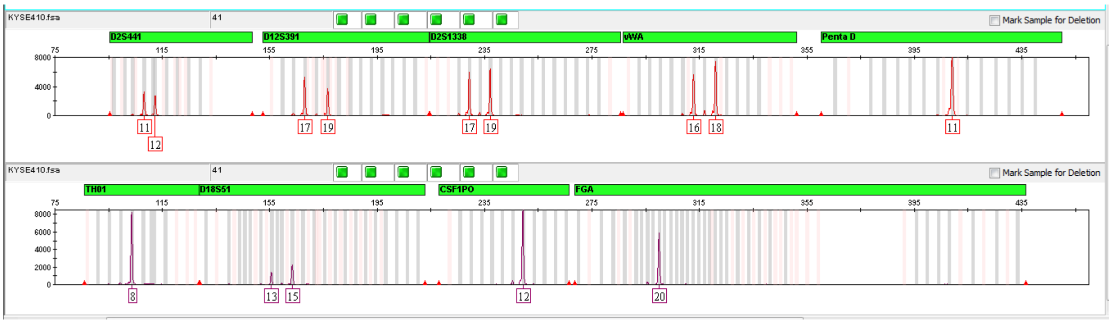

3.2 KYSE150 检测结果

3.2.1 KYSE150 STR 数据

STR 基因座	KYSE150 检测结果	
	Allele 1	Allele 2
Yindel	-	
AMEL	X	
D3S1358	15	16
D13S317	8	11
D7S820	10	11
D16S539	9	11
SE33	20	
D10S1248	13	14
D5S818	12	13
D21S11	30	31
TPOX	8	
D1S1656	15	17.3
D6S1043	18	20
DXS6795	13	
D19S433	15	15.2
D22S1045	15	
D8S1179	10	15
Penta E	12	18
DYS391	-	
D2S441	10	11
D12S391	19	22
D2S1338	25	
vWA	16	17
Penta D	10	
TH01	7	9
D18S51	14	
CSF1PO	12	13
FGA	21	24

3.2.2 KYSE150 STR 位点数据比对结果

通过 DSMZ 数据库/客户提供细胞信息比对结果如下

EV	Cell No.	Cell name	Locus names								
			D5S818	D13S317	D7S820	D16S539	VWA	TH01	AM	TPOX	CSF1PO
	Query (Your Cell)		12,13	8,11	10,11	9,11	16,17	7,9	X,X	8,8	12,13
1.00(36/36)	375	KYSE-150	12,13	8,11	10,11	9,11	16,17	7,9	X,X	8,8	12,13
0.79(30/38)	RCB0608	SF8543	10,11,12	8,11	11,12	9,11	16,17,19	7,9	X,X	8,8	11,13
0.78(28/36)	646	HCEC-12	11,12	8,11	10,11	11,14	14,17	9,9.3	X,X	8,8	12,13
0.78(28/36)	647	HCEC-B4G12	11,12	8,11	10,11	11,14	14,17	9,9.3	X,X	8,8	12,13
0.76(28/37)	RCB0582	SF8404	10,12	8,11	11,11	9,11	16,17	6,9,9.3	X,X	8,11	12,13
**1.00(36/36)**	**CVCL_1348**	**KYSE-150**	**12,13**	**8,11**	**10,11**	**9,11**	**16,17**	**7,9**	**X,X**	**8,8**	**12,13**

客户提供数据比对

根据客户提供数据进行比对，KYSE150 样本的 STR 结果与数据相符。

注：参考 ANSI/ATCC,Authenticationof Human Cell Line Standardization of STR Profiling . 2011,ASN-0002-2011.标准，STR 检测匹配度超过 80%即可认为是同一种来源的细胞。

3.2.3 KYSE150 STR 图谱

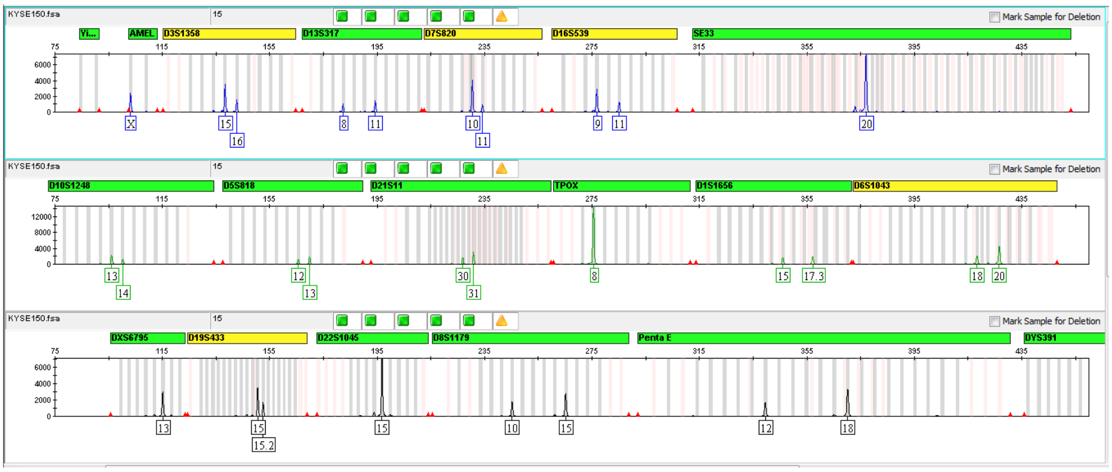

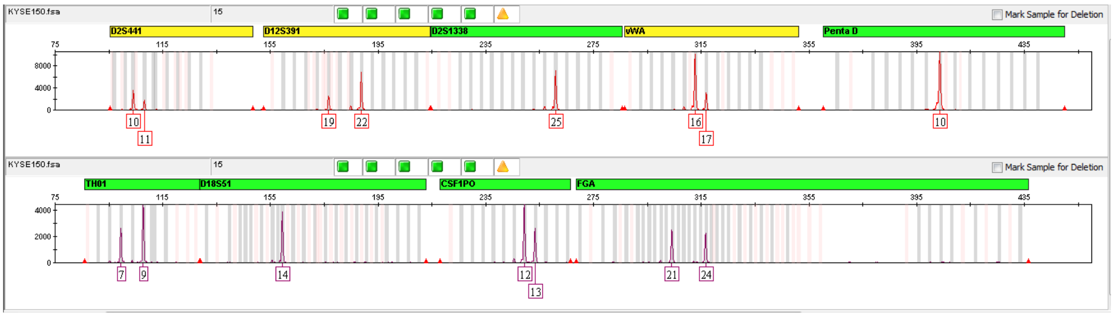

3.3 KYSE30 检测结果

3.3.1 KYSE30 STR 数据

STR 基因座	KYSE30 检测结果		
	Allele 1	Allele 2	Allele 3
Yindel	-		
AMEL	X		
D3S1358	15	16	
D13S317	9		
D7S820	11	11.3	
D16S539	10	12	
SE33	22	23	
D10S1248	15		
D5S818	11		
D21S11	28		
TPOX	9		
D1S1656	15	16	
D6S1043	11	20	
DXS6795	13		
D19S433	14.2	15.2	
D22S1045	11	16	
D8S1179	12	15	
Penta E	13		
DYS391	-		
D2S441	11		
D12S391	17	19	
D2S1338	23		
vWA	16	18	19
Penta D	12		
TH01	9		
D18S51	14		
CSF1PO	10		
FGA	24		

3.3.2 KYSE30 STR 位点数据比对结果

通过 DSMZ 数据库/客户提供细胞信息比对结果如下

EV	Cell No.	Cell name	Locus names								
			D5S818	D13S317	D7S820	D16S539	VWA	TH01	AM	TPOX	CSF1PO
	Query (Your Cell)		11,11	9,9	11,11.3	10,12	16,18,19	9,9	X,X	9,9	10,10
0.95(36/38)	351	KYSE-30	11,11	9,9	11,12	10,12	16,18,19	9,9	X,X	9,9	10,10
0.95(36/38)	JCRB0188	KYSE-30	11,11	9,9	11,11.3	10,12	16,18,19	9,9	X,X	8,9	10,10
0.59(22/37)	CRL-2338	HCC1954	11,11	8,9	10,11	9,11	18,19	6,7	X,X	8,9	10,10
0.59(22/37)	CRL-3035	CHLA-03-AA	11,11	10,11	9,10	11,12	16,18	6,9	X,X	8,9	10,10
0.59(22/37)	JCRB1039	SKN-3	9,9	11,11	10,11	10,12	18,20	9,9	X,X	9,11	10,10
**0.95(36/38)**	**CVCL_1351(DSMZ)**	**KYSE-30**	**11,11**	**9,9**	**11,12**	**10,12**	**16,18,19**	**9,9**	**X,X**	**9,9**	**10,10**
**0.95(36/38)**	**CVCL_1351(JCRB)**	**KYSE-30**	**11,11**	**9,9**	**11,11.3**	**10,12**	**16,18,19**	**9,9**	**X,X**	**8,9**	**10,10**

客户提供数据比对

根据客户提供数据进行比对，KYSE30 样本的 STR 结果与数据相符。

注：参考 ANSI/ATCC,Authenticationof Human Cell Line Standardization of STR Profiling . 2011,ASN-0002-2011.标准，STR 检测匹配度超过 80%即可认为是同一种来源的细胞。

3.3.3 KYSE30 STR 图谱

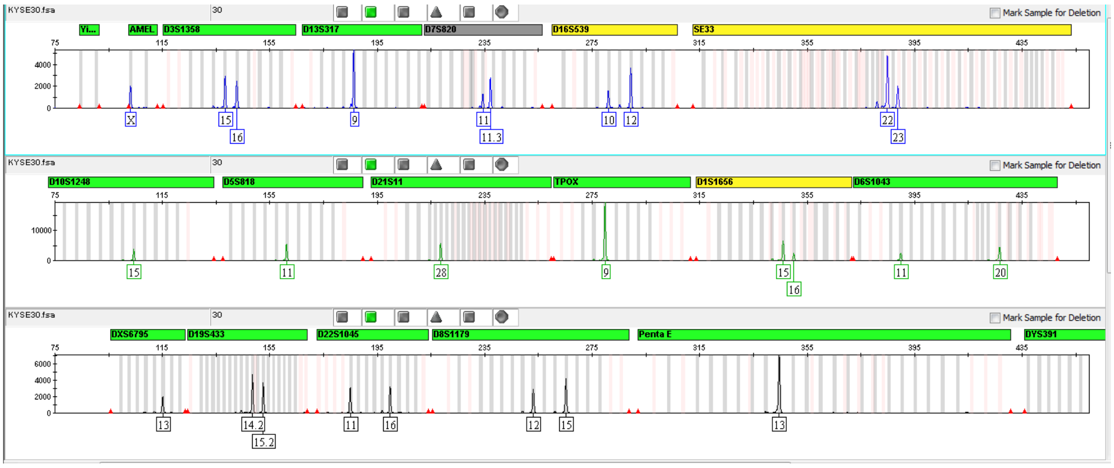

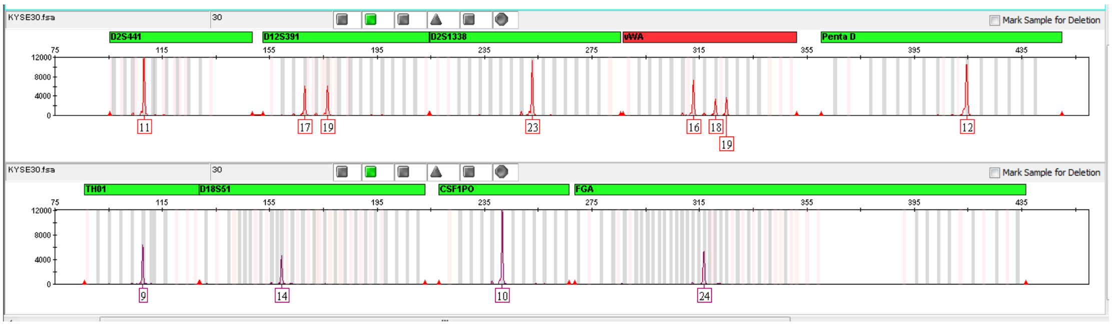

3.4 KYSE140 检测结果

3.4.1 KYSE140 STR 数据

STR 基因座	KYSE140 检测结果	
	Allele 1	Allele 2
Yindel	-	
AMEL	X	
D3S1358	18	
D13S317	12	
D7S820	10	11
D16S539	10	12
SE33	24.2	28.2
D10S1248	14	15
D5S818	10	
D21S11	29	
TPOX	8	
D1S1656	17	
D6S1043	11	14
DXS6795	11	
D19S433	13	15
D22S1045	16	17
D8S1179	10	
Penta E	16	17
DYS391	-	
D2S441	12	
D12S391	17	19
D2S1338	17	
vWA	14	
Penta D	9	
TH01	7	9
D18S51	14	
CSF1PO	13	
FGA	22	23

3.4.2 KYSE140 STR 位点数据比对结果

通过 DSMZ 数据库/客户提供细胞信息比对结果如下

EV	Cell No.	Cell name	Locus names								
			D5S818	D13S317	D7S820	D16S539	VWA	TH01	AM	TPOX	CSF1PO
	Query (Your Cell)		10,10	12,12	10,11	10,12	14,14	7,9	X,X	8,8	13,13
0.94(34/36)	348	KYSE-140	10,10	12,12	10,10	10,12	14,14	7,9	X,X	8,8	13,13
0.72(26/36)	RCB1293	SSP-25	10,10	12,12	11,11	10,12	14,17	7,9.3	X,X	8,8	12,12
0.67(24/36)	HTB-105	Tera-1	11,13	12,12	9,10	10,12	14,14	7,8	X,X	8,8	11,11
0.67(24/36)	JCRB1011	KMRC-2	11,14	12,12	10,11	9,11	14,18	7,9	X,X	8,8	11,13
0.67(24/36)	JCRB1317	KCL-22	10,11	8,12	11,12	12,12	14,14	7,9	X,X	8,8	12,12
**1.00(36/36)**	**CVCL_1347(Cosmic-CLP)**	**KYSE-140**	**10,10**	**12,12**	**10,11**	**10,12**	**14,14**	**7,9**	**X,X**	**8,8**	**13,13**
**0.94(34/36)**	**CVCL_1347(DSMZ)**	**KYSE-140**	**10,10**	**12,12**	**10,10**	**10,12**	**14,14**	**7,9**	**X,X**	**8,8**	**13,13**

客户提供数据比对

根据客户提供数据进行比对，KYSE140 样本的 STR 结果与数据相符。

注：参考 ANSI/ATCC,Authenticationof Human Cell Line Standardization of STR Profiling . 2011,ASN-0002-2011.标准，STR 检测匹配度超过 80%即可认为是同一种来源的细胞。

3.4.3 KYSE140 STR 图谱

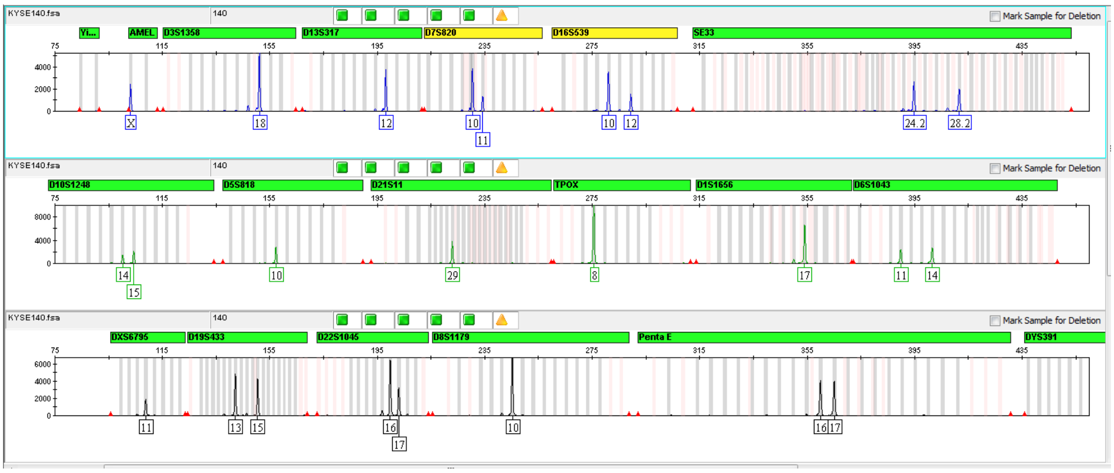

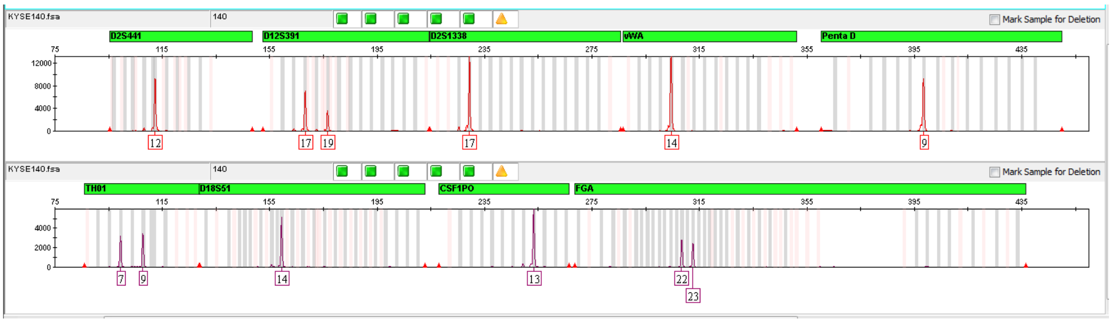

3.5 KYSE510 检测结果

3.5.1 KYSE510 STR 数据

STR 基因座	KYSE510 检测结果	
	Allele 1	Allele 2
Yindel	-	
AMEL	X	
D3S1358	17	
D13S317	12	
D7S820	11	12
D16S539	9	
SE33	31.2	
D10S1248	13	14
D5S818	11	
D21S11	31	
TPOX	8	
D1S1656	13	
D6S1043	14	
DXS6795	13	
D19S433	15	
D22S1045	11	15
D8S1179	10	
Penta E	16	
DYS391	-	
D2S441	11	
D12S391	18	21
D2S1338	20	23
vWA	14	
Penta D	12	
TH01	9	
D18S51	15	
CSF1PO	11	13
FGA	22	

3.5.2 KYSE510 STR 位点数据比对结果

通过 DSMZ 数据库/客户提供细胞信息比对结果如下

EV	Cell No.	Cell name	Locus names								
			D5S818	D13S317	D7S820	D16S539	VWA	TH01	AM	TPOX	CSF1PO
	Query (Your Cell)		11,11	12,12	11,12	9,9	14,14	9,9	X,X	8,8	11,13
1.00(36/36)	374	KYSE-510	11,11	12,12	11,12	9,9	14,14	9,9	X,X	8,8	11,13
0.94(34/36)	374	KYSE-510	11,11	12,12	11,12	9,9	14,14	9,9	X,X	8,8	11,11
0.72(26/36)	CRL-2072	CCD-1059Sk	11,11	12,12	11,12	9,11	16,18	9,9.3	X,X	8,8	11,12
0.72(26/36)	JCRB0816	SBC-1	9,11	12,12	11,12	9,9	14,17	8,9	X,X	8,8	10,12
0.72(26/36)	JCRB0817	SBC-2	9,11	12,12	11,12	9,9	14,17	8,9	X,X	8,8	10,12
**1.00(36/36)**	**CVCL_1354**	**KYSE-510**	**11,11**	**12,12**	**11,12**	**9,9**	**14,14**	**9,9**	**X,X**	**8,8**	**11,13**

客户提供数据比对

根据客户提供数据进行比对，KYSE510 样本的 STR 结果与数据相符。

注：参考 ANSI/ATCC,Authenticationof Human Cell Line Standardization of STR Profiling . 2011,ASN-0002-2011.标准，STR 检测匹配度超过 80%即可认为是同一种来源的细胞。

3.5.3 KYSE510 STR 图谱

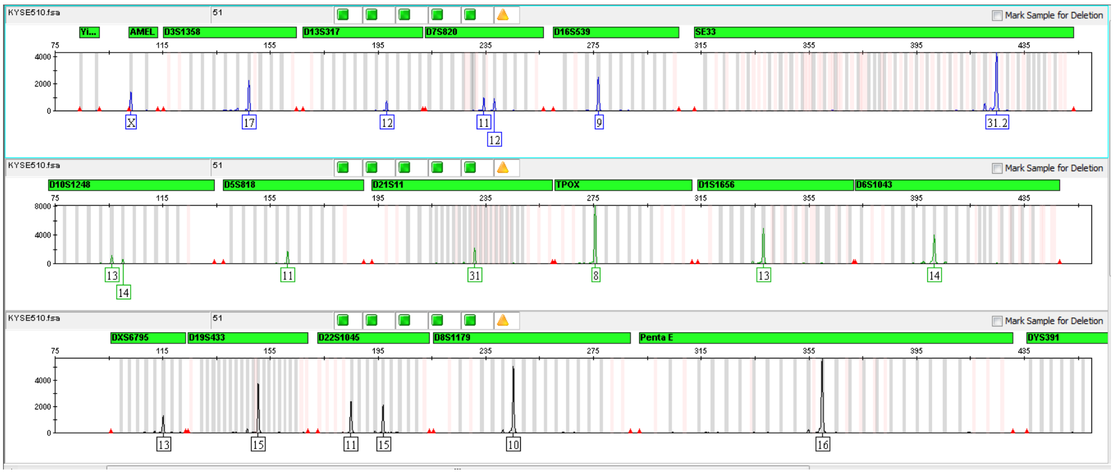

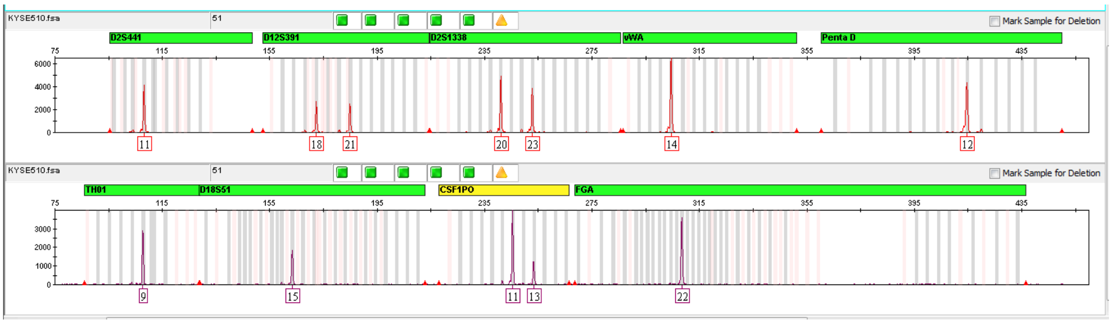

3.6 KYSE180 检测结果

3.6.1 KYSE180 STR 数据

STR 基因座	KYSE180 检测结果	
	Allele 1	Allele 2
Yindel	-	
AMEL	X	
D3S1358	15	
D13S317	9	12
D7S820	9	12
D16S539	9	10
SE33	20	21
D10S1248	13	15
D5S818	8	11
D21S11	30	
TPOX	11	
D1S1656	16	
D6S1043	11	
DXS6795	11	
D19S433	14	15.2
D22S1045	15	
D8S1179	10	12
Penta E	5	12
DYS391	-	
D2S441	10	
D12S391	18	19
D2S1338	27	
vWA	16	17
Penta D	9	
TH01	7	
D18S51	20	21
CSF1PO	12	13
FGA	19	21

3.6.2 KYSE180 STR 位点数据比对结果

通过 DSMZ 数据库/客户提供细胞信息比对结果如下

EV	Cell No.	Cell name	Locus names								
			D5S818	D13S317	D7S820	D16S539	VWA	TH01	AM	TPOX	CSF1PO
	Query (Your Cell)		8,11	9,12	9,12	9,10	16,17	7,7	X,X	11,11	12,13
1.00(36/36)	JCRB1083	KYSE180	8,11	9,12	9,12	9,10	16,17	7,7	X,X	11,11	12,13
0.94(34/36)	379	KYSE-180	8,11	9,12	12,12	9,10	16,17	7,7	X,X	11,11	12,13
0.76(28/37)	JCRB1082	KYSE170	11,12	9,9	11,12	9,10	16,17	7,9	X,X	11,11	10,12,13
0.67(24/36)	CRL-7657	Hs 907.Lu	11,12	12,12	8,10	9,10	16,17	7,7	X,X	8,8	12,13
0.67(24/36)	RCB0692	HTOA	11,13	8,10	9,10	9,13	17,17	7,7	X,X	11,11	12,13
**1.00(36/36)**	**CVCL_1349(JCRB)**	**KYSE-180**	**8,11**	**9,12**	**9,12**	**9,10**	**16,17**	**7,7**	**X,X**	**11,11**	**12,13**
**0.94(34/36)**	**CVCL_1349(DSMZ; Cosmic-CLP)**	**KYSE-180**	**8,11**	**9,12**	**12,12**	**9,10**	**16,17**	**7,7**	**X,X**	**11,11**	**12,13**

客户提供数据比对

根据客户提供数据进行比对，KYSE180 样本的 STR 结果与数据相符。

注：参考 ANSI/ATCC,Authenticationof Human Cell Line Standardization of STR Profiling . 2011,ASN-0002-2011.标准，STR 检测匹配度超过 80%即可认为是同一种来源的细胞。

3.6.3 KYSE180 STR 图谱

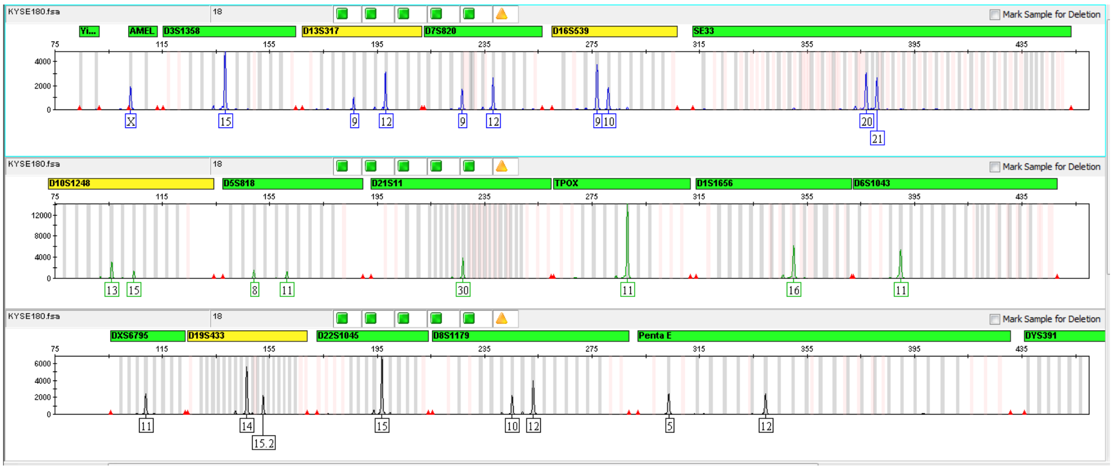

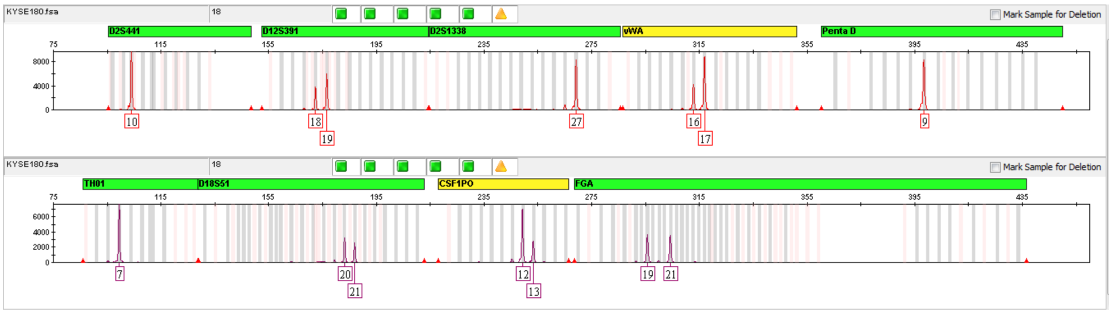

3.7 KYSE70 检测结果

3.7.1 KYSE70 STR 数据

STR 基因座	KYSE70 检测结果	
	Allele 1	Allele 2
Yindel	-	
AMEL	X	
D3S1358	15	
D13S317	11	12
D7S820	8	11
D16S539	13	
SE33	20	
D10S1248	14	
D5S818	12	
D21S11	30	
TPOX	8	
D1S1656	14	18
D6S1043	13	
DXS6795	13	
D19S433	13	15.2
D22S1045	15	17
D8S1179	12	
Penta E	11	
DYS391	-	
D2S441	11	
D12S391	19	24
D2S1338	16	
vWA	16	18
Penta D	9	
TH01	7	
D18S51	13	
CSF1PO	12	
FGA	21	

3.7.2 KYSE70 STR 位点数据比对结果

通过 DSMZ 数据库/客户提供细胞信息比对结果如下

EV	Cell No.	Cell name	Locus names								
			D5S818	D13S317	D7S820	D16S539	VWA	TH01	AM	TPOX	CSF1PO
	Query (Your Cell)		12,12	11,12	8,11	13,13	16,18	7,7	X,X	8,8	12,12
1.00(36/36)	363	KYSE-70	12,12	11,12	8,11	13,13	16,18	7,7	X,X	8,8	12,12
0.94(34/36)	JCRB0190	KYSE-70	12,12	11,12	8,11	13,13	16,18	7,11	X,X	8,8	12,12
0.72(26/36)	IFO50069	Alexander cells	12,12	11,12	9,11	13,13	15,16	7,8	X,X	8,8	10,10
0.72(26/36)	JCRB0406	PLC/PRF/5	12,12	11,12	9,11	13,13	15,16	7,8	X,X	8,8	10,10
0.72(26/36)	JCRB1090	NCE 16IIA	12,12	11,12	8,11	11,12	16,18	7,7	X,X	9,12	11,12
**1.00(36/36)**	**CVCL_1356**	**KYSE-70**	**12,12**	**11,12**	**8,11**	**13,13**	**16,18**	**7,7**	**X,X**	**8,8**	**12,12**

客户提供数据比对

根据客户提供数据进行比对，KYSE70 样本的 STR 结果与数据相符。

注：参考 ANSI/ATCC,Authenticationof Human Cell Line Standardization of STR Profiling . 2011,ASN-0002-2011.标准，STR 检测匹配度超过 80%即可认为是同一种来源的细胞。

3.7.3 KYSE70 STR 图谱

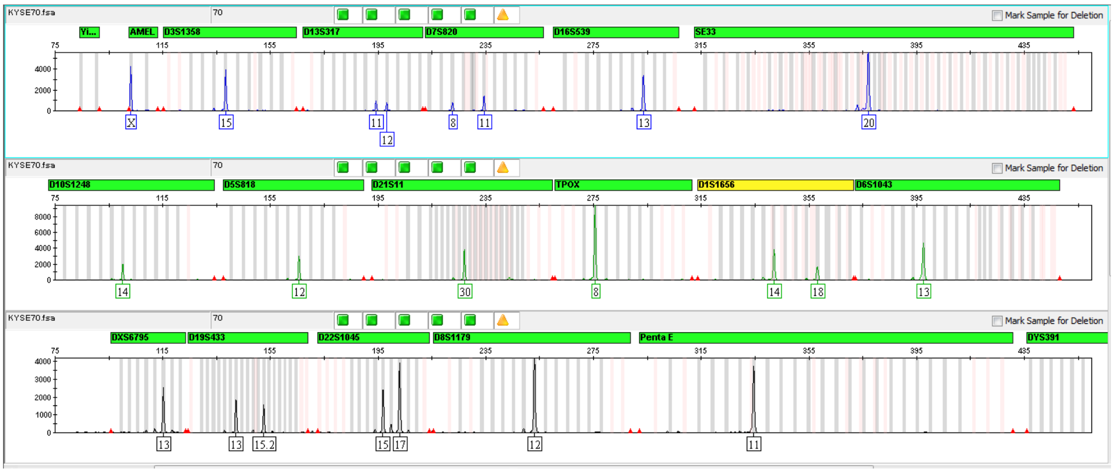

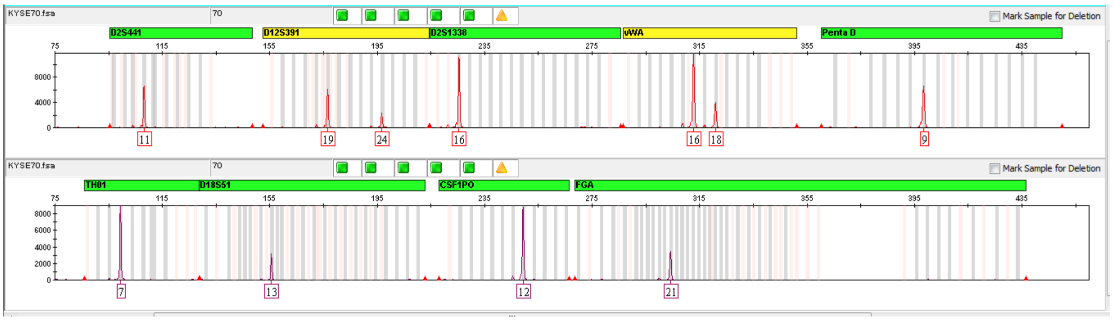

3.8 KYSE450 检测结果

3.8.1 KYSE450 STR 数据

STR 基因座	KYSE450 检测结果	
	Allele 1	Allele 2
Yindel	-	
AMEL	X	
D3S1358	18	
D13S317	11	
D7S820	11	12
D16S539	9	
SE33	19	20
D10S1248	12	14
D5S818	10	13
D21S11	30	
TPOX	8	
D1S1656	12	16
D6S1043	10	11
DXS6795	13	
D19S433	13	14.2
D22S1045	11	
D8S1179	10	
Penta E	11	16
DYS391	-	
D2S441	10	11
D12S391	22	
D2S1338	19	23
vWA	14	
Penta D	9	10
TH01	8	9
D18S51	13	
CSF1PO	11	12
FGA	24	

3.8.2 KYSE450 STR 位点数据比对结果

通过 DSMZ 数据库/客户提供细胞信息比对结果如下

EV	Cell No.	Cell name	Locus names								
			D5S818	D13S317	D7S820	D16S539	VWA	TH01	AM	TPOX	CSF1PO
	Query (Your Cell)		10,13	11,11	11,12	9,9	14,14	8,9	X,X	8,8	11,12
1.00(36/36)	387	KYSE-450	10,13	11,11	11,12	9,9	14,14	8,9	X,X	8,8	11,12
0.72(26/36)	JCRB1130	PL507	9,11	11,11	8,12	9,9	14,19	7,9	X,X	8,8	11,12
0.72(26/36)	RCB0153	HUC-F	10,10	8,11	11,13	9,9	14,14	8,9	X,X	8,9	11,13
0.67(24/36)	374	KYSE-510	11,11	12,12	11,12	9,9	14,14	9,9	X,X	8,8	11,11
0.67(24/36)	CRL-2073	NCCIT	10,13	11,11	10,10	9,12	14,18	7,9	X,X	8,8	10,12
**1.00(36/36)**	**CVCL_1353**	**KYSE-450**	**10,13**	**11,11**	**11,12**	**9,9**	**14,14**	**8,9**	**X,X**	**8,8**	**11,12**

客户提供数据比对

根据客户提供数据进行比对，KYSE450 样本的 STR 结果与数据相符。

注：参考 ANSI/ATCC,Authenticationof Human Cell Line Standardization of STR Profiling . 2011,ASN-0002-2011.标准，STR 检测匹配度超过 80%即可认为是同一种来源的细胞。

3.8.3 KYSE450 STR 图谱

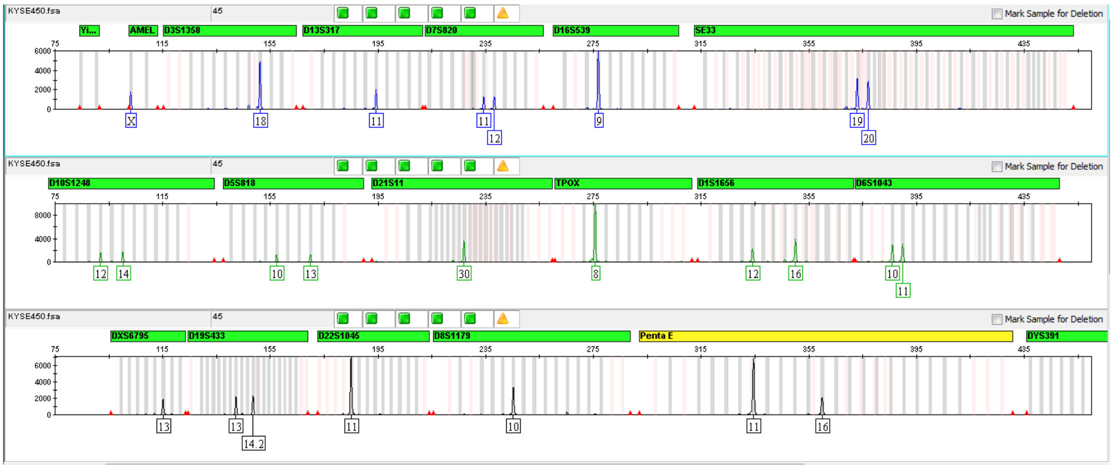

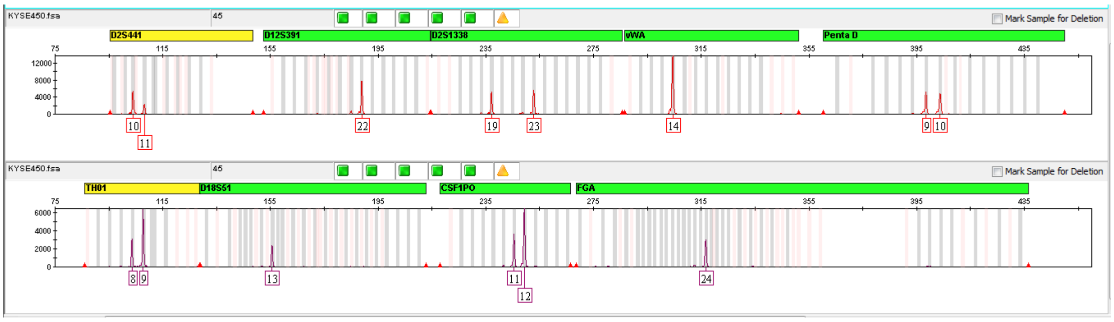

3.9 colo680n 检测结果

3.9.1 colo680n STR 数据

STR 基因座	colo680n 检测结果	
	Allele 1	Allele 2
Yindel	-	
AMEL	X	
D3S1358	15	
D13S317	13	
D7S820	10	12
D16S539	11	12
SE33	16	27.2
D10S1248	14	16
D5S818	11	
D21S11	27	
TPOX	6	
D1S1656	16	
D6S1043	12	19
DXS6795	12	
D19S433	16.2	
D22S1045	16	
D8S1179	14	15
Penta E	7	8
DYS391	-	
D2S441	11	
D12S391	17	18
D2S1338	23	
vWA	17	18
Penta D	12	
TH01	8	
D18S51	19	
CSF1PO	11	12
FGA	18.2	

3.9.2 colo680n STR 位点数据比对结果

通过 DSMZ 数据库/客户提供细胞信息比对结果如下

EV	Cell No.	Cell name	Locus names								
			D5S818	D13S317	D7S820	D16S539	VWA	TH01	AM	TPOX	CSF1PO
	Query (Your Cell)		11,11	13,13	10,12	11,12	17,18	8,8	X,X	6,6	11,12
0.94(34/36)	182	COLO-680N	11,11	13,13	10,12	11,12	17,18	8,8	X,X	6,8	11,12
0.72(26/36)	CRL-11233	THLE-3	13,13	13,13	8,10	11,12	17,18	8,9.3	X,X	6,9	11,12
0.61(22/36)	41	LP-1	11,11	12,12	11,12	11,12	17,17	7,8	X,X	11,11	11,12
0.61(22/36)	509	SUP-M2	11,11	10,11	8,12	11,12	16,18	8,9.3	X,X	8,10	11,12
0.61(22/36)	582	OCI-AML3	11,13	8,13	9,10	11,13	17,18	8,10	X,X	8,11	11,12
**1.00(36/36)**	**CVCL_1131(CLS)**	**COLO 680N**	**11,11**	**13,13**	**10,12**	**11,12**	**17,18**	**8,8**	**X,X**	**6,6**	**11,12**
**0.94(34/36)**	**CVCL_1131(Cosmic-CLP; DSMZ)**	**COLO 680N**	**11,11**	**13,13**	**10,12**	**11,12**	**17,18**	**8,8**	**X,X**	**6,8**	**11,12**

客户提供数据比对

根据客户提供数据进行比对，colo680n 样本的 STR 结果与数据相符。

注：参考 ANSI/ATCC,Authenticationof Human Cell Line Standardization of STR Profiling . 2011,ASN-0002-2011.标准，STR 检测匹配度超过 80%即可认为是同一种来源的细胞。

3.9.3 colo680n STR 图谱

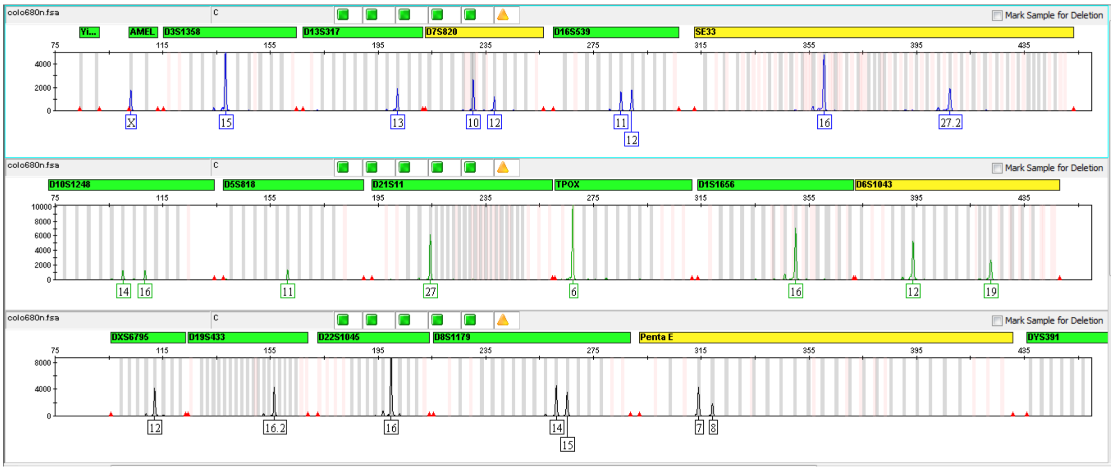

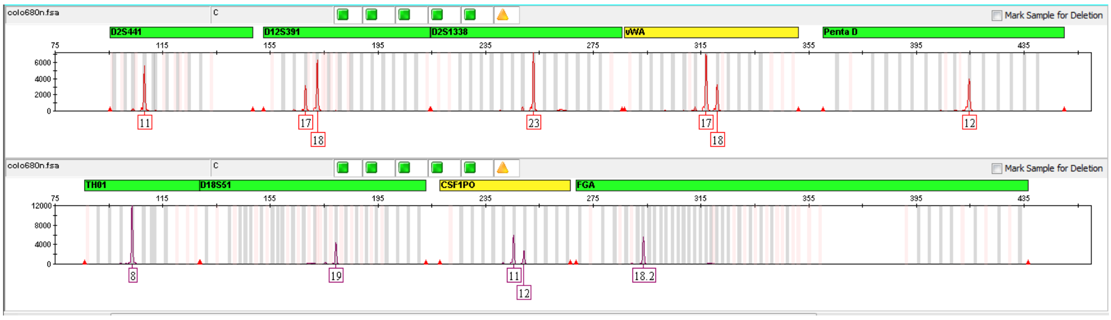

4. 参考文献

[1] Zhao, M., et al., Assembly and initial characterization of a panel of 85 genomically validated cell lines from diverse head and neck tumor sites. Clin Cancer Res, 2011. 17(23): p. 7248-64.

[2] Masters, J.R., Cell-line authentication: End the scandal of false cell lines. Nature, 2012. 492(7428): p. 186.

[3]American Type Culture Collection Standards Development Organization Workgroup, A.S.N., Authentication of Human Cell Lines: Standardization of STR Profiling. 2011, ANSI/ATCC ASN-0002-2011.

[4] Reid, Y.A., Characterization and authentication of cancer cell lines: an overview. Methods Mol Biol, 2011. 731: p. 35-43.

[5] Lorsch, J.R., F.S. Collins, and J. Lippincott-Schwartz, Cell Biology. Fixing problems with cell lines. Science, 2014. 346(6216): p. 1452-3.

[6] Chatterjee, R., Cell biology. Cases of mistaken identity. Science, 2007. 315(5814): p. 928-31.

NE2

GENETIC TESTING BIOTECHNOLOGY

Cell Line Authentication Service
STR Profile Report

**Email Address:**	liuhuijuan1025@163.com
**Sales Order:**	211022D
**Cell Line Designation:**	2-4
**Date Sample Received:**	Oct 22th, 2021
**Report Date:**	Oct 22th, 2021

Methodology: Nineteen short tandem repeat (STR) loci plus the gender determining locus, Amelogenin, were amplified using the commercially available EX20 Kit from AGCU. The cell line sample was processed using the ABI Prism® 3130 XL Genetic Analyzer. Data were analyzed using GeneMapper® ID v3.2 software (Applied Biosystems). Appropriate positive and negative controls were run and confirmed for each sample submitted.

Data Interpretation: Cell lines were authenticated using Short Tandem Repeat (STR) analysis as described in 2012 in ANSI Standard (ASN-0002) by the ATCC Standards Development Organization (SDO) and in Capes-Davis et al., Match criteria for human cell line authentication: Where do we draw the line? Int J Cancer. 2013;132(11):2510-9.

GTB™ performs STR Profiling following ISO 9001:2008 and ISO/IEC 17025:2005 quality standards.

There are no warranties with respect to the services or results supplied, express or implied, including, without limitation, any implied warranty of merchantability or fitness for a particular purpose. Genetic Testing Biotechnology (GTB) is not liable for any damages or injuries resulting from receipt and/or improper, inappropriate, negligent or other wrongful use of the test results supplied, and/or from misidentification, misrepresentation, or lack of accuracy of those results. Your exclusive remedy against GTB and those supplying materials used in the services for any losses or damage of any kind whatsoever, whether in contract, tort, or otherwise, shall be, at GTB's option, refund of the fee paid for such service or repeat of the service.

NOTE: According to the recommendations of *IJC* on cell line authentication, the report is valid for 3 years since the issue date.

Technical Questions?
GTB Technical Support
+86-512-67486171
service@jsdna.org
Section 505, Yixin BLD
SIP, Suzhou, 215123
Jiangsu, P.R. China

Ordering Questions?
order@jsdna.org
GTB Corporation
+86-512-62806339
Section 303, Yixin BLD
SIP, Suzhou, 215123
Jiangsu, P.R. China

GENETIC TESTING BIOTECHNOLOGY

Cell Line Authentication Service
STR Profile Report

Sales Order: 211022D

	Test Results for Submitted Sample		DSMZ Reference Database Profile
**Loci**	**Query Profile:** 2-4		**Database Profile:** NA
Amelogenin	X	Y	
D3S1358	14	18	
D13S317	9	12	
D7S820	12		
D16S539	9	11	
Penta E	12	20	
TPOX	9	11	
TH01	6	7	
D2S1338	19	24	
CSF1PO	10	11	
Penta D	9	12	
D19S433	13	14	
vWA	14	15	
D21S11	30	31	
D18S51	18		
D6S1043	14	19	
D8S1179	10	11	
D5S818	12		
D12S391	19	23	
FGA	21	23	

The allele match algorithm compares the 8 core loci plus amelogenin only, even though alleles from all loci will be reported when available.

Note: Loci highlighted in grey (8 core STR loci plus Amelogenin) can be made public to verify cell identity. In order to protect the identity of the donor, ***please do not publish*** *the allele calls from all the STR loci tested. The sample match is based on the reference data available at the time of comparison.*

Explanation of Test Results

Cell lines with ≥80% match are considered to be related; i.e., derived from a common ancestry. Cell lines with between a 55% to 80% match require further profiling for authentication of relatedness.

- [x] The submitted sample profile is human, but not a match for any profile in the DSMZ STR database.
- [ ] The submitted profile is an exact match for the following human cell line(s) in the DSMZ STR database (8 core loci plus Amelogenin):
- [ ] The submitted profile is similar to the following DSMZ human cell line(s):

e-Signature, Technician: Digitally signed by Xuekun Chen
DN: cn=Xuekun Chen, o=Genetic Testing Biotechnology (Suzhou), ou=DNA Typing Section, email=order@jsdna.org, c=CN
Date: 2021.10.22 17:49:23 +08'00'

GENETIC TESTING BIOTECHNOLOGY — GUARANTEE — CELL LINE AUTHENTICATION — ★★★★★

e-Signature, Reviewer: Digitally signed by Xiankun Zhao
DN: cn=Xiankun Zhao, o=Genetic Testing Biotechnology (Suzhou), ou=Supervision Section, email=service@jsdna.org, c=CN
Date: 2021.10.22 17:52:54 +08'00'

Addendum: Electropherogram for the customer's sample set 1 of 1

Cell Line Authentication Service
STR Profile Report

GENETIC TESTING BIOTECHNOLOGY

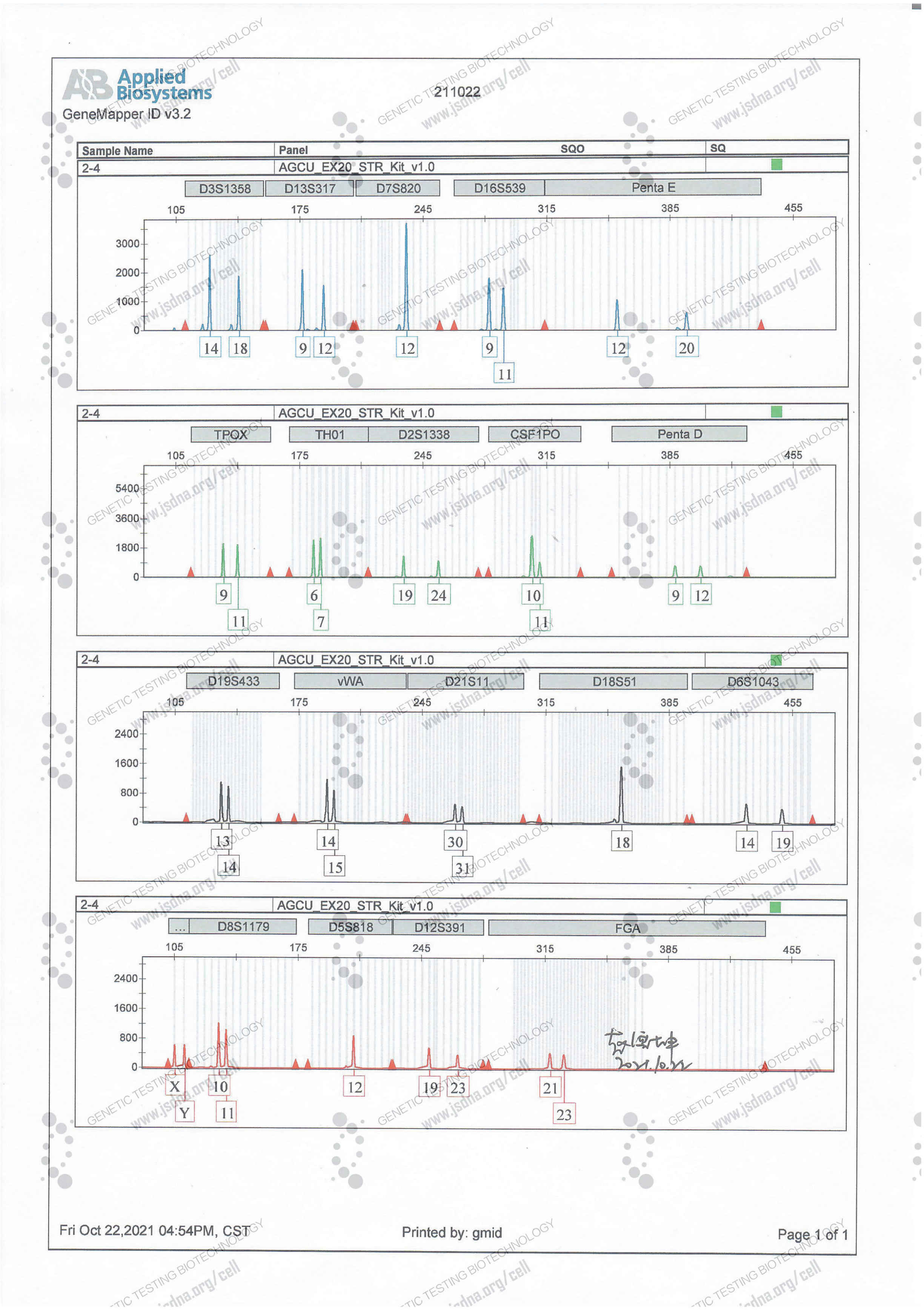

Supplement: Supplementary file 4 — Supporting Information [file ADVS-13-e09574-s001.pdf]
